# Probabilistic estimation of the source component of seismic hazard in North-Eastern Brazil

**DOI:** 10.1016/j.heliyon.2024.e30716

**Published:** 2024-05-03

**Authors:** J.A.S. Fonsêca, S. Lasocki, A.F. do Nascimento

**Affiliations:** a Federal University of Rio Grande do Norte, Departament of Geophysics, Natal, RN, 59078-970, Brazil; b Institute of Geophysics Polish Academy of Sciences, Warsaw, Poland

**Keywords:** Seismic hazard, Statistical seismology, Stable continental region

## Abstract

Stable continental regions pose unique challenges for conducting Probabilistic Seismic Hazard Analysis because the earthquake activity driving mechanisms are poorly understood. For instance, the lower seismicity (hence the paucity of data) and the absence of well-defined active fault systems complicate accurately determining seismic source parameters. Northeastern Brazil is a stable continental region exhibiting moderate-size events recorded with significant seismic intensities and provoking the collapse of poorly constructed buildings in the last century. Thus, assessing the seismic hazard is critical for seismic risk mitigation. The seismic hazard depends on three components: source, path, and site, and here, we present the probabilistic seismic hazard analysis of the source component for NE Brazil. Spatial aggregation of earthquake sources outlined four areal seismic zones. A goodness-of-fit test rejected the Gutenberg-Richter model of magnitude frequency distribution in one of the studied seismic zones. For this reason, we estimated the magnitude probability distribution function in that zone using a nonparametric adaptive kernel estimator. In other zones the Gutenberg-Richter magnitude frequency model was applied. In either way of the magnitude probability distribution modelling we considered the upper bound for magnitude equal to 6.6 *m*_*R*_, based on the upper bound of a 95 % confidence interval for the standard normal distribution of palaeoearthquake sizes. Our findings suggests that potentially damaging events are likely to occur, and we cannot neglect chances for the occurrence of earthquakes exceeding 5.2 *m*_*R*_. The calculated mean return periods indicate significantly shorter intervals between consecutive large events than palaeoseismic records.

## 1 Introduction

The seismic hazard is associated with a physical phenomenon resulting from an earthquake, e.g., ground shaking or surface faulting. When combined with buildings' vulnerability and exposure, the characterization of seismic hazards is a critical input for estimating seismic risk [1].

Seismic hazard depends on three components: source, path, and site [2]. The source component relates the size- and space-time occurrences of events, and path and site components represent how the seismic waves taking off from the source interact with the rock mass until they reach an observation point.

As a result, the seismic hazard analysis quantifies ground motion intensity for a place of interest [2]. One way of assessing it is to define the most pessimistic scenario regarding the maximum source located at the least possible distance to the place of interest. This approach is the Deterministic Seismic Hazard Analysis (DSHA). Although DSHA has been widely employed (e.g. Refs. [[3], [4], [5]]), it faces significant problems when the uncertainties far exceed the knowledge of seismic hazard components. The characterization of an extreme event and its respective ground motion intensity behaviour along the medium is not obvious and depends on the scrutinization of many important parameters and their interrelationship [6]. For example, in the case of multiple sources, there may not exist a single maximum event that could promote an extreme scenario covering all frequencies of ground shaking [7]. This is a more prominent problem in low seismicity areas, e.g., most intraplate regions, because of the usual lack of well-defined earthquake sources and its poor correlation with surface geologic features [8,9].

Another way of assessing seismic hazard is incorporating all possible events and their respective ground intensities, employing a probabilistic framework [7]. It represents the Probabilistic Seismic Hazard Analysis (PSHA), which uses the space, time, and size distributions of earthquakes available for a region of interest. The result is expressed as the exceedance probability of specified levels of ground motion at a specified place within a specific time window. PSHA has gained widespread application across different tectonic environments (e.g. Refs. [[10], [11], [12]]). The uncertainties are incorporated into PSHA and quantified as aleatoric (inherent to the random component of the process) and epistemic (inherent to the lack of knowledge of the process) uncertainties. Although it is impossible to reduce aleatoric uncertainty, one can reduce epistemic uncertainty by improving seismicity knowledge and increasing PSHA assessment accuracy. In this regard, proper seismic source characterization is crucial in advancing the PSHA accuracy [[13], [14], [15]]. PSHA has traditionally focused on estimating the fraction of Earth's gravitational acceleration, 'g', that can be exceeded at a specific site of interest [7]. It integrates the intricate interplay of earthquake sources, seismic paths, and site effects, providing a holistic view of seismic hazard.

Most of the Brazilian territory lies in South America's Stable Continental Region (SRC), whose seismicity is one of the least active in the world [16]. Still, its crustal portion has documented and observed tectonic earthquakes with significant magnitudes. The strongest recorded event reached the magnitude of 6.2 in Central Brazil in 1955, with the felt area of up to 500 km [16,17]. An even larger earthquake likely happened in 1690 with a magnitude of 7.0 in the Amazon region, but there is not enough information to confirm it [18]. However, moderate-size events (M ∼ 5) have been relatively frequently recorded and generated noticeable effects, since light damage to the collapse of ill-constructed buildings [16].

In the published literature, seismic hazard analysis for Brazil has been commonly addressed on a global scale (e.g. Refs. [[19], [20], [21], [22]]). Within these studies the currently known seismic zones have not been addressed individually. The reasons for not addressing individually known seismic zones in Brazil are twofold: 1) these global-scale studies used datasets containing only stronger events collected from international catalogues that did not depict sources zones from smaller earthquakes that might pose a significant hazard, and 2) with the improvement of seismic monitoring in Brazil, new seismic zones were identified only in the last decades. In any case, [23] developed preliminary studies of PSHA for Brazil considering a regional dataset and included more data. Also, [24] conducted other assessments for specific areas, such as the continental margin of the Southeast region, and [25] for a nuclear power plant.

**Fig. 1:**
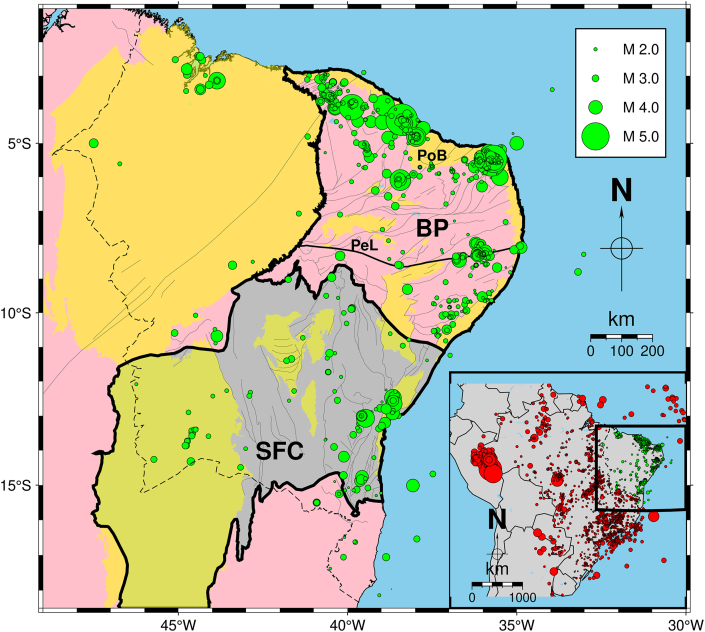
Filtered SISBRA events plotted over simplified geologic map of NE Brazil and main tectonic units [54]. The dashed black line delimits the NE Brazil area, while green circles represent the selected events, with their sizes corresponding to the magnitudes of the earthquakes. The legend on the map indicates the magnitude scale. Pink areas represent Proterozoic Fold belts, where a thicker black line outlines the Borborema Province (BP). In the BP lies the Pernambuco Lineament (PeL), indicated by the E-W oriented thick black line. Yellow areas indicate sedimentary covers in which the Potiguar Basin (PoB) is identified. The São Francisco Craton (SFC) is indicated by the grey area outlined by a thicker black line. Inset: seismicity map of Brazil. Only precise events (see text for explanation) from the SISBRA catalogue are plotted. The black dashed line represents NE Brazil limits. Epicentres located outside the study area are red-coloured. Green circles symbolize the remaining catalogue. (For interpretation of the references to colour in this figure legend, the reader is referred to the Web version of this article.)

The Northeastern part of Brazil is one of the most seismic active areas of the SCR of South America, where its seismicity is characterized by frequent seismic swarms [9,16]. Another characteristic of this region is that aftershock sequences can last several years [16]. The maximum earthquake recorded in NE Brazil was a 5.2 mb in 1980 that caused severe damage near the epicentre zone (city of Cascavel, in the NW part of the Potiguar Basin, PoB, Fig. 1). This earthquake was felt up to 600 km away, followed by a 2-year sequence of aftershocks [26,27]. Similarly, near the city of João Câmara (in the E PoB, Fig. 1), recurrent aftershocks between 1986 and 1989 with mainshocks of 5.1 mb and 5.0 mb provoked the collapse of many buildings and escape of population from nearby urban centres [[28], [29], [30]].

Several works have studied seismogenic sources and helped to delimit seismic zones in NE Brazil (e.g. Refs. [[27], [28], [29],[31], [32], [33], [34], [35], [36], [37], [38], [39], [40], [41]]). Conversely, few studies have assessed seismic hazard, limiting the analysis for specific areas [42,43].

This work presents NE Brazil's PSHA source component analysis results, considering the most recent Brazilian earthquake catalogue. The data preparation comprised time distribution analysis, completeness magnitude estimation, and seismicity declustering. Next, we tested the adopted probabilistic model for magnitude. Finally, we estimated the seismic hazard parameters: the maximum credible magnitude, mean return period, and exceedance probability, for the four most active seismic zones.

## 2 Regional geology and seismotectonic setting

Fig. 1 shows a simplified geologic map of NE Brazil plotted with earthquakes collected from the Brazilian Seismic Catalog (Catálogo Sísmico Brasileiro - SISBRA) and the surface-mapped tectonic faults and ductile shear zones. This region encompasses the dynamic interplay of the Borborema tectonic province (BP, Fig. 1), the São Francisco Craton (SFC, Fig. 1), and various phanerozoic sedimentary basins.

BP, formed during the Proterozoic in the Brasiliano Cycle, exhibits complex geology, where key structural features include NE-oriented fold belts and EW-oriented shear zones [44]. The BP's geological domains extend into African metamorphic terranes, presenting a diverse composition of Neoproterozoic supracrustal belts, Paleoproterozoic gneissic-migmatitic basement sequences, and Archean nuclei [45].

The SFC basement presents subalkaline granitoids dating to approximately 1700 Ma [46]. Subsequent to this period, the overlying sediments underwent deformation during the ∼600 Ma Brasiliano orogeny, characterized by the presence of westward-inclined ductile shear zones [47]. These zones extend southward into adjacent geological units.

In NE Brazil, earthquake sources have been recognized as seismically defined faults ranging from a few hundred meters to tens of kilometres in length. Mostly, such seismic sources do not coincide with surface-mapped geologic structures [9]. So far, the only example of fault reactivation was found in the East of the Pernambuco Lineament (PeL, Fig. 1), a ductile shear zone whose 700 km long, E-W oriented main belt links to secondary NE oriented branches [9,48]. Furthermore, NE Brazil events occur in shallow depths (up to 12 km), where most concentrate along the continental margin, mainly near areas where geophysical contrasts are found (e.g., sedimentary basins borders) [9,35,49]. Such areas are considered weak zones, where superposition of local and regional stresses play a crucial role in generating seismicity [[33], [34], [35],^50^]. Their spatial aggregation of earthquake sources outlines areal seismic zones. In this context, the most active tectonic domain is the BP [9]. In BP, four main seismogenic zones, whose seismicity rates are the highest in NE Brazil, have been identified as producing significant magnitude events: 1) Acaraú, in the NW BP (Fig. 1) (e.g. Refs. [32,35,36,40]), 2) the NW PoB border (Fig. 1), where the largest NE Brazil earthquake occurred (e.g. Refs. [27,34,35]); 3) the E PoB border, where the João Câmara earthquake sequences were recorded (Fig. 1) (e.g., Refs. [29,30,35]; 4) The reactivated part of PeL (Fig. 1) (e.g. Refs. [33,37,39,48]). Notwithstanding, other areas also present significant magnitude event occurrence. In this regard, one can highlight the Northwestern part of NE Brazil and SFC, where the largest earthquakes were recorded at 4.3 *m*_*R*_ in 2017 [51] and 4.5 *m*_*R*_ in 2020 [52,53], respectively. However, seismicity in these two regions is low, as is the number of characterized seismogenic faults.

## 3 Methodology and data

### 3.1 Methodology

We used three methods for estimating the magnitude of completeness (Mc): 1) Maximum curvature (MAXC) [55]; 2) goodness-of-fit test (GFT) [55]; 3) modified goodness-of-fit test (MGFT) [56]. We used the *Completeness Magnitude estimation* application on the EPISODES platform (https://episodesplatform.eu; [57]). Then, we applied two declustering methods: 1 - [58] (G-K), and 2 - [59] (R) in our datasets and compared their results.

We performed time-independent PSHA, assuming that the seismic process does not change in time. Hence, its probabilistic characteristics, estimated from past data, are also the characteristics of this process in the future. In this regard, earthquake occurrence rate and size distributions are key elements. Their interplay characterizes the PSHA as the probability distribution of event magnitude (M), conditional upon event occurrence in D time units

(1) $$f\left(M|N\left(D\right)\neq 0\right)=\frac{f\left(M\right)}{1-\mathrm{Pr}\left[N\left(D\right)=0\right]}\sum_{n=1}^{\infty }n\;\mathrm{Pr}\left[N\left(D\right)=n\right]{\left[F\left(M\right)\right]}^{n-1}$$

where F(M) and f(M) are the Cumulative Distribution Function (CDF) and Probability Density Function (PDF) of magnitude. Pr[N(D)=n] is the probability distribution of event occurrence frequency.

We modelled earthquake occurrence frequency by the Poisson distribution, which results from the assumption that the event occurrence in time is a Poisson process. Equation (2) shows the Poisson distribution: expressed as the probability of n event occurrence in *D* time units

(2) $$\mathrm{Pr}\left[N\left(D\right)=n\right]=\frac{{\left(\lambda D\right)}^{n}}{n!}e^{-\lambda D}$$

where λ is the rate of earthquake occurrence, estimated as the total number of events with magnitude ≥ Mc divided by the time interval in which these events were observed.

Incorporating equation (2) into equation (1) makes conditional PDF

(3) $$f\left(M|N\left(D\right)\neq 0\right)=\frac{\lambda Df\left(M\right)e^{-\lambda D\left[1-F\left(M\right)\right]}}{1-e^{-\lambda D}}$$

Equation (3), therefore, represents the probability distribution of event magnitude (M), conditional upon event occurrence in D time units assuming a Poisson process. The seismic hazard is often parameterized by:

(4) $$R\left(M_{p},D\right)=1-\;e^{-\lambda D\left[1-F_{M}\left(M_{p}\right)\right]}$$

(5) $$T\left(M_{p}\right)={\left\{\lambda \left[1-\;F_{M}\left(M_{p}\right)\right]\right\}}^{-1}$$

(6) $$T\left(M_{cred}\right)=D$$

where $R\left(M_{p},D\right)$, is the exceedance probability of the event of magnitude $M_{p}$ within *D* time units, $T\left(M_{p}\right)$ is the mean return period of such an event, and $M_{cred}$ is the maximum credible magnitude for *D* time units. In this work, we calculated these hazard parameters by using the following applications on the EPISODES platform (https://episodesplatform.eu [57];: *Stationary Hazard: Exceedance Probability*, *Stationary Hazard: Mean Return Period* and *Stationary Hazard: Maximum Credible Magnitude*.)

The level of seismic hazard is directly linked to the capacity to generate significant magnitude events. According to equations (4), (5), (6)), the more frequent the larger magnitude events, the higher the seismic hazard. We also notice that the hazard parameters depend on the magnitude CDF. Hence, accurate modelling of the magnitude CDF is crucial. Improper modelling results in either underestimation or overestimation of the seismic hazard. E.g., in a practical example, [60] showed that the mean return periods based on inappropriate Magnitude Frequency Distribution (MFD) strongly departed from observed earthquake recurrences.

In many PSHA studies, MFD have been based on the expectation that magnitude values ≥ Mc are log-linearly distributed, following the Gutenberg-Richter (G-R) law [61] as indicated by equation 7

(7) logN(m)=a−bm

where N(m) is the number of events whose magnitudes are ≥ m, a and b are constants, in which b explains the large-to-small proportion of events. The G-R law leads to the exponential distribution of magnitude, where its PDF and CDF are indicated by equations (8), (9)

(8) $$f\left(m\right)={\beta e}^{-\beta m}$$

(9) $$F\left(m\right)=1-e^{-\beta \left(m-Mc\right)}$$

where β=bln(10).

However, MFD have been observed in some cases to significantly deviate from the G-R model for global and regional catalogue data [[62], [63], [64], [65], [66], [67], [68]]. In this regard, testing on the appropriateness of such modelling is paramount for better-ensuring coherence with seismic hazard calculations, hence obtaining more reliable results. We used the Anderson-Darling (A-D) test [69] to verify if the G-R model can explain our set of MFD. We used the *Anderson-Darling test for magnitude distribution* application on the EPISODES platform (https://episodesplatform.eu; [57]) using 95 % significance level.

In the case of the exponentiality rejection, i.e., not adequacy of the G-R law in explaining MFD, the parametric distribution model for magnitude is unknown. One way to overcome this problem, [60,68,[70], [71], [72]] proposed using a model-free approach based on nonparametric kernel estimators to estimate the probability distribution functions of magnitude.

Since Mc is defined, any MFD has a lower bound fixed at Mc. However, both parametric and nonparametric magnitude models can be estimated with no upper limits or with upper bounds. We used upper bounded parametric and nonparametric models in the present work. As will be discussed later, the entire selected dataset spans a short time length of only 300 years. Therefore, we based our maximum magnitude ($M_{\max}$) estimate on palaeoseismic records presented by Refs. [9,73].

For magnitude CDF modelling, we used the *Source size distribution functions/Stationary Hazard* application on the EPISODES platform (https://episodesplatform.eu; [57]). The upper bounded exponential distribution of magnitude is

(10) $$F_{M}\left(M\right)=\frac{1-e^{-\beta \left(M-\;M_{c}\right)}\;}{1-e^{-\beta \left(M_{\max}-\;M_{c}\right)}\;}$$

where the maximum likelihood was used to estimate β [74,75].

We also used (e.g. Ref. [76], – and references therein) estimation method for the upper bounded nonparametric modelling of MFD using an adaptive form:

(11) $$\hat{F}\left(M\right)=\frac{\sum_{i=1}^{n}\left[\Phi \left(\frac{M-M_{i}}{\omega_{i}h}\right)-\Phi \left(\frac{Mc-M_{i}}{\omega_{i}h}\right)\right]}{\sum_{i=1}^{n}\left[\Phi \left(\frac{M_{\max}-M_{i}}{\omega_{i}h}\right)-\Phi \left(\frac{Mc-M_{i}}{\omega_{i}h}\right)\right]}$$

where $\hat{F}\left(M\right)$ is the cumulative distribution function of magnitude, *n* is the number of events whose magnitudes, $M_{i}$, are equal to or greater than Mc. h is the smoothing factor and $\omega_{i}$ are local bandwidths. Φ(x) is the standard Gaussian cumulative distribution.

### 3.2 Data

We collected data from the Brazilian Seismic Catalog (Catálogo Sísmico Brasileiro - SISBRA) for our analysis. This catalogue was prepared by the Seismology Centre of the University of São Paulo and comprises a period ranging from 1720 to 2020. It contains historical and instrumental earthquake information as a compilation of the original catalogue of [26] and collections produced by various seismology groups in Brazil (University of Brasília; Federal University of Rio Grande do Norte; The National Observatory; Institute for Technological Research; São Paulo State University; Federal University of Mato Grosso do Sul). The catalogue contains corrected information on historical earthquakes and data from old international catalogues. It also includes new seismic events, older data with revised parameters, and earthquakes from other countries felt within Brazilian territory.

**Fig. 2:**
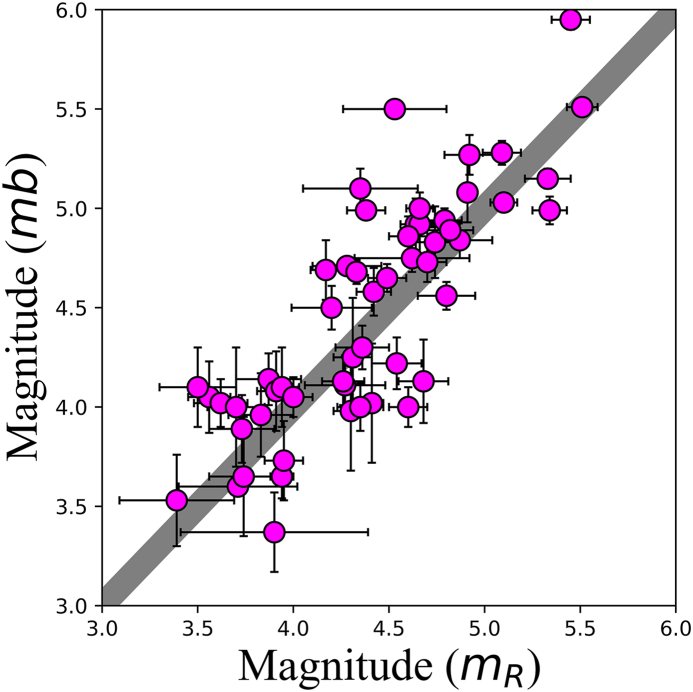
Relationship between mb and m_R_ scales for Brazilian earthquakes taken from Ref. [78]. The grey line indicates the 1:1 relation, and the error bars depict one standard deviation from the mean.

Our catalogue’s magnitudes are available in *m*_*R*_ [77] and *mb* scales. *m*_*R*_ is a Brazilian regional magnitude scale based on the P-wave train maximum amplitude of earthquakes within the distance range between 200 km and 1500 km, whose periods vary between 0.1 s and 1.0 s [77]. Fig. 2 shows the relationship between *mb* and *m*_*R*_ for Brazilian earthquakes taken from Ref. [78]. The mean values standard deviations are not significantly different from the 1:1 relation, indicating that both scales are equivalent [16,77,78]; thus, hereafter, we used *m*_*R*_ for all events in SISBRA.

We removed the earthquakes with location errors greater than 50 km and/or those for which the occurrence month and day were not provided. Then, we extracted all events in NE Brazil, so the remaining data that formed our dataset comprised 1329 events (Fig. 1 and Table S1 – see Supplementary materials for details).

**Fig. 3:**
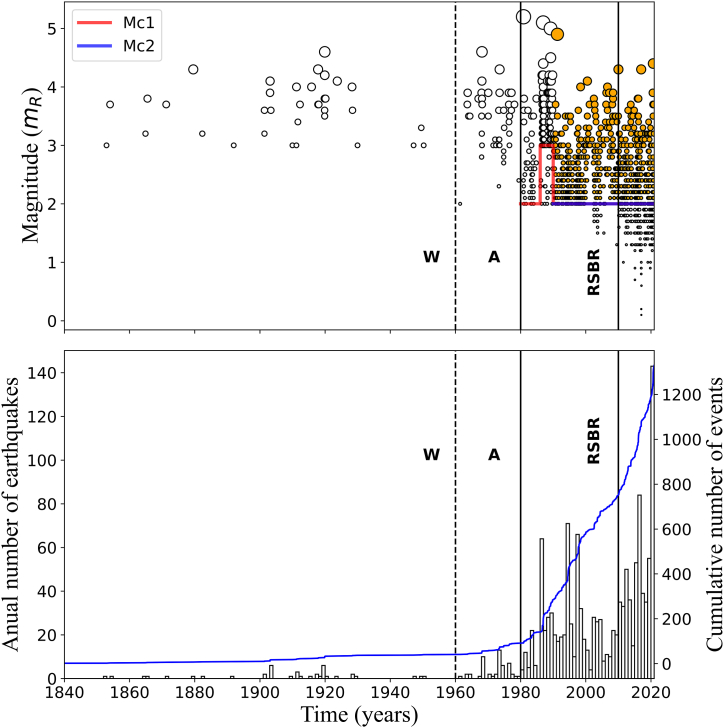
Temporal distribution of magnitude (upper panel) and annual frequency of earthquakes superposed with the cumulative number of earthquakes (lower panel) for the 1840–2020 catalogue time range. Letter W indicates the year 1960, the period that marks the start of WWSSN and the instrumental recording in Brazil. Letter A indicates the year 1980, which marks a significant lowering of event detection coincident with increasing station number. RSBR code denotes the start of the Brazilian Seismographic Network in 2010. Horizontal lines represent the magnitude of completeness estimations shown in Table 1 differenced by colour (red: from 1980 onwards, Mc1; blue: from 1990 onwards, Mc2). In the upper panel, earthquakes are symbolized by circles, where the selected ones after Mc estimation are orange coloured. (For interpretation of the references to colour in this figure legend, the reader is referred to the Web version of this article.)

**Fig. 4:**
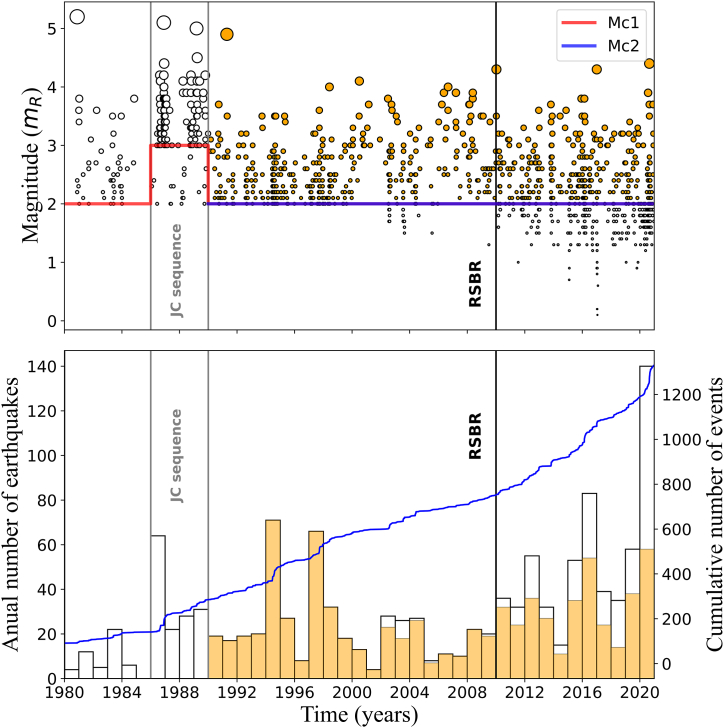
Same as Fig. 3 for the 1980–2020 catalogue time range. Vertical grey lines indicate the period between 1986 and 1989 when aftershocks near João Câmara city were recorded (JC sequence). Orange histograms are the annual frequency of earthquakes from the 1990–2020 catalogue with Mc ≥ 2.0. (For interpretation of the references to colour in this figure legend, the reader is referred to the Web version of this article.)

Although our NE Brazil catalogue began in 1720, only two earthquakes were reported until c. 1840. Fig. 3 shows a time-magnitude scatterplot from 1840. We can observe that the number of events reported in the catalogue changes over time because the level of magnitude detection varies over time. For instance, in the years 1960, 1980, and 2010 we can notice this effect as a sudden increase in the number of detected events (and hence, Mc decrease). [16,79] explain these changes in earthquake recording as the result of three distinct employment of seismographic stations in the world and Brazil. First, in the 1960s, the World-Wide Standardized Seismograph Network (WWSSN) started operation, and instrumental data began to be recorded in Brazil (indicated by 'W' in Fig. 3). Second, from around the 1980s, the number of stations in NE and SE Brazil increased (identified as 'A' in Fig. 3). Third, from 2010 onwards, a denser and permanent seismographic network, the Brazilian Seismographic Network (RSBR) [79], started operation in Brazil (indicated by the code RSBR in Fig. 3, Fig. 4).

Because data was relatively scarce before 1980 (Fig. 3), we selected the period from 1980 onwards to perform our analysis (Fig. 4). Between 1986 and 1989, two earthquakes with magnitudes 5.0+ followed by aftershocks occurred near João Câmara (JC) [28,29], as mentioned in the Introduction section. Both JC sequences of thousands of events occurred at the same seismogenic Samambaia fault. The NE Brazil catalogue contains only fractions of these sequences. Still, they strongly affect the time-magnitude distribution (JC sequence in Fig. 4).

The implementation of RSBR around 2010 (RSBR code in Fig. 3, Fig. 4) also changes the detectability of earthquakes. Therefore, we divided the catalogue into five periods of similar earthquake detectability levels: 1) from 1980 until 2009 (whole period); 2) from 1980 until 1985 (the period before aftershocks sequences period); 3) from 1986 until 1989 (the JC aftershock sequences period); 4) from 1990 until 2009 (post-JC aftershock sequences period); 5) from 2010 onwards (period of RSBR operation).

In Fig. 4, we observed that the yearly earthquake rate substantially increased, and it may be related to the COVID-19 transmission containment measures in Brazil throughout 2020 because: 1) anthropogenic noise reduction on the seismic records and 2) a higher scrutiny level of the manually analysed data. As a result, weaker event detection increased, and as will be shown later, this effect is negligible for PSHA estimation.

## 4 Results

We applied three magnitudes of completeness (Mc) estimation methods mentioned in Section 2 to each of the five sub-catalogues into which we divided our catalogue. We present the results in Table 1 (see Supplementary materials for details).

**Table 1:** Results of Mc estimation using MAXC, GFT, and MGFT methods for each divided by period sub-catalogue.

Sub-catalogue	Nº of events	MAXC	GFT	MGFT	Mc
1980–2009	660	2.0	3.0	2.0	2.0
1980–1985	49	2.0	2.0	2.0	2.0
1986–1989	145	3.0	3.6	3.0	3.0
1990–2009	466	2.0	2.0	2.0	2.0
2010–2020	578	2.0	2.0	2.0	2.0

Because MAXC and MGFT provided identical solutions for every sub-catalogue, we accepted these results as the final Mc values. They are shown in the “Mc” column of Table 1 and displayed in Fig. 3, Fig. 4 as horizontal red and blue lines (Mc1 and Mc2, respectively).

Mc = 2.0 was obtained in all sub-catalogues, except in the 1986–1989 sub-catalogue, where we obtained Mc = 3.0. The Mc estimates from all three methods were identical in sub-catalogues 1980–1985, 1990–2009, and 2010–2020.

To mitigate Mc uncertainties, we retained only the sub-catalogues where Mc estimates were the same. In addition, these sub-catalogues should be continuous in time. These criteria removed the datasets that contained only a few events or were influenced by aftershock sequences. As a result, we restricted the catalogue to the period from 1990 onwards (Mc2: blue horizontal line in Fig. 3, Fig. 4). The final dataset contained 803 events with magnitudes equal to or greater than Mc = 2.0 (orange circles in Fig. 3, Fig. 4). For such a dataset, the anomalous peak of events detected in 2020 (Fig. 4, lower panel), due to COVID-19 containment measures, was reduced to the levels typically observed in previous years (orange histograms in Fig. 3).

**Fig. 5:**
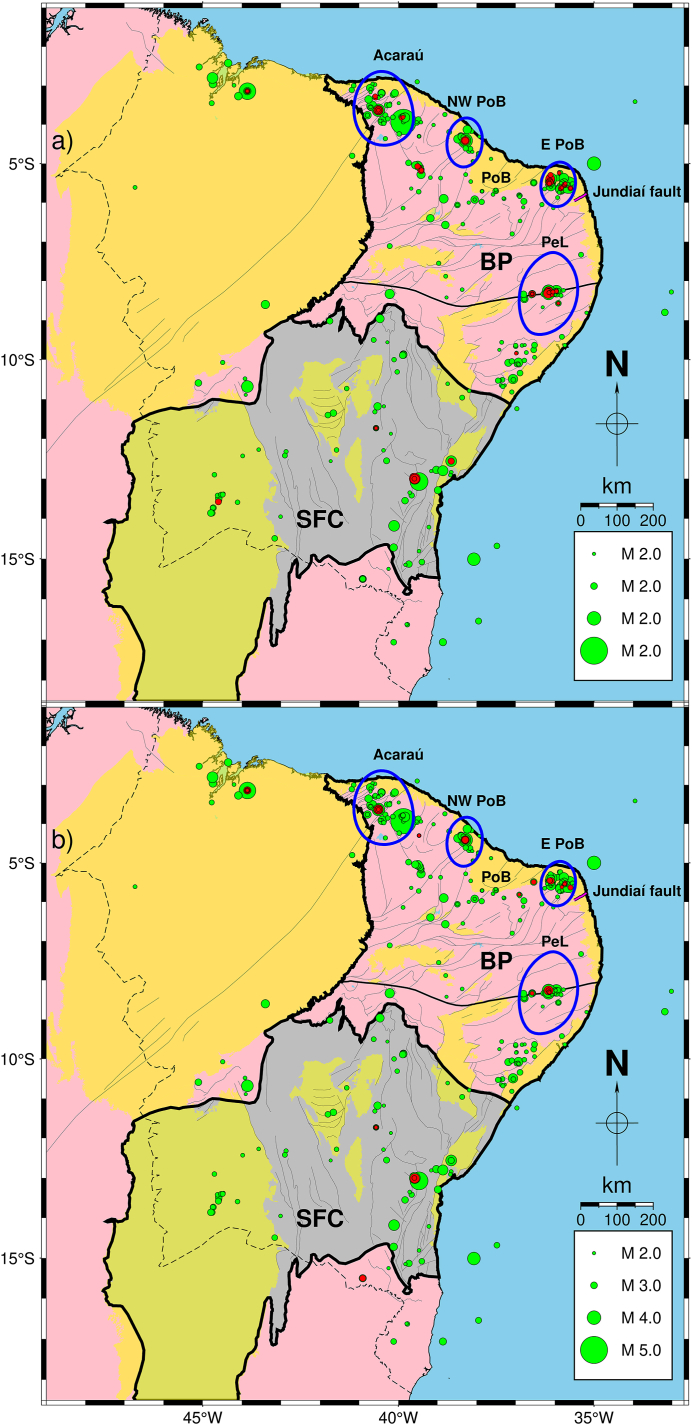
G-K (a) and R (b) seismicity declustering performance for the 1990–2020 sub-catalogue with Mc ≥ 2.0 plotted over the same map as displayed by Fig. 1. Red circles represent cluster events removed from the dataset while green circles indicate the remaining seismicity. Blue lines delimit NE Brazil's four most active source zones: Acaraú, NW PoB, E PoB, and PeL. The Jundiaí fault, used in this work for $M_{\max}$ estimation, is indicated by the magenta line. (For interpretation of the references to colour in this figure legend, the reader is referred to the Web version of this article.)

**Fig. 6:**
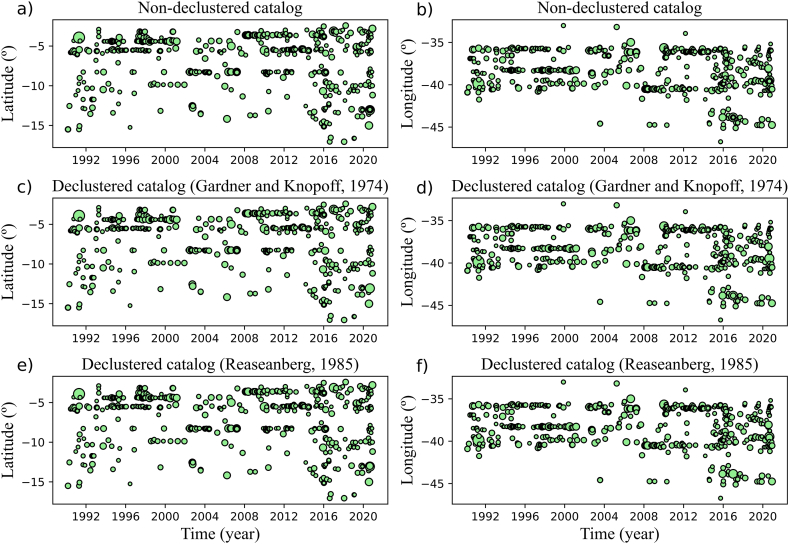
Latitude and longitude distributions between 1990 and 2020 for the non-declustered catalogue (a and b), G-K declustered catalogue (c and d), and R declustered catalogue (e and f).

We applied the G-K and R methods separately to the dataset resulted from the Mc estimation analysis (orange circles in Fig. 3, Fig. 4) for seismicity declustering. The G-K and R methods identified 200 and 75 dependent events, resulting in declustered datasets of 603 and 728 earthquakes, respectively. Figs. 5a and b shows the seismicity maps containing the declustered catalogue events (green circles) and removed events (red circles) for the G-K and R approaches, respectively. Figs. 6(a–f) show the latitude and longitude distributions along time for the non-declustered catalogue and declustered events after the G-K and R methods.

Dependent events location maps of both methods (Figs. 5a and b) were similar. In this regard, approximately 98 % of the mainshocks found by the G-K approach (590 events) were also found by the R method. Furthermore, the space-time distributions of the mainshocks found by the G-K (Figs. 6c and d) and R (Figs. 6e and f) methods were similar. Within the catalogues declustered by G-K and R, approximately 62 % and 68 % of seismicity concentrated in NE Brazil's four most active seismic zones, respectively. Blue lines in Figs. 5a and b delimit these zones. The remaining 38 % and 32 % of events were distributed as a more diffuse seismicity or formed small clusters.

Table 2 displays the numbers of non-declustered events and mainshocks found by the G-K and R methods within each seismic zone. As a result, the seismicity declustering following the G-K approach strongly reduced the size of datasets in three out of four seismic zones. Therefore, we accepted the dataset declustered by R once it was demonstrated to be less conservative. To further analyse the data for PSHA, we separated the four denser populated seismic zones.

**Table 2:** Number of events within each seismic zone before and after G-K and R seismicity declustering methods.

Seismic zone	Initial # of events	G-K	R
Acaraú	101	90	97
NW PoB	167	89	144
E PoB	158	117	145
PeL	116	75	106

We based $M_{\max}$ estimation on the palaeorecords discussed by Refs. [9,73]. These authors identified the largest Quaternary surface rupturing segment as long as 1.5 km in the Jundiaí fault (indicated in Figs. 5a and b) and suggested that it was equivalent to magnitude events as large as *Mw*5.5. The empirical relationships between different magnitude scales for Brazil [78] provided that *Mw* = 5.5 equals 5.6 in the *m*_*R*_ scale used in the present work. However, taking this value $M_{\max}$ might be biased because of the scarcity of the palaeorecords in NE Brazil and the entire country. To account for such uncertainty, we accepted $M_{\max}$ as the upper bound of the 95 % confidence interval for $M_{\max}$ assuming that it was normally distributed with the mean value 5.6 *m*_*R*_ and the standard deviation 0.5. As a result, we obtained $M_{\max}$ = 6.6 *m*_*R*_. We adopted this magnitude value as $M_{\max}$ in the MFD in the four seismic zones.

We tested the MFD of each seismic zone dataset, highlighted in Fig. 5b, using the A-D test with the significance level 95 % (α = 5 %). The results are summarized in Table 3 (see Supplementary materials for details). The A-D test result is represented by the mean p-value, calculated as the average value of 100 test trials. The standard deviations (SD) of each set of 100 repeated A-D tests are also provided.

**Table 3:** A-D test results for each selected seismic zone.

Seismic zone	Magnitude range (*m*_*R*_)	Mean p-value	SD
Acaraú	2.0–4.9	0.0186	0.005
NW PoB	2.0–4.1	0.8150	0.155
E PoB	2.0–4.3	0.2231	0.092
PeL	2.0–3.9	0.2211	0.075

The p-value for NW PoB, E PoB, and PeL zones was greater than α; hence, the exponential distribution model of magnitude, Eq. (10), was not rejected. This model was rejected for the Acaraú seismic zone, where the p-value was less than α. Based on the A-D tests results we used the parametric model, Eq. (10) to estimate the magnitude CDFs for the NW PoB, E PoB, and PeL zones, and the nonparametric adaptive kernel estimator (Eq. (11)) to model the Acaraú zone magnitude CDF.

**Fig. 7:**
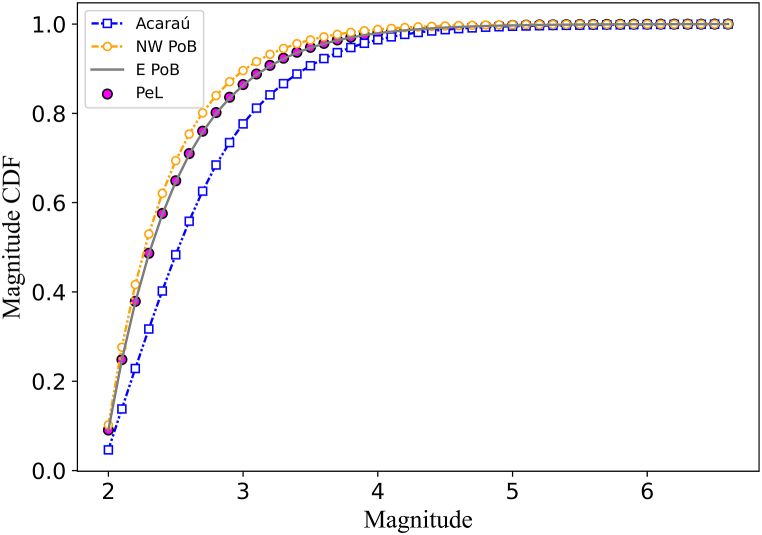
Cumulative Distribution Function of each seismic zone (color-coded as the legend indicates). (For interpretation of the references to colour in this figure legend, the reader is referred to the Web version of this article.)

For the exponential distribution model of magnitude for the NW PoB, E PoB and PeL seismic zones β (and b-value) estimations were equal to 2.15 (b = 0.94), 1.89 (b = 0.82) and 1.91 (b = 0.83), respectively. Fig. 7 displays the magnitude CDF-s for the four seismic zones.

We also estimated the mean activity rate λ for each seismic zone. For the Acaraú, NW PoB, E PoB, and PeL seismic zones, the λ estimates were equal to 3.28, 4.84, 4.81, and 3.63 events per year, respectively.

**Fig. 8:**
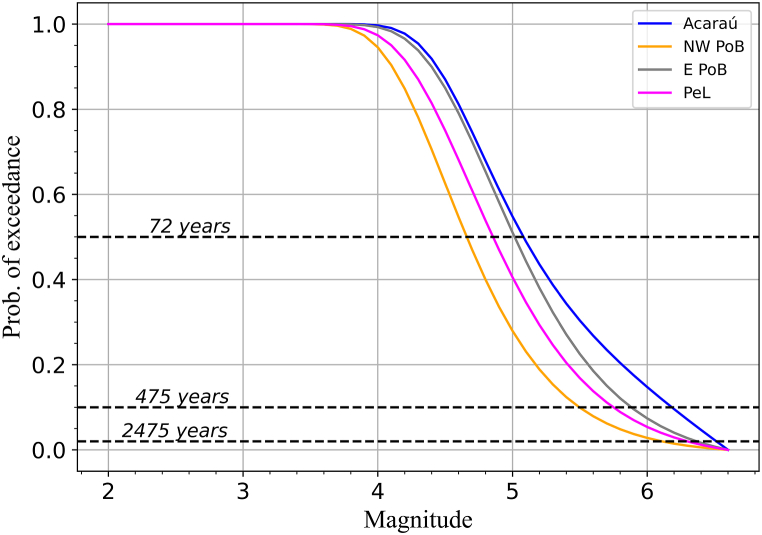
Exceedance probability of magnitude levels for each seismic zone in 50 years (color-coded as the legend indicates). Black dashed lines indicate 50 %, 10 %, and 2 % exceedance probabilities scenarios, corresponding to mean return periods of 72, 475, and 2475 years, respectively. (For interpretation of the references to colour in this figure legend, the reader is referred to the Web version of this article.)

Fig. 8 compares magnitude exceedance probabilities in the seismic zones in 50 years. For events of magnitudes up to 3.4 *m*_*R*_, the exceedance probability is very high, close to 100 %, and the differences among zones are insignificant. For larger events, the relation among the exceedance probabilities is Acaraú > E PoB > PeL > NW PoB. Horizontal black dashed lines mark the 50 %, 10 %, and 2 % exceedance probabilities, whose mean return periods correspond to 72, 475, and 2475 years, respectively. For the Acaraú, E PoB, PeL, and NW PoB seismic zones, there is a 50 % probability for the occurrence of earthquakes of magnitudes exceeding 5.1, 5.0, 4.9, and 4.7, respectively. The 10 % probability has the occurrence of earthquakes whose magnitudes exceed 6.2, 5.9, 5.8, and 5.5, respectively. For 2 % probability, the result is 6.5, 6.4, 6.3, and 6.1, respectively.

**Fig. 9:**
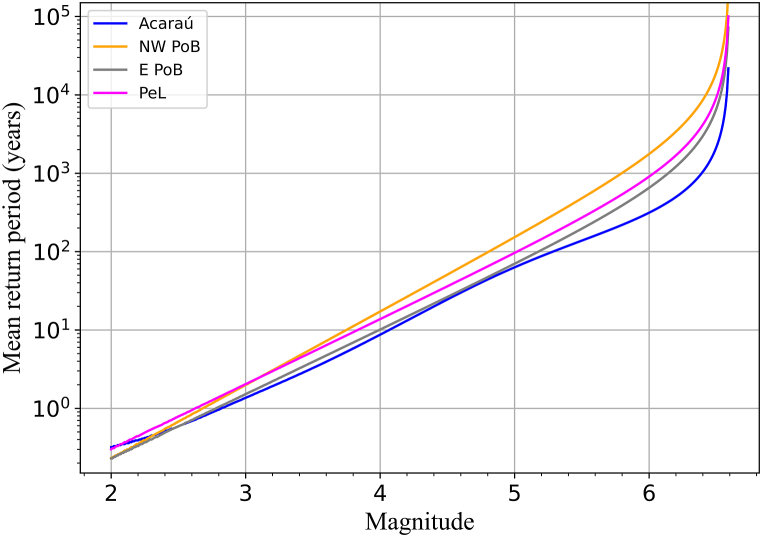
Magnitude mean return period for each seismic zone (color-coded as the legend indicates). (For interpretation of the references to colour in this figure legend, the reader is referred to the Web version of this article.)

Fig. 9 shows the mean return periods $T\left(M_{p}\right)$ of earthquakes as functions of magnitude, $M_{p}$. Up to $M_{p}=3.1$, the differences among the four seismic zones are minor (less than 1 year). When $M_{p}\geq 3.2$, $T\left(M_{p}\right)$ order is: Acaraú < E PoB < PeL < NW PoB. For example for an $M_{p}=4.5$ event, the mean return periods are 24.5, 26.4, 36, and 50.8 years, respectively, for a 5.5 event, $T\left(M_{p}\right)$ is 138, 196, 271 and 479 years, respectively, and for a 6.5 event, $T\left(M_{p}\right)$ is 2,134, 6,597, 9194 and 19,305 years, respectively.

We calculated maximum credible magnitudes for 50 and 70 years that are relevant for ordinary building infrastructures, and 10,000 years relevant for critical infrastructures. For 50 years, we obtained magnitudes of 4.9, 4.7, 4.7, and 4.5 for the Acaraú, E PoB, PeL, and NW PoB seismic zones, respectively; for 70 years, $T\left(M_{cred}\right)$ is 5.1, 5.0, 4.8, and 4.6, respectively; and for 10,000 years, $T\left(M_{cred}\right)$ is 6.6, 6.5, 6.5 and 6.4, respectively.

## 5 Discussion

We found the completeness magnitude estimates being the same in every temporally separated sub-catalogue except in the 1986–1989 sub-catalogue (Table 1). The greater Mc value for this sub-catalogue is probably due to two strong earthquakes followed by numerous aftershocks in 1986 and 1989. Such activity (JC sequence in Fig. 4a) artificially decreased the number of smaller events, possibly due to earthquake waveforms overlapping and/or some human factor in the analysis.

Reasenberg’s declustering algorithm identified 75 dependent events in the 1990–2020 dataset, indicating a data loss of approximately 9.34 % (Fig. 5b). Such a result suggests that seismicity declustering had a negligible impact on that dataset size. Still, the 1990–2020 dataset spans a very short time to represent long-term earthquake recurrence and, when split into the four seismic zones, provided too few events to estimate $M_{\max}$ from the catalogue data accurately. However, we believe that the upper 95 % confidence interval limit of event size estimated from palaeorecords provides a sufficiently conservative $M_{\max}$ estimate for the magnitude CDF modeling.

The b-values obtained from our MFD modelled by the G-R law range from 0.82 to 0.94, consistent with those reported in Ref. [80] (b-value = 0.8–0.9). It reinforces the reliability of our seismicity analysis. Compared to other area in South America SCR, i.e., the SE Brazil (with b-value typically varying from 1.02 to 1.30 [25,80,81]), our observed b-values fall within a lower range. This discrepancy reinforces that seismicity parameters across the South America SCR is not uniform, and can be attributed due to different condition in seismogenic processes as noted previously (e.g. Refs. [16,49]).

Contrary to other zones, the MFD in the Acaraú seismic zone was not exponential, i.e., it did not follow the G-R law. We calculated the upper bound of the 90 % confidence interval for the mean p-value to be 0.0194, reinforcing the robustness of our estimate. Even after considering potential uncertainty, the statistical significance of the mean p-value suggests that the data significantly deviates from the exponential distribution. One potential explanation for this deviation is that pore pressure diffusion is a driving mechanism for seismicity in the Acaraú seismic zone, as [32] pointed out. Pore pressure diffusion as a driving mechanism for seismicity yielding to MFD exponentiality deviation has been also reported before [82]. Another contributing factor to this deviation may be the greater variety of focal mechanisms within the Acaraú seismic zone [32] (and references therein). Unlike the other studied zones (NW PoB, E PoB, and PeL) characterized by uniform focal mechanisms, Acaraú exhibits a mix of strike-slip, normal, and reverse mechanisms. Simultaneously, the presence of varied focal mechanisms signifies complex stress interactions. This combined effect emphasizes the complex interplay that drives the seismic behaviour within the Acaraú seismic zone, resulting in a mixture of earthquake size distribution and yielding the observed deviation from the expected exponential pattern.

Fig. 8, Fig. 9 show that the zone's seismic hazard ranking is Acaraú > E PoB > PeL > NW PoB. Hence, the area where the largest earthquake in NE Brazil was recorded, NW PoB, poses the lowest seismic hazard, although it has the highest activity rate λ. Conversely, the area with the lowest λ, Acaraú, poses the highest seismic hazard. It is possible that the largest event in NE Brazil reduced the stress and the hazard levels in NW PoB. Furthermore, the strongest observed event does not strongly affect the long-term hazard. Therefore, while our findings may seem counterintuitive at first glance, they may highlight the interplay of geological factors in shaping the NE Brazil seismic hazard.

For the 50 % exceedance probability in 50 years, events range from 4.7 *m*_*R*_ to 5.1 *m*_*R*_ among the analysed seismic areas. Within the whole NE Brazilian catalogue, similar magnitude events (4.0 ≤ *m*_*R*_ ≤ 5.2) correspond to the maximum observed Modified Mercalli Intensities (MMI) [83], varying from V to VII. This correspondence is exemplified by the 5.1 mb João Câmara and 5.2 mb Cascavel events, which caused the collapse of poorly constructed buildings. Hence, our results suggest that potentially damaging earthquakes have a likely chance to occur in a relatively short period (72 years). However, compared to highly active intraplate areas, NE Brazil presents a low probability of larger magnitude exceedance probability. For instance, in the New Madrid seismic zone, the return period of magnitude 7 earthquakes is about 500 years [84], and the exceedance probability of such earthquakes in 50 years is about 10 %. According to our results, in NE Brazil, the 2 % exceedance probability in 50 years has earthquakes of magnitudes from 5.5 *m*_*R*_ to 6.2 *m*_*R*_.

In NE Brazil, palaeoearthquake average recurrence was estimated on the Jundiaí fault (Figs. 5a and b). It was 15.8 Ka for a 5.5 *Mw* (5.6 *m*_*R*_) event [9,73]. Our probabilistic analysis based on the observed seismicity for the same magnitude provided strongly different results. The mean return period in the four studied seismic zones varied from 160 to 609 years. However, our mean return period estimations are the order of the estimates from palaeoseismic records in other less active intraplate seismic areas. For magnitude 6, our mean return periods (313–1756 years) are comparable to the 350–1000 years average recurrence in the Saguenay region, E Canada [85]. Furthermore, such considerable variation of earthquake average recurrences, as in NE Brazil, was also obtained in E Canada. For the Charlevoix-Kamouraska seismic zone, which is approximately 70 km away from the Saguenay region, the mean return period of magnitude 6 events was estimated to be as short as 75, compared to 350–1000 years for the Saguenay region [85]. The opposite situation may have been in NE Brazil. The Jundiaí fault region, located approximately 55 km from the nearest to our seismic zones (E PoB), may have had much longer mean return periods than this zone. However, further investigations with denser populated seismic catalogues will help address this point better.

## 6 Conclusions

In one of the studied seismic zones exponentiality of MFD was rejected. We attributed this rejection to a combined effect of pore pressure diffusion and focal mechanism variability in the area.

Our analysis showed that in NE Brazil in 50 years, earthquakes of magnitudes from 4.7 to 5.1 have 50 % exceedance probabilities, and from 5.5 to 6.2 have 10 % exceedance probabilities. Such earthquakes can damage considerably poorly-built buildings in NE Brazil. Considering a period length of 10,000 years, suitable for critical infrastructures, maximum credible magnitudes are expected in the range of 6.4–6.6. Nevertheless, in NE Brazil, the seismic hazard is lower when compared to highly active intraplate areas, e.g., the New Madrid seismic zone.

The mean return periods calculated within our seismic zones are significantly shorter than the average recurrence based on palaeorecords on a near fault. The origin of the large difference between mean return periods obtained from our PSHA and palaeorecords in the Jundiaí fault is unclear. It can be genuine, but it can also be due to inaccurate return period estimations. A denser populated dataset would be necessary to better address this point.

We conducted the probabilistic analysis of the source component of seismic hazard. Based on the obtained results, we will assess the corresponding ground-shaking parameters in future work.

## Data availability

The earthquake catalogue used for our analysis (‘SISBRA - Catálogo de Sismos do Brasil’) was retrieved from https://seiscode.iag.usp.br/CSUSP/sisbra.

## CRediT authorship contribution statement

**J.A.S. Fonsêca:** Writing – review & editing, Writing – original draft, Visualization, Validation, Software, Methodology, Investigation, Formal analysis, Conceptualization. **S. Lasocki:** Writing – review & editing, Writing – original draft, Validation, Supervision, Software, Methodology, Investigation, Formal analysis, Conceptualization. **A.F. do Nascimento:** Writing – review & editing, Writing – original draft, Validation, Supervision, Investigation.

## Declaration of competing interest

The authors declare that they have no known competing financial interests or personal relationships that could have appeared to influence the work reported in this paper.
