# Supplementary material for: Probabilistic estimation of the source component of seismic hazard in North-Eastern Brazil
Source: Heliyon. 2024 May 3;10(9):e30716. doi: 10.1016/j.heliyon.2024.e30716 (PMC11098838; doi:10.1016/j.heliyon.2024.e30716)
Supplement: Multimedia component 1 [file mmc1.docx]

# **Supplementary material for**

**Probabilistic estimation of the source component of seismic hazard in North-Eastern Brazil.**

J. A. S. Fonsêca (augusto.fonseca.108@ufrn.edu.br)^a^; Stanisław Lasocki (lasocki@igf.edu.pl) ^b^; A. F. do Nascimento (aderson.nascimento@ufrn.br) ^1^

^a^ Departamento de Geofísica, Universidade Federal do Rio Grande do Norte, Campus Universitário, Natal, RN 59078-970, Brazil

^b^ Institute of Geophysics Polish Academy of Sciences, Department of Seismology, Krakow, Poland

**The present Supplementary Material (SM) file contains:**

Supporting text

Figures S1 to S9

Table S1

SM References

**Mc estimation**

Many methods have been proposed to assess Mc, as [1] summarize. In this section, we describe the methods and results of Mc estimations obtained by MAXC [2], GFT [2], and MGFT [3] approaches for each one of the 5 divided by period sub-catalogues: 1) from 1980 until 2009; 2) from 1980 until 1985; 3) from 1986 until 1989; 4) from 1990 until 2009; 5) from 2010 onwards.

The MAXC method [2] bases the estimation of Mc by defining the magnitude corresponding to the highest number of events in the non-cumulative FMD (maximum curvature). One of the advantages one can take is that this approach works well with small datasets, obtaining stable results when they do not contain strong heterogeneities. However, Mc estimation can usually be underestimated.

A more conservative method, GFT [2], evaluates the difference between the observed FMD and a synthetic distribution as a function of several Mc values (Mc_i_). First, for each observed Mc_i_, b- and a-values of the Gutenberg-Richter (G-R) relationship are estimated using the maximum likelihood approach for events with Magnitudes ≥ Mc_i_. Then, synthetic distributions perfectly fitted to GR power law are constructed based on the previously calculated GR parameters. Finally, goodness of fit is calculated using the absolute difference of the number of events in each magnitude bin between the observed and the synthetic distributions as follows below:

$$R\left( a,b,M_{\dot{i}} \right)=100-\left( \frac{\sum_{{Mc}_{i}}^{Mmax} \left| B_{i}-S_{i} \right|\cdot100}{\sum_{i} B_{i}} \right)$$

(1),

where $B_{i}$ and $S_{i}$ are the observed and predicted cumulative number of events in each magnitude bin. Thus, Mc is estimated where the residual $R\left( a,b,M_{\dot{i}} \right)$ first reaches a predefined confidence level in a residual-magnitude plot. In this work, we chose 90 % as the confidence level at which modelled data can explain variability.

A modification of the GFT method (MGFT) was proposed by [3]. Many catalogues (*k*) are synthesized with random values following the G-R law obtained from the observed data. Each synthetic catalogue contains N_i_ events, the cumulative number of events with M ≥ Mc_i_. Then, a- and b-values are calculated by maximum-likelihood estimation for each synthetic dataset considering the entire range Mc_i_ ≥ M ≤M_max_. Thus, for each magnitude bin, there are k synthetic values whose difference to the observed data is calculated using equation 1. Here, we used *k* = 1,000. Such differences are averaged so that for each magnitude bin, there is one mean value of these differences. Repeating the same procedure as a function of Mc_i_, the Mc estimation relies on the value corresponding to the minimum point, regardless of any specific confidence level.

The results were obtained by using the *Completeness Magnitude estimation* application on the EPISODES platform (https://episodesplatform.eu; [4]) and are presented in Figs S1, S2, S3, S4 and S5, respectively. Each Figure illustrates the Number of events as a function of magnitude mR in its upper panel, in which the cumulative and non-cumulative distributions are symbolized by black squares and gray circles, respectively. The vertical blue line indicates the Mc estimate of MAXC. In its lower panel is presented the residual plot against magnitude m_R_. The red squares and black circles symbolize the differences between observed and synthetic data for MGFT (average difference) and GFT methods, respectively. The horizontal dashed green line represents the residual level of 10%, at which modelled data can explain 90% of data variability.

In the 1980-2009 sub-catalogue, there were 660 events. For this dataset, we can observe in Fig. S1 (lower panel) that GFT distribution first reaches 10% in the magnitude bin = 3.0 on the residual plot. On the other hand, although MGFT tends to follow GFT, a more prominent minimum local is found on smaller magnitudes (lower panel). This value coincides with the magnitude bin, corresponding to the highest number of events in the non-cumulative FMD (Mc estimation by MAXC in the upper panel). As a result, MAXC and MGFT converged to the same result (Mc = 2.0), while the GFT result was significantly different (Mc = 3.0).

For the 1980-1985 sub-catalogue (Fig. S2), GFT distribution (lower panel) points to a more prominent minimum residual when compared to the 1980-2009 sub-catalogue results. However, this is observed on smaller magnitudes. Still, it is most highlightable by the distribution of MGFT (lower panel), coinciding again with the estimation of Mc by MAXC (upper panel). Thus, all methods converged to the same Mc = 2.0. However, although all estimations of Mc matched, it must be noticed that only 49 events are present in this sub-catalogue, contributing to a poorer-shaped non-cumulative FMD (upper panel). Consequently, uncertainties can ben be significant.

For the 145 events in the 1986-1989 sub-catalogue (Fig. S3), the same pattern can be observed for the dataset's period between 1980 and 2009 can be observed. The minimum value pointed by MGFT (lower panel) coincides with the maximum curvature pointed by MAXC (upper panel). In comparison, a residual level of 10% is achieved on a larger magnitude by GFT (lower panel). However, in this comparison, all Mc estimations are higher (for MGFT and MAXC, Mc=3.0; for GFT, Mc=3.6).

The 1990-2009 and 2010-2020 sub-categories present different FMDs (Fig. S4 with 466 events and Fig. S5 with 578 occurrences, respectively). Despite that, Mc estimations follow the same pattern found in the 1980-1985 sub-catalogue, hence the convergence of all methods to Mc = 2.0.


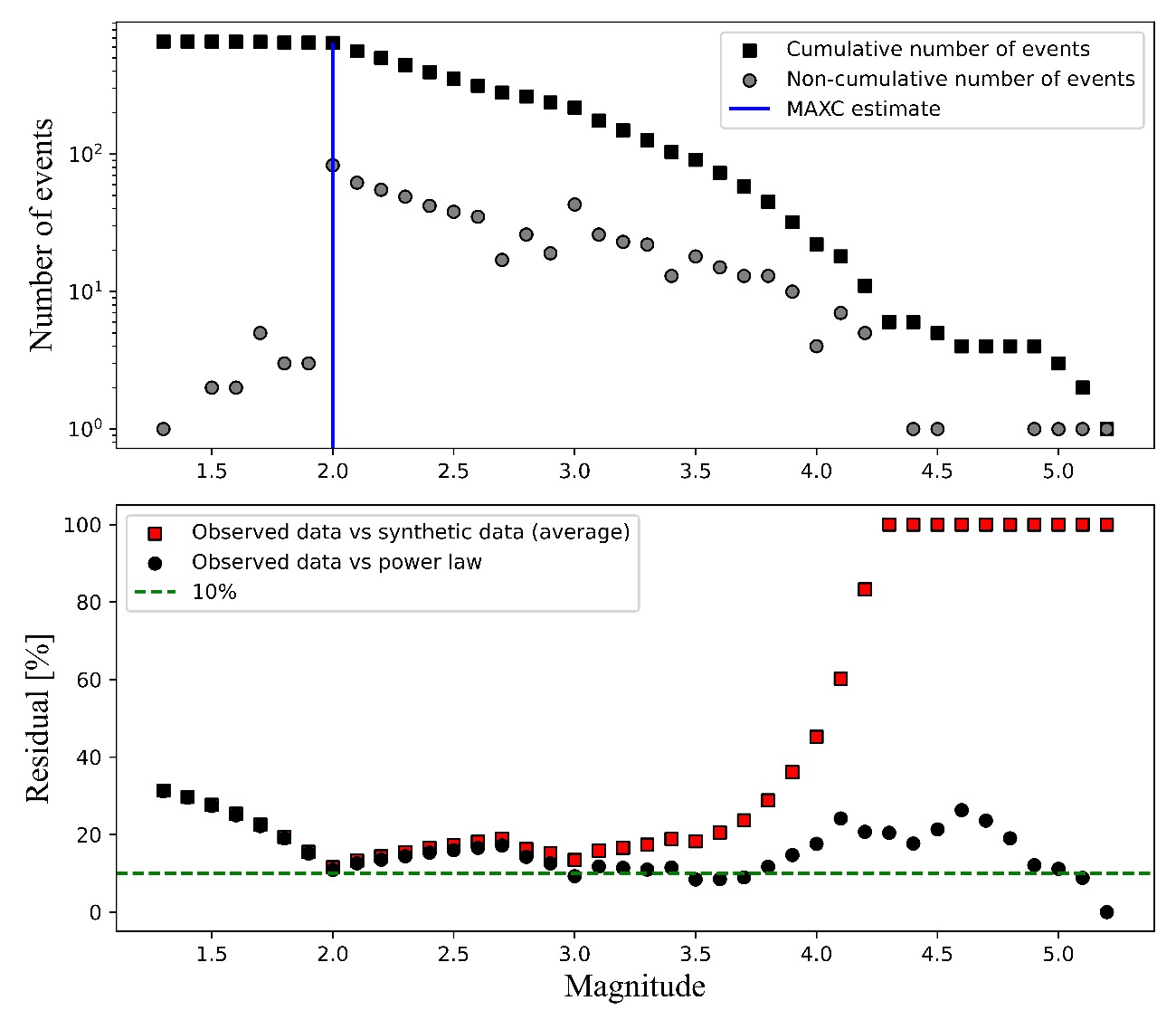


*Figure S1: Mc estimation for the 1980-2009 sub-catalogue by applying MAXC, GFT, and MGFT methods. Upper panel: Number of events with respect to Magnitude m_R_. Cumulative and non-cumulative distributions are represented by the black squares and gray circles, respectively, while the vertical blue line indicates the MAXC estimate. Lower panel: Residual plot against magnitude m_R_. Red squares are the average values of differences between observed and synthetic data for MGFT, while black circles symbolize the difference between observed and synthetic data for GFT. The green dashed line indicates the residual level of 10%.*


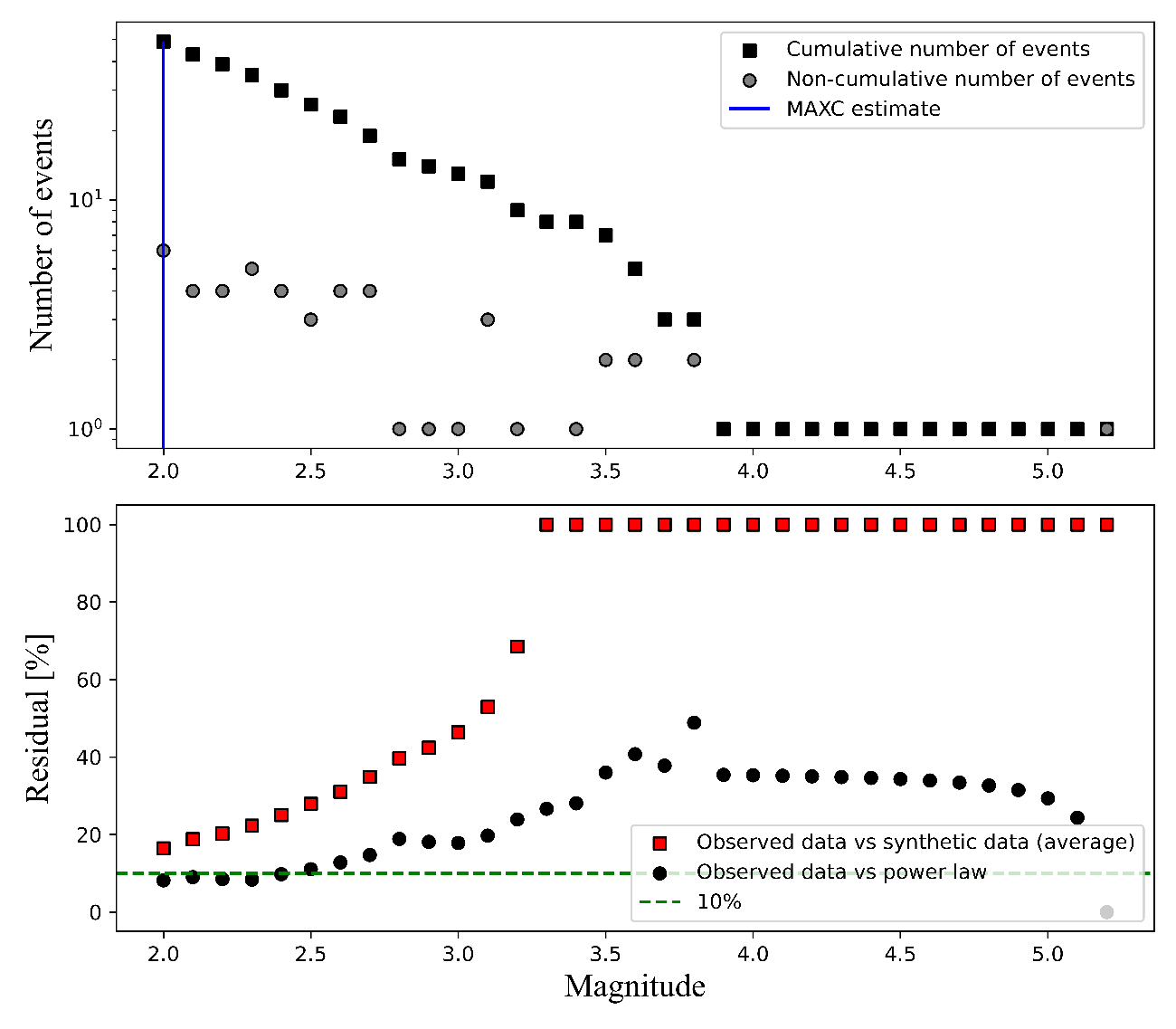


*Figure S2: Mc estimation for the 1980-1985 sub-catalogue by applying MAXC, GFT, and MGFT methods similarly to Fig. S1.*


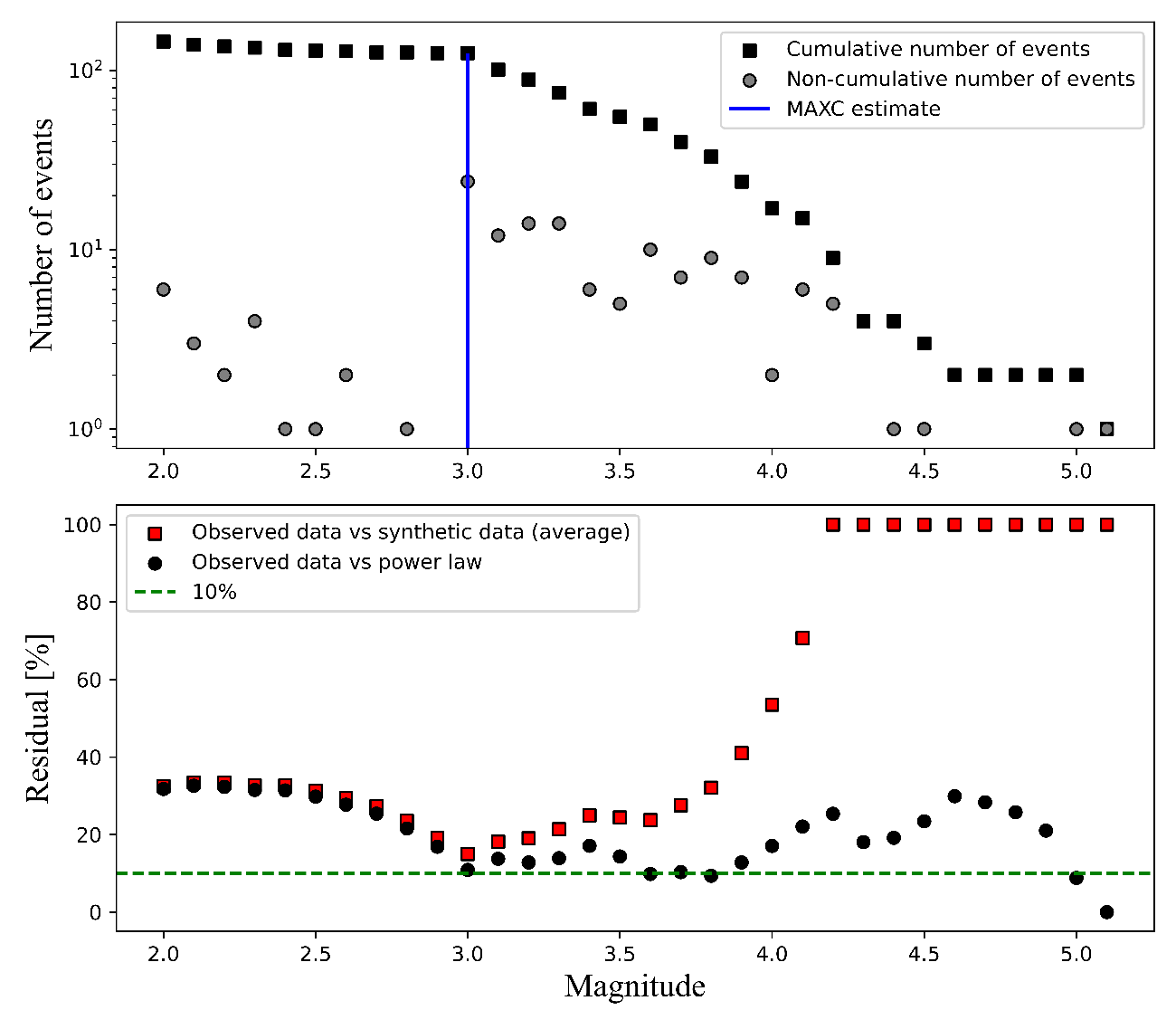


*Figure S3: Mc estimation for the 1986-1989 sub-catalogue by applying MAXC, GFT, and MGFT methods similarly to Fig. S1.*


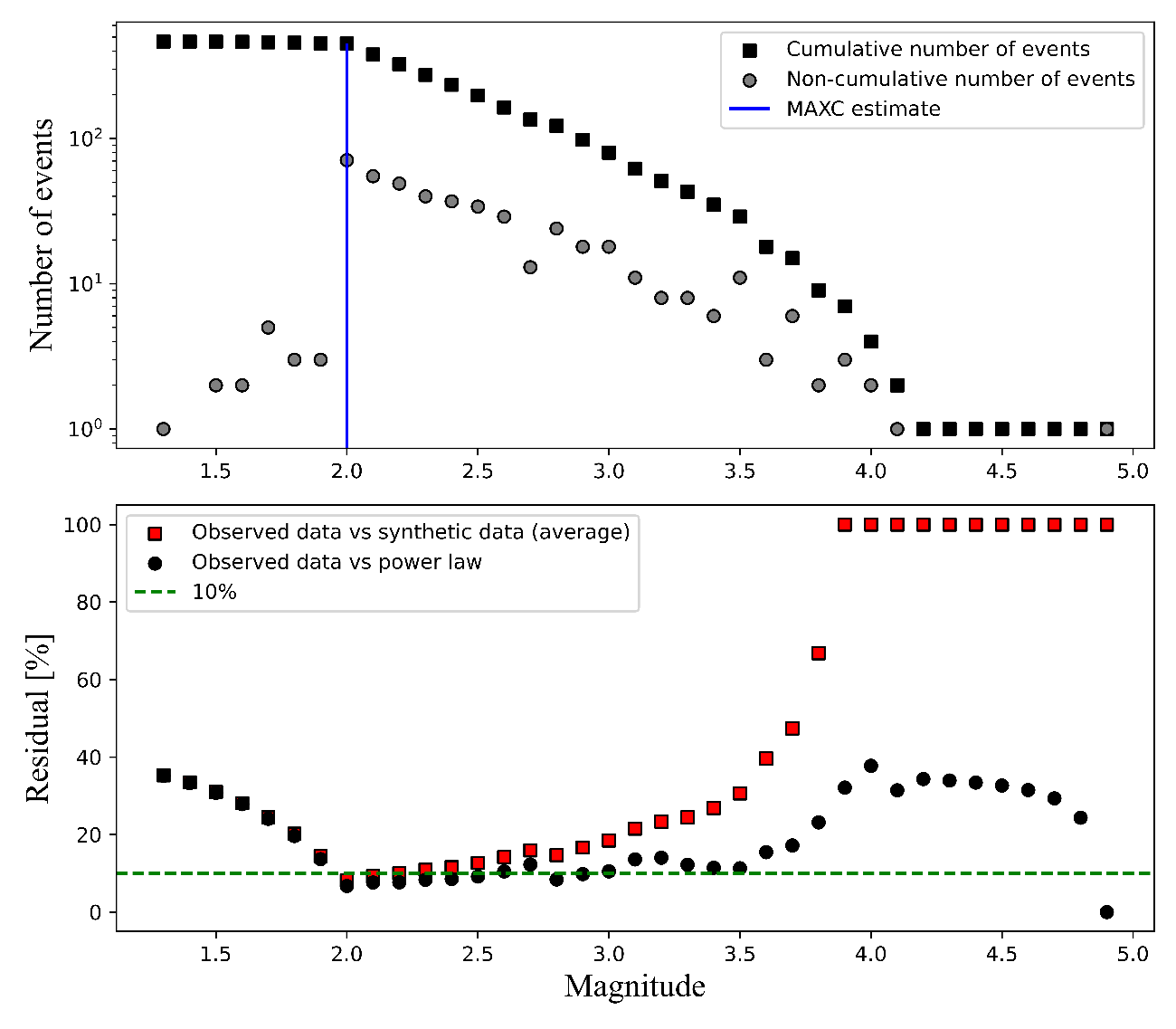


*Figure S4: Mc estimation for the 1990-2009 sub-catalogue by applying MAXC, GFT, and MGFT methods similarly to Fig. S1.*


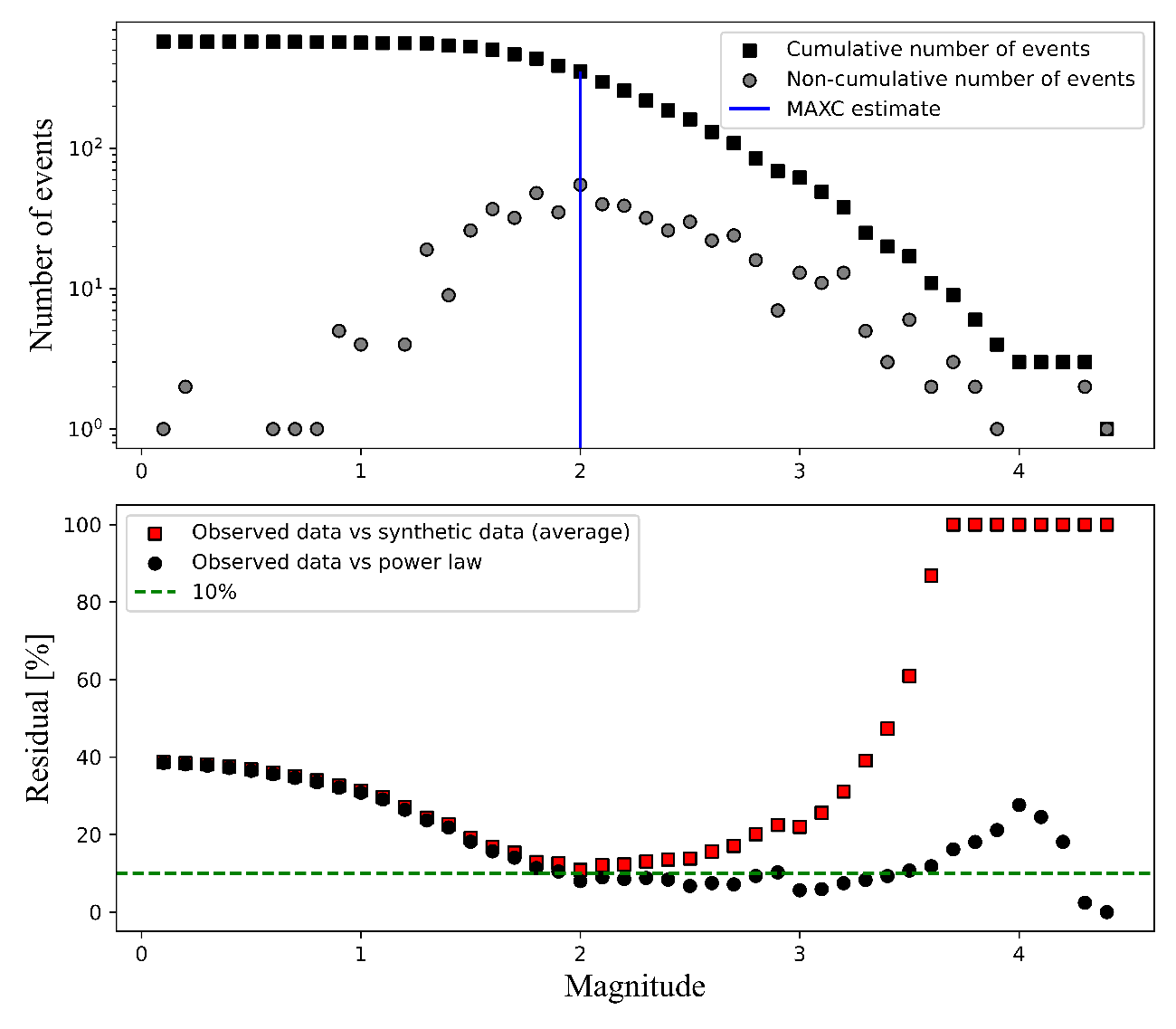


*Figure S5: Mc estimation for the 2010-2020 sub-catalogue by using MAXC, GFT, and MGFT methods similarly to Fig. S1.*

Despite the differences between Mc estimations, MGFT and MAXC provided the same solutions in every tested sub-catalogue. Although containing higher residuals, MGFT is less sensitive to specific confidence levels and evaluates a more objective criterion [3]. Because of this, the selection of the proper value of Mc for each sub-catalogue was based on the convergence of MGFT and MAXC results when they departed from MAXC.

**Anderson-Darling test**

We used the Anderson-Darling (A-D) test [5] for verifying if our set of magnitude distributions can be explained by the G-R magnitude distribution model. Specifically, A-D test performs a goodness of fit test between the exponential and the magnitude distribution under a specific level of confidence α. We used the *Anderson-Darling test for magnitude distribution* application on the EPISODES platform (https://episodesplatform.eu; [4]) for applying the A-D test, considering α = 5 %, following similar procedure as [6]. The p-values obtained were the average between 100 trials of the A-D test. Figs S6, S7, S8, and S9 show the result of A-D test applied to Acaraú, NW PoB, E PoB, and PeL seismic zones, respectively.


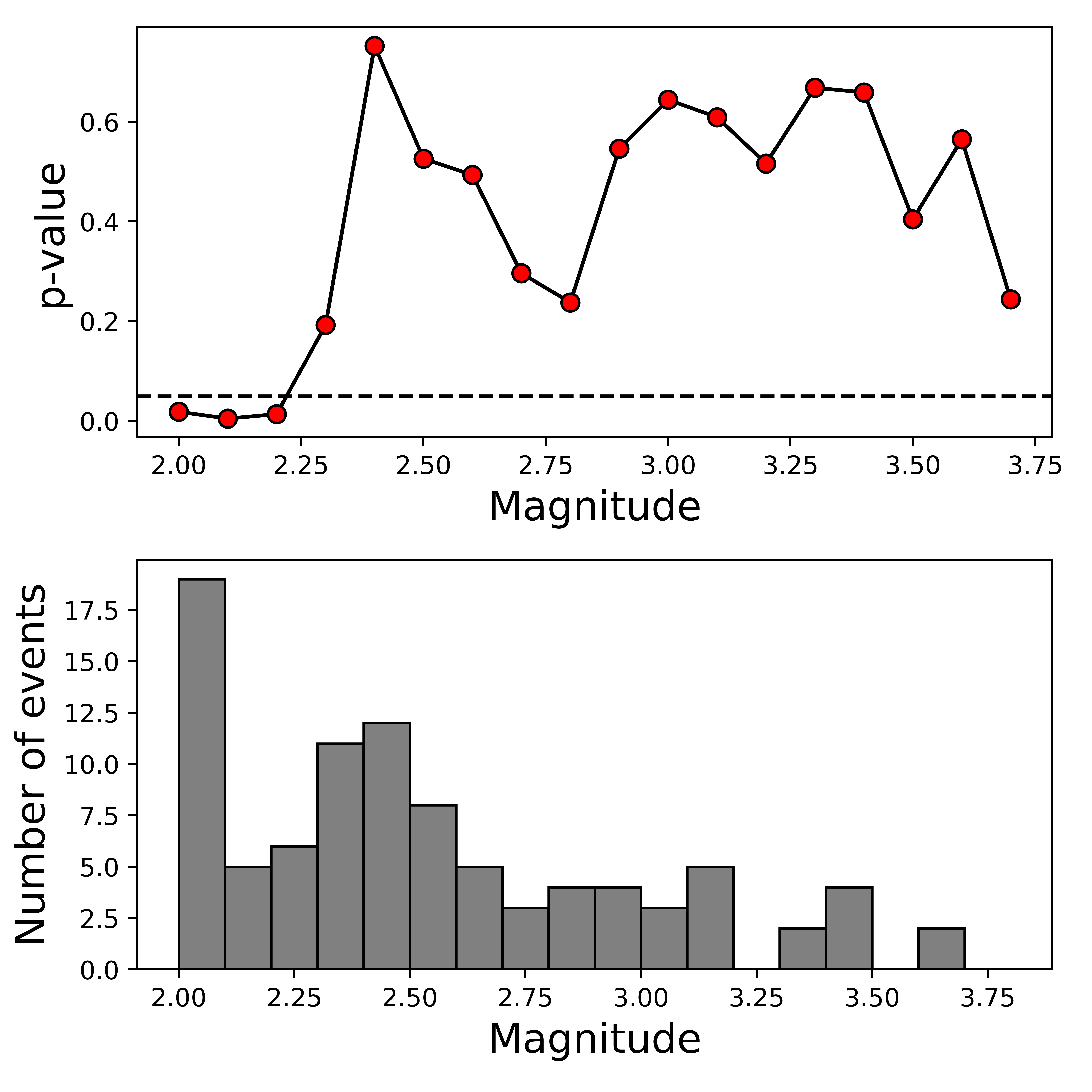


Figure S6: A-D test performance of Acaraú magnitude distribution. Upper pannel: mean p-value against magnitude. The dashed black line represents α. Lower pannel: Number of events against magnitude.


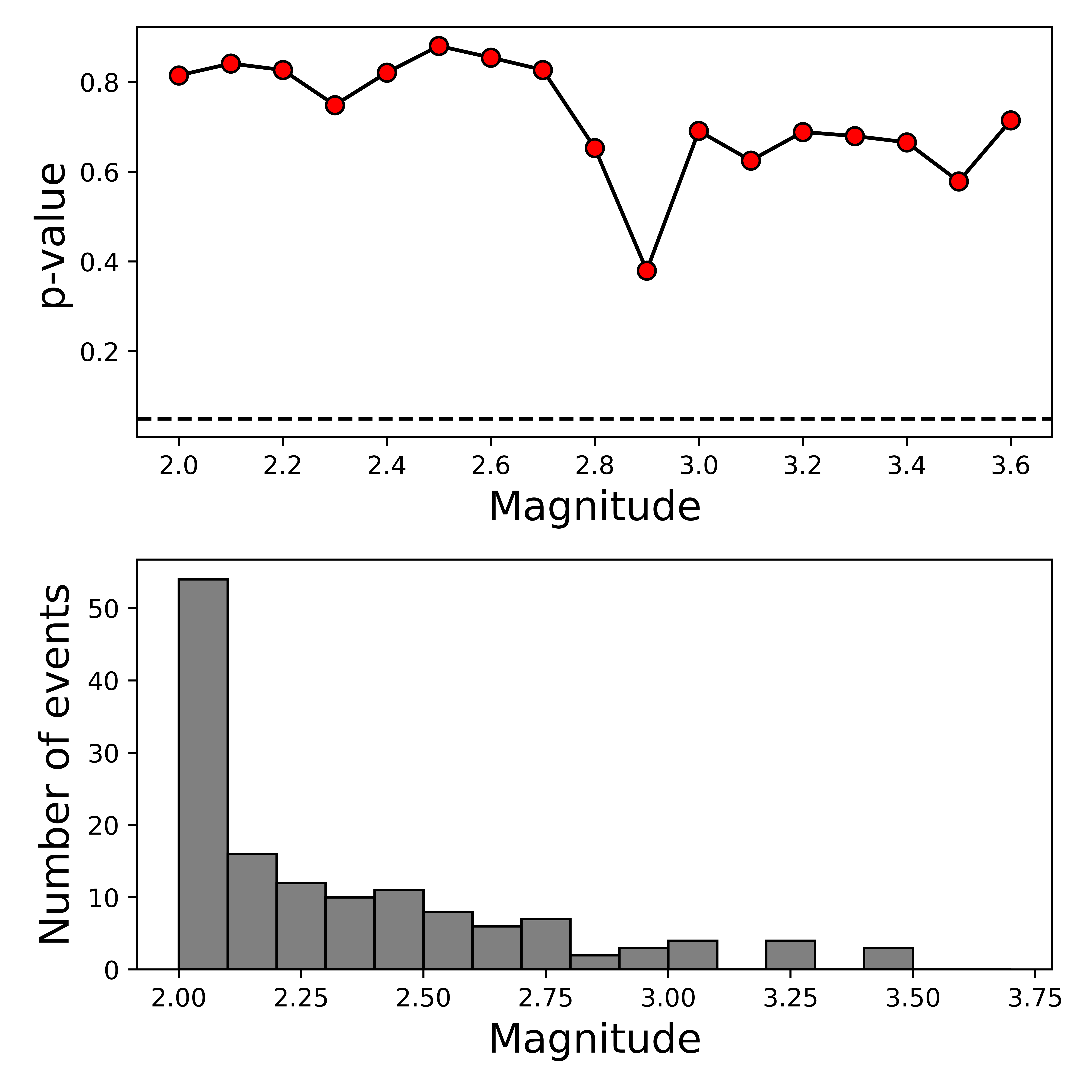


Figure S7: A-D test performance of NW PoB magnitude distribution (same as Fig. S6).

**
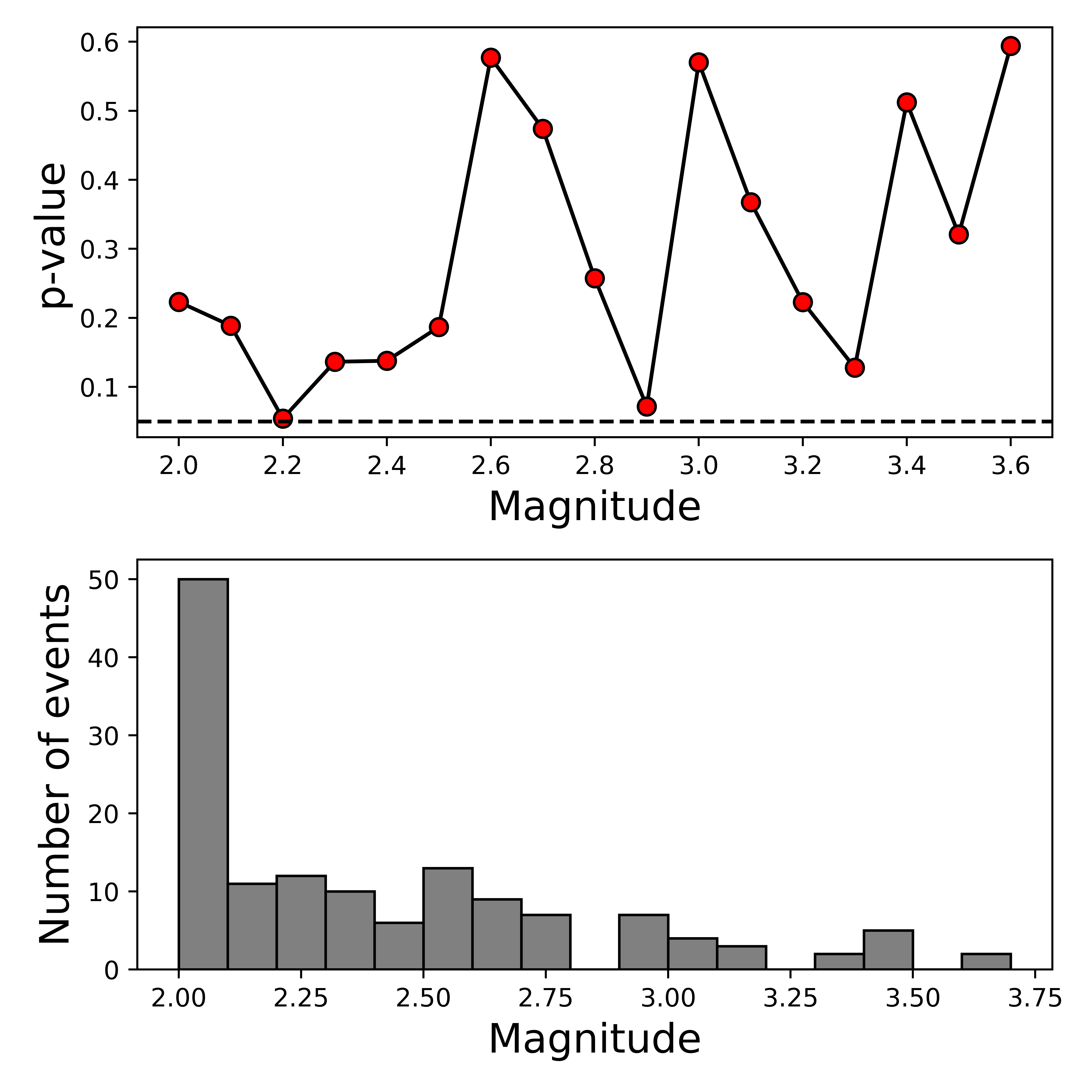
**

Figure S8: A-D test performance of E PoB magnitude distribution (same as Fig. S6).


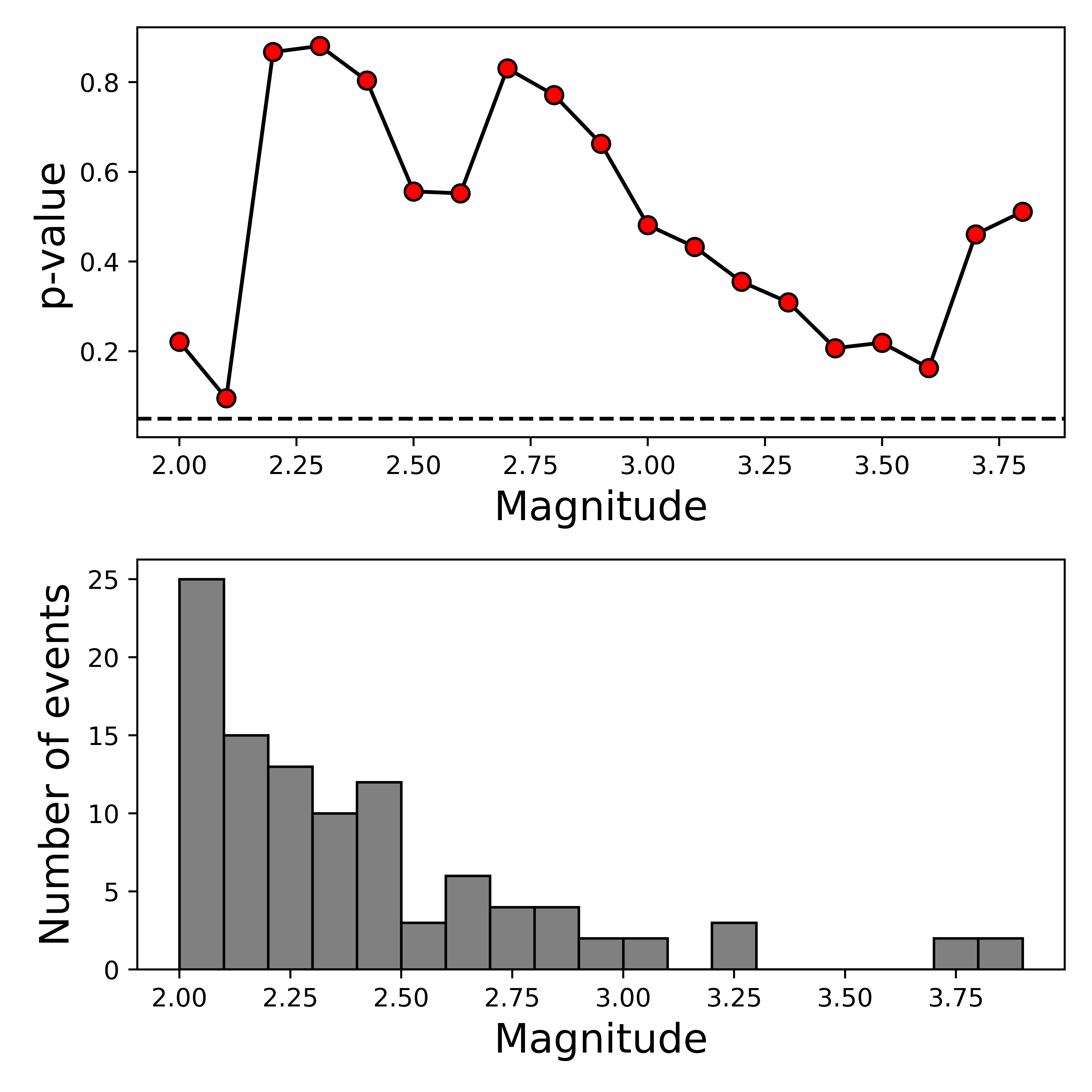


Figure S9: A-D test performance of PeL magnitude distribution (same as Fig. S6).

The mean p-value for the Acaraú seismic zone was significantly below α for magnitude bin = Mc (Mc = 2.0). It suggests evidence for magnitude distribution exponentiality rejection. The mean p-value for NW PoB, E PoB, and PeL seismic zones were significantly larger than α, suggesting evidence for non-rejection of exponentialy for their magnitude distributions.

**Earthquake Catalogue**

In this section, we present the Brazilian Seismic Catalog (Catálogo Sísmico Brasileiro – SISBRA; version “catalogo_ate2020_CLEAN_v2022Jan27” retrieved from <https://seiscode.iag.usp.br/CSUSP/sisbra>) resulted from the removal of events with location error greater than 50 km and whose month and day were not provided. It is the initial data set for our analysis and is presented in Table S1. Table S1 shows parameter (column header) as follows: year (year), month (mm), day (dd), hour (hh), second (ss.s), latitude in degree (latit), longitude in degree (longit), depth in km (depth), epicentral error in km (err), magnitude (mag), maximum modified Mercalli intensity. The value of 0 in epicntral uncertainty means lack of information.

Table S1: Filtered SISBRA earthquake catalogue.

| **year** | **mm** | **dd** | **hh** | **min** | **ss.s** | **latit** | **longit** | **depth** | **err** | **mag** | **Io** |
| --- | --- | --- | --- | --- | --- | --- | --- | --- | --- | --- | --- |
| 1724 | 1 | 4 | 7 | 30 | 0 | -13 | -38.5 | 0 | 0 | 2.8 | 3-4 |
| 1811 | 10 | 28 | 19 | 30 | 0 | -8.1 | -34.9 | 0 | 50 | 3.8 | 5 |
| 1852 | 12 | 2 | 13 | 30 | 0 | -4.6 | -37.8 | 0 | 0 | 3 | 4 |
| 1854 | 1 | 10 | 7 | 0 | 0 | -5.2 | -35.5 | 0 | 50 | 3.7 | 5-6 |
| 1864 | 11 | 26 | 1 | 30 | 0 | -2.5 | -44.3 | 0 | 0 | 3.2 | 4-5 |
| 1865 | 7 | 5 | 18 | 0 | 0 | -4.4 | -39.3 | 0 | 0 | 3.8 | 4 |
| 1871 | 4 | 5 | 1 | 30 | 0 | -3.4 | -44.4 | 0 | 0 | 3.7 | 5-6 |
| 1879 | 7 | 24 | 17 | 25 | 0 | -6 | -35.5 | 0 | 50 | 4.3 | 5 |
| 1882 | 5 | 1 | 23 | 0 | 0 | -3.4 | -44.4 | 0 | 0 | 3.2 | 4 |
| 1891 | 12 | 21 | 9 | 30 | 0 | -13 | -39.1 | 0 | 0 | 3 | 4 |
| 1901 | 5 | 25 | 23 | 20 | 0 | -9.5 | -36.1 | 0 | 0 | 3.2 | 4-5 |
| 1901 | 5 | 26 | 2 | 15 | 0 | -9.5 | -36 | 0 | 20 | 3.6 | 5-6 |
| 1903 | 2 | 10 | 0 | 0 | 0 | -4.4 | -39 | 0 | 30 | 3.9 |  |
| 1903 | 2 | 12 | 0 | 0 | 0 | -4.4 | -39 | 0 | 30 | 3.9 |  |
| 1903 | 2 | 14 | 0 | 0 | 0 | -4.4 | -39 | 0 | 30 | 4.1 | 6 |
| 1903 | 2 | 15 | 0 | 0 | 0 | -4.4 | -39 | 0 | 30 | 4.1 | 6 |
| 1903 | 2 | 16 | 0 | 0 | 0 | -4.4 | -39 | 0 | 30 | 4.1 | 6 |
| 1903 | 5 | 14 | 13 | 0 | 0 | -4.1 | -38.8 | 0 | 0 | 3.6 |  |
| 1909 | 12 | 23 | 19 | 0 | 0 | -2.4 | -44.4 | 0 | 0 | 3 | 4 |
| 1911 | 3 | 15 | 20 | 55 | 0 | -13 | -38.7 | 0 | 20 | 3 |  |
| 1911 | 3 | 22 | 15 | 0 | 0 | -13 | -38.7 | 0 | 20 | 4 | 7 |
| 1911 | 8 | 26 | 21 | 15 | 0 | -3.1 | -40.8 | 0 | 0 | 3.4 |  |
| 1912 | 4 | 19 | 5 | 0 | 0 | -13 | -38.9 | 0 | 0 | 3.7 | 5-6 |
| 1915 | 11 | 6 | 15 | 30 | 0 | -13 | -38.6 | 0 | 20 | 4 | 6 |
| 1917 | 11 | 7 | 20 | 25 | 0 | -12 | -38.7 | 0 | 10 | 4.3 | 6-7 |
| 1917 | 11 | 7 | 22 | 0 | 0 | -12 | -38.6 | 0 | 0 | 3.7 | 4 |
| 1918 | 1 | 12 | 8 | 0 | 0 | -12 | -38.6 | 0 | 0 | 3.7 | 5-6 |
| 1919 | 11 | 9 | 11 | 0 | 0 | -13 | -38.7 | 0 | 20 | 3.8 | 4 |
| 1919 | 11 | 10 | 0 | 0 | 0 | -13 | -38.6 | 0 | 20 | 3.6 | 4 |
| 1919 | 11 | 13 | 0 | 0 | 0 | -13 | -38.7 | 0 | 0 | 3.5 | 5 |
| 1919 | 11 | 16 | 6 | 55 | 0 | -13 | -38.7 | 0 | 20 | 3.5 | 5 |
| 1919 | 11 | 23 | 1 | 20 | 0 | -13 | -38.6 | 0 | 10 | 4.2 | 7 |
| 1919 | 11 | 24 | 2 | 50 | 0 | -3.9 | -38.9 | 0 | 50 | 4.6 | 5 |
| 1920 | 2 | 16 | 22 | 30 | 0 | -4.4 | -39.7 | 0 | 0 | 3.8 |  |
| 1923 | 10 | 1 | 11 | 20 | 0 | -8.5 | -36.7 | 0 | 20 | 4.1 | 5 |
| 1928 | 4 | 14 | 21 | 59 | 0 | -4.6 | -37.8 | 0 | 0 | 4 | 6 |
| 1928 | 6 | 7 | 21 | 30 | 0 | -5.4 | -39.4 | 0 | 0 | 3.6 | 3-4 |
| 1929 | 12 | 20 | 22 | 40 | 0 | -8.3 | -36 | 0 | 0 | 3 |  |
| 1947 | 3 | 4 | 0 | 0 | 0 | -2.5 | -44.4 | 0 | 10 | 3 | 4 |
| 1949 | 7 | 12 | 0 | 0 | 0 | -8.2 | -35.1 | 0 | 0 | 3.3 | 4 |
| 1950 | 4 | 7 | 12 | 0 | 0 | -5.5 | -35.8 | 0 | 0 | 3 | 4 |
| 1961 | 6 | 11 | 2 | 0 | 0 | -9.6 | -36 | 0 | 0 | 2 | 4 |
| 1963 | 8 | 27 | 0 | 0 | 0 | -5.7 | -36.2 | 0 | 0 | 3.7 | 5-6 |
| 1963 | 10 | 2 | 15 | 40 | 0 | -5.7 | -36.2 | 0 | 0 | 3.9 | 6 |
| 1964 | 1 | 19 | 0 | 0 | 0 | -8.3 | -36 | 0 | 0 | 3.5 | 5 |
| 1964 | 6 | 16 | 0 | 0 | 0 | -8.3 | -36 | 0 | 0 | 3.5 | 5 |
| 1967 | 1 | 21 | 15 | 59 | 48 | -8.2 | -36 | 0 | 10 | 3.8 | 5 |
| 1968 | 1 | 13 | 1 | 55 | 51 | -6.1 | -38.4 | 0 | 10 | 3.9 | 6 |
| 1968 | 1 | 13 | 1 | 58 | 0 | -6 | -38.5 | 0 | 0 | 2.8 | 3-4 |
| 1968 | 1 | 18 | 10 | 40 | 0 | -6.1 | -38.4 | 0 | 0 | 3.5 | 5 |
| 1968 | 2 | 4 | 0 | 0 | 0 | -6.1 | -38.4 | 0 | 0 | 3.2 | 4-5 |
| 1968 | 2 | 15 | 13 | 20 | 48 | -6.1 | -38.4 | 0 | 10 | 4.1 | 6 |
| 1968 | 2 | 23 | 14 | 23 | 2 | -6.1 | -38.4 | 0 | 5 | 4.6 | 7 |
| 1968 | 2 | 23 | 14 | 37 | 1 | -6.1 | -38.4 | 0 | 10 | 3 |  |
| 1968 | 2 | 23 | 15 | 21 | 16 | -6.1 | -38.4 | 0 | 10 | 3 |  |
| 1968 | 2 | 23 | 15 | 33 | 3 | -6.1 | -38.4 | 0 | 10 | 3.8 |  |
| 1968 | 3 | 18 | 0 | 0 | 0 | -6 | -38.5 | 0 | 0 | 3.7 | 5-6 |
| 1970 | 1 | 29 | 0 | 0 | 0 | -8 | -36.2 | 0 | 0 | 3.9 | 6 |
| 1970 | 1 | 30 | 0 | 0 | 0 | -8 | -36.2 | 0 | 0 | 3.5 | 5 |
| 1971 | 8 | 5 | 2 | 30 | 0 | -8 | -34.9 | 0 | 0 | 3 | 5 |
| 1972 | 3 | 4 | 21 | 45 | 0 | -9.9 | -36.5 | 0 | 0 | 3.1 | 5 |
| 1972 | 6 | 4 | 4 | 30 | 0 | -6.2 | -38.5 | 0 | 0 | 3 | 4 |
| 1973 | 4 | 26 | 14 | 15 | 2 | -5.3 | -35.8 | 0 | 0 | 3.6 |  |
| 1973 | 4 | 26 | 18 | 54 | 57 | -5.3 | -35.8 | 0 | 0 | 3.6 |  |
| 1973 | 4 | 27 | 8 | 20 | 13 | -5.3 | -35.8 | 0 | 0 | 3 |  |
| 1973 | 5 | 2 | 3 | 41 | 14 | -5.3 | -35.8 | 0 | 0 | 3 |  |
| 1973 | 5 | 2 | 3 | 47 | 58 | -5.3 | -35.8 | 0 | 0 | 3.1 |  |
| 1973 | 5 | 2 | 17 | 34 | 34 | -5.3 | -35.8 | 0 | 0 | 2.3 | 4 |
| 1973 | 6 | 17 | 4 | 22 | 29 | -5.3 | -35.8 | 0 | 0 | 3 |  |
| 1973 | 6 | 26 | 5 | 25 | 2 | -5.3 | -35.8 | 0 | 0 | 3.1 |  |
| 1973 | 7 | 20 | 13 | 35 | 25 | -5.3 | -35.8 | 0 | 0 | 3.9 |  |
| 1973 | 7 | 22 | 21 | 22 | 50 | -5.3 | -35.8 | 0 | 10 | 4.3 | 6-7 |
| 1973 | 7 | 22 | 21 | 33 | 27 | -5.3 | -35.8 | 0 | 0 | 3 |  |
| 1973 | 7 | 23 | 13 | 27 | 10 | -5.3 | -35.8 | 0 | 0 | 3 |  |
| 1973 | 7 | 23 | 23 | 35 | 51 | -5.3 | -35.8 | 0 | 0 | 3 |  |
| 1974 | 10 | 20 | 21 | 37 | 32 | -8 | -36.1 | 0 | 10 | 3.6 | 5 |
| 1974 | 10 | 20 | 21 | 59 | 0 | -8 | -36.1 | 0 | 0 | 2.9 |  |
| 1974 | 10 | 20 | 23 | 2 | 42 | -8 | -36.1 | 0 | 0 | 2.7 |  |
| 1974 | 10 | 21 | 2 | 18 | 11 | -8 | -36.1 | 0 | 0 | 2.8 |  |
| 1974 | 10 | 21 | 2 | 29 | 41 | -8 | -36.1 | 0 | 0 | 2.7 |  |
| 1974 | 12 | 15 | 5 | 14 | 46 | -3.7 | -39.2 | 0 | 0 | 3.3 | 6 |
| 1976 | 5 | 18 | 8 | 30 | 0 | -15 | -39.6 | 0 | 20 | 3.7 | 6 |
| 1976 | 5 | 25 | 8 | 30 | 0 | -15 | -39.6 | 0 | 20 | 3.9 | 6 |
| 1976 | 6 | 8 | 21 | 0 | 0 | -5.7 | -35.5 | 0 | 0 | 3.4 |  |
| 1976 | 7 | 12 | 14 | 0 | 0 | -15 | -40.3 | 0 | 0 | 3.2 | 4-5 |
| 1976 | 7 | 27 | 21 | 0 | 0 | -4.8 | -38.8 | 0 | 0 | 3 | 4 |
| 1976 | 7 | 29 | 18 | 0 | 0 | -4.8 | -38.8 | 0 | 0 | 3.5 | 5 |
| 1976 | 8 | 11 | 20 | 0 | 0 | -15 | -39.6 | 0 | 0 | 3.5 | 5 |
| 1977 | 2 | 25 | 13 | 40 | 0 | -5.7 | -35.8 | 0 | 10 | 3.6 | 6-7 |
| 1977 | 3 | 12 | 7 | 0 | 0 | -4.8 | -38.8 | 0 | 0 | 3.9 | 6 |
| 1978 | 2 | 10 | 0 | 0 | 0 | -6.3 | -36 | 0 | 15 | 3.7 | 5 |
| 1979 | 5 | 29 | 20 | 58 | 3 | -9.7 | -37.5 | 0 | 50 | 2.4 |  |
| 1980 | 11 | 20 | 3 | 29 | 45 | -4.3 | -38.4 | 5 | 5 | 5.2 | 7 |
| 1980 | 11 | 20 | 5 | 27 | 11 | -4.3 | -38.4 | 0 | 30 | 2.2 |  |
| 1980 | 11 | 21 | 21 | 28 | 44 | -4.3 | -38.4 | 0 | 30 | 2.4 |  |
| 1980 | 12 | 24 | 13 | 35 | 0 | -4.3 | -38.4 | 0 | 0 | 2.3 |  |
| 1981 | 1 | 2 | 4 | 14 | 55 | -4.3 | -38.4 | 0 | 30 | 3.6 |  |
| 1981 | 1 | 6 | 19 | 51 | 58 | -5 | -47.5 | 0 | 50 | 3.4 |  |
| 1981 | 1 | 12 | 3 | 33 | 8 | -4.3 | -38.4 | 0 | 30 | 3.8 |  |
| 1981 | 1 | 12 | 9 | 29 | 0 | -4.3 | -38.4 | 0 | 0 | 2 |  |
| 1981 | 4 | 21 | 18 | 53 | 0 | -4.8 | -38.6 | 0 | 50 | 2.5 |  |
| 1981 | 4 | 22 | 1 | 5 | 0 | -4.3 | -38.4 | 0 | 0 | 2.1 |  |
| 1981 | 9 | 9 | 23 | 12 | 0 | -4.3 | -38.4 | 0 | 0 | 2.6 |  |
| 1981 | 9 | 10 | 15 | 56 | 0 | -3.7 | -39.8 | 0 | 0 | 2 |  |
| 1981 | 9 | 11 | 11 | 20 | 0 | -4.3 | -38.4 | 0 | 0 | 2.7 |  |
| 1981 | 11 | 2 | 11 | 37 | 5 | -8.6 | -38.5 | 0 | 5 | 3.1 | 3-4 |
| 1981 | 11 | 2 | 14 | 57 | 13 | -8.6 | -38.5 | 0 | 5 | 3.1 | 4 |
| 1981 | 11 | 4 | 3 | 16 | 51 | -8.6 | -38.5 | 0 | 5 | 3.1 | 4 |
| 1982 | 3 | 13 | 18 | 18 | 0 | -4.3 | -38.4 | 0 | 0 | 2.7 |  |
| 1982 | 3 | 29 | 7 | 47 | 11 | -9.3 | -38.2 | 0 | 5 | 3.6 | 4 |
| 1982 | 7 | 23 | 11 | 6 | 45 | -14 | -45.7 | 0 | 50 | 2.9 |  |
| 1982 | 9 | 5 | 6 | 7 | 0 | -4.3 | -38.4 | 0 | 0 | 2.2 |  |
| 1982 | 10 | 5 | 16 | 19 | 32 | -4.5 | -40.2 | 0 | 50 | 2.1 |  |
| 1983 | 1 | 8 | 13 | 10 | 14 | -9.1 | -37.1 | 0 | 0 | 2.6 |  |
| 1983 | 3 | 22 | 12 | 51 | 21 | -6.1 | -36.4 | 0 | 0 | 2 |  |
| 1983 | 3 | 23 | 1 | 16 | 16 | -6.1 | -36.4 | 0 | 50 | 2.4 |  |
| 1983 | 5 | 4 | 21 | 53 | 5 | -5.1 | -38.7 | 0 | 50 | 2.4 |  |
| 1983 | 5 | 5 | 21 | 40 | 0 | -8.7 | -35.6 | 0 | 0 | 3 | 4 |
| 1983 | 5 | 7 | 18 | 30 | 0 | -8.7 | -35.6 | 0 | 0 | 3.5 | 5 |
| 1983 | 6 | 1 | 10 | 3 | 55 | -8.6 | -36.2 | 0 | 40 | 2.4 |  |
| 1983 | 6 | 1 | 10 | 49 | 20 | -8.6 | -36.2 | 0 | 40 | 2.3 |  |
| 1983 | 6 | 1 | 14 | 57 | 23 | -14 | -44.6 | 0 | 50 | 3.2 |  |
| 1983 | 6 | 1 | 17 | 18 | 6 | -8.6 | -36.2 | 0 | 40 | 2.5 |  |
| 1983 | 6 | 1 | 17 | 42 | 21 | -8.6 | -36.2 | 0 | 40 | 2.3 |  |
| 1983 | 6 | 1 | 18 | 12 | 24 | -8.5 | -42.8 | 0 | 0 | 2 |  |
| 1983 | 6 | 1 | 18 | 31 | 28 | -8.6 | -36.2 | 0 | 40 | 2.1 |  |
| 1983 | 6 | 28 | 20 | 10 | 15 | -13 | -39.3 | 0 | 50 | 2.2 |  |
| 1983 | 9 | 16 | 13 | 43 | 52 | -4.3 | -38.4 | 0 | 15 | 2.5 |  |
| 1983 | 10 | 1 | 6 | 14 | 35 | -4.7 | -39.8 | 0 | 50 | 2.7 |  |
| 1983 | 11 | 18 | 8 | 41 | 2 | -5.1 | -38.7 | 0 | 0 | 2.8 |  |
| 1983 | 11 | 18 | 10 | 27 | 41 | -5 | -38.7 | 0 | 40 | 2.3 |  |
| 1983 | 11 | 18 | 12 | 10 | 9 | -5 | -38.7 | 0 | 40 | 2.6 |  |
| 1983 | 12 | 16 | 23 | 53 | 35 | -8.3 | -35.8 | 0 | 15 | 3.5 |  |
| 1983 | 12 | 23 | 2 | 2 | 21 | -5.6 | -35.8 | 0 | 0 | 2.2 |  |
| 1983 | 12 | 25 | 1 | 4 | 9 | -5.6 | -35.8 | 0 | 10 | 2.3 | 4 |
| 1984 | 1 | 3 | 5 | 4 | 36 | -5.6 | -35.8 | 0 | 0 | 2.1 |  |
| 1984 | 1 | 3 | 6 | 31 | 17 | -5.6 | -35.8 | 0 | 0 | 2 |  |
| 1984 | 1 | 3 | 21 | 26 | 7 | -5.6 | -35.8 | 0 | 0 | 2 |  |
| 1984 | 5 | 18 | 19 | 9 | 4 | -6 | -36.2 | 0 | 50 | 2.6 |  |
| 1984 | 10 | 23 | 7 | 42 | 22 | -8.2 | -35.9 | 0 | 20 | 2.7 |  |
| 1984 | 11 | 8 | 12 | 5 | 35 | -8.2 | -35.9 | 0 | 5 | 3.8 | 5-6 |
| 1986 | 1 | 30 | 5 | 38 | 46 | -8.9 | -36.4 | 0 | 40 | 2.3 |  |
| 1986 | 3 | 24 | 20 | 57 | 20 | -8.4 | -35.9 | 0 | 30 | 2.4 |  |
| 1986 | 6 | 12 | 1 | 43 | 49 | -14 | -40.4 | 0 | 50 | 3 |  |
| 1986 | 6 | 14 | 6 | 6 | 55 | -6.9 | -38.6 | 0 | 0 | 2 |  |
| 1986 | 6 | 14 | 21 | 26 | 15 | -6.9 | -38.6 | 0 | 10 | 3.2 | 4 |
| 1986 | 8 | 5 | 23 | 13 | 30 | -5.5 | -35.8 | 0 | 10 | 3.3 | 5-6 |
| 1986 | 8 | 5 | 23 | 58 | 22 | -5.5 | -35.8 | 0 | 10 | 3 |  |
| 1986 | 8 | 5 | 23 | 59 | 19 | -5.5 | -35.8 | 0 | 10 | 3 |  |
| 1986 | 8 | 6 | 12 | 56 | 1 | -5.5 | -35.8 | 0 | 10 | 3 |  |
| 1986 | 8 | 20 | 11 | 58 | 10 | -5.5 | -35.8 | 0 | 10 | 3.3 |  |
| 1986 | 8 | 20 | 18 | 0 | 14 | -5.5 | -35.8 | 0 | 10 | 3.8 | 5 |
| 1986 | 8 | 20 | 18 | 12 | 14 | -5.5 | -35.8 | 0 | 10 | 3.2 |  |
| 1986 | 8 | 21 | 9 | 34 | 40 | -5.5 | -35.7 | 0 | 5 | 4.2 | 6-7 |
| 1986 | 8 | 21 | 9 | 41 | 45 | -5.5 | -35.8 | 0 | 10 | 3.2 |  |
| 1986 | 8 | 23 | 20 | 6 | 31 | -5.5 | -35.8 | 0 | 10 | 3.7 |  |
| 1986 | 8 | 25 | 14 | 52 | 12 | -5.5 | -35.8 | 0 | 10 | 3.1 |  |
| 1986 | 8 | 27 | 9 | 54 | 53 | -5.5 | -35.8 | 0 | 10 | 3 |  |
| 1986 | 8 | 27 | 13 | 25 | 26 | -5.5 | -35.8 | 0 | 10 | 3.1 |  |
| 1986 | 9 | 3 | 1 | 16 | 6 | -5.5 | -35.7 | 0 | 5 | 4.1 | 6 |
| 1986 | 9 | 3 | 3 | 38 | 18 | -5.5 | -35.8 | 0 | 10 | 3.4 |  |
| 1986 | 9 | 3 | 4 | 19 | 50 | -5.5 | -35.8 | 0 | 10 | 3.2 |  |
| 1986 | 9 | 4 | 1 | 15 | 39 | -5.5 | -35.8 | 0 | 10 | 3.1 |  |
| 1986 | 9 | 4 | 1 | 31 | 21 | -5.5 | -35.8 | 0 | 10 | 3 |  |
| 1986 | 9 | 5 | 20 | 39 | 28 | -5.5 | -35.8 | 0 | 5 | 4.1 | 6 |
| 1986 | 9 | 5 | 20 | 46 | 17 | -5.5 | -35.8 | 0 | 10 | 3.2 |  |
| 1986 | 9 | 5 | 20 | 57 | 23 | -5.5 | -35.8 | 0 | 10 | 3.1 |  |
| 1986 | 9 | 9 | 9 | 17 | 22 | -5.5 | -35.8 | 0 | 10 | 3 |  |
| 1986 | 9 | 9 | 20 | 2 | 36 | -5.5 | -35.8 | 0 | 10 | 3.6 |  |
| 1986 | 9 | 9 | 23 | 29 | 2 | -5.5 | -35.8 | 0 | 10 | 3.2 |  |
| 1986 | 9 | 11 | 9 | 5 | 41 | -5.5 | -35.8 | 0 | 10 | 3.6 |  |
| 1986 | 11 | 30 | 5 | 19 | 50 | -5.5 | -35.8 | 5 | 5 | 5.1 | 7 |
| 1986 | 11 | 30 | 5 | 26 | 10 | -5.5 | -35.8 | 0 | 10 | 3.9 |  |
| 1986 | 11 | 30 | 6 | 7 | 35 | -5.5 | -35.8 | 0 | 10 | 4 |  |
| 1986 | 11 | 30 | 6 | 10 | 20 | -5.5 | -35.8 | 0 | 10 | 4.2 |  |
| 1986 | 11 | 30 | 6 | 58 | 57 | -5.5 | -35.8 | 0 | 10 | 3.8 |  |
| 1986 | 11 | 30 | 7 | 2 | 5 | -5.5 | -35.8 | 0 | 10 | 4.2 |  |
| 1986 | 11 | 30 | 14 | 1 | 47 | -5.5 | -35.8 | 0 | 10 | 3.8 |  |
| 1986 | 11 | 30 | 23 | 1 | 16 | -5.5 | -35.8 | 0 | 10 | 3.3 |  |
| 1986 | 11 | 30 | 23 | 2 | 47 | -5.5 | -35.8 | 0 | 10 | 3.2 |  |
| 1986 | 11 | 30 | 23 | 3 | 59 | -5.5 | -35.8 | 0 | 10 | 3.3 |  |
| 1986 | 12 | 1 | 1 | 4 | 51 | -5.5 | -35.8 | 0 | 10 | 3.8 |  |
| 1986 | 12 | 1 | 17 | 37 | 5 | -5.5 | -35.8 | 0 | 10 | 3.3 |  |
| 1986 | 12 | 1 | 21 | 59 | 16 | -5.5 | -35.8 | 0 | 10 | 3.3 |  |
| 1986 | 12 | 2 | 7 | 6 | 12 | -5.5 | -35.8 | 0 | 10 | 3.2 |  |
| 1986 | 12 | 2 | 9 | 50 | 48 | -5.5 | -35.8 | 0 | 10 | 3.6 |  |
| 1986 | 12 | 3 | 8 | 1 | 46 | -5.5 | -35.8 | 0 | 10 | 3.2 |  |
| 1986 | 12 | 3 | 10 | 6 | 2 | -5.5 | -35.8 | 0 | 10 | 3.6 |  |
| 1986 | 12 | 3 | 21 | 55 | 37 | -5.5 | -35.8 | 0 | 10 | 3.1 |  |
| 1986 | 12 | 5 | 14 | 17 | 50 | -5.5 | -35.8 | 0 | 10 | 3.1 |  |
| 1986 | 12 | 5 | 22 | 10 | 8 | -5.5 | -35.8 | 0 | 10 | 4 |  |
| 1986 | 12 | 6 | 3 | 4 | 21 | -5.5 | -35.8 | 0 | 10 | 3.4 |  |
| 1986 | 12 | 6 | 9 | 31 | 32 | -5.5 | -35.8 | 0 | 10 | 3.6 |  |
| 1986 | 12 | 6 | 9 | 51 | 4 | -5.5 | -35.8 | 0 | 10 | 3.8 |  |
| 1986 | 12 | 7 | 20 | 18 | 9 | -5.5 | -35.8 | 0 | 10 | 3.3 |  |
| 1986 | 12 | 8 | 21 | 23 | 42 | -5.5 | -35.7 | 6 | 2 | 3.6 |  |
| 1986 | 12 | 8 | 21 | 49 | 3 | -5.5 | -35.7 | 6 | 3 | 3.5 |  |
| 1986 | 12 | 9 | 6 | 48 | 44 | -5.5 | -35.7 | 5 | 3 | 4.4 | 6 |
| 1986 | 12 | 11 | 14 | 23 | 1 | -5.5 | -35.8 | 0 | 10 | 3.2 |  |
| 1986 | 12 | 13 | 4 | 32 | 17 | -5.5 | -35.8 | 0 | 10 | 3 |  |
| 1986 | 12 | 16 | 8 | 20 | 16 | -5.5 | -35.8 | 0 | 10 | 3 |  |
| 1986 | 12 | 16 | 21 | 52 | 18 | -5.5 | -35.8 | 0 | 10 | 3.1 |  |
| 1986 | 12 | 20 | 2 | 5 | 43 | -5.5 | -35.8 | 0 | 10 | 3 |  |
| 1986 | 12 | 24 | 7 | 5 | 25 | -5.5 | -35.8 | 0 | 10 | 3.8 |  |
| 1986 | 12 | 24 | 23 | 18 | 50 | -5.5 | -35.8 | 0 | 10 | 3.4 |  |
| 1987 | 1 | 3 | 13 | 31 | 4 | -5.5 | -35.8 | 0 | 10 | 3.7 |  |
| 1987 | 1 | 4 | 2 | 24 | 48 | -5.5 | -35.8 | 0 | 10 | 3.5 |  |
| 1987 | 1 | 7 | 11 | 8 | 27 | -5.6 | -35.8 | 0 | 2 | 3.7 |  |
| 1987 | 1 | 7 | 12 | 14 | 16 | -5.6 | -35.8 | 0 | 2 | 3.8 |  |
| 1987 | 1 | 7 | 15 | 6 | 45 | -5.5 | -35.8 | 0 | 10 | 3.3 |  |
| 1987 | 1 | 13 | 6 | 38 | 59 | -5.5 | -35.8 | 0 | 10 | 3.3 |  |
| 1987 | 1 | 28 | 16 | 16 | 12 | -5.5 | -35.8 | 0 | 2 | 3.7 |  |
| 1987 | 1 | 30 | 2 | 3 | 0 | -5.5 | -35.8 | 0 | 10 | 3 |  |
| 1987 | 2 | 3 | 23 | 51 | 56 | -5.5 | -35.7 | 0 | 2 | 3.8 |  |
| 1987 | 2 | 7 | 7 | 48 | 14 | -5.5 | -35.8 | 0 | 10 | 3.4 |  |
| 1987 | 2 | 7 | 22 | 12 | 38 | -5.5 | -35.7 | 0 | 2 | 3.7 |  |
| 1987 | 2 | 23 | 20 | 1 | 37 | -5.5 | -35.8 | 0 | 10 | 3.1 |  |
| 1987 | 2 | 24 | 20 | 15 | 6 | -5.5 | -35.8 | 0 | 10 | 3.1 |  |
| 1987 | 2 | 25 | 21 | 37 | 4 | -3.7 | -39.8 | 0 | 50 | 3 | 4 |
| 1987 | 4 | 28 | 4 | 59 | 17 | -5.5 | -35.8 | 0 | 10 | 3.6 |  |
| 1987 | 5 | 17 | 5 | 32 | 15 | -5.7 | -36.1 | 0 | 50 | 2 |  |
| 1987 | 6 | 2 | 6 | 8 | 8 | -8.4 | -36.2 | 0 | 6 | 2 |  |
| 1987 | 6 | 2 | 6 | 29 | 36 | -8.4 | -36.2 | 0 | 6 | 2.3 |  |
| 1987 | 6 | 18 | 16 | 21 | 2 | -5.5 | -35.8 | 0 | 10 | 3 |  |
| 1987 | 6 | 27 | 5 | 9 | 2 | -5.5 | -35.8 | 0 | 10 | 3 |  |
| 1987 | 8 | 3 | 11 | 34 | 47 | -13 | -39.7 | 0 | 5 | 2.3 |  |
| 1987 | 11 | 30 | 1 | 23 | 56 | -5.5 | -35.8 | 0 | 10 | 3 |  |
| 1988 | 2 | 22 | 22 | 27 | 18 | -5 | -38.2 | 0 | 30 | 2 |  |
| 1988 | 3 | 14 | 6 | 11 | 42 | -4.7 | -38 | 0 | 30 | 2.2 |  |
| 1988 | 3 | 14 | 10 | 31 | 51 | -4.6 | -37.9 | 0 | 30 | 2.1 |  |
| 1988 | 3 | 14 | 23 | 43 | 33 | -4.6 | -38 | 0 | 30 | 2.1 |  |
| 1988 | 4 | 1 | 0 | 36 | 34 | -4 | -40.4 | 8 | 2 | 4.1 | 5-6 |
| 1988 | 4 | 1 | 0 | 37 | 46 | -4 | -40.4 | 8 | 2 | 3.9 |  |
| 1988 | 4 | 14 | 4 | 20 | 11 | -4.8 | -38.1 | 0 | 30 | 2.1 |  |
| 1988 | 4 | 30 | 7 | 33 | 16 | -4.7 | -38.3 | 0 | 30 | 2.5 |  |
| 1988 | 5 | 1 | 3 | 33 | 21 | -4.7 | -38.2 | 0 | 30 | 2.6 |  |
| 1988 | 6 | 25 | 11 | 56 | 26 | -5.5 | -35.8 | 0 | 15 | 3.4 |  |
| 1988 | 6 | 29 | 13 | 14 | 27 | -5.5 | -35.8 | 0 | 15 | 3.9 |  |
| 1988 | 10 | 1 | 1 | 39 | 40 | -5.5 | -35.8 | 0 | 15 | 3.3 |  |
| 1988 | 10 | 1 | 16 | 43 | 38 | -5.5 | -35.8 | 0 | 15 | 3 |  |
| 1988 | 10 | 3 | 1 | 3 | 35 | -5.5 | -35.8 | 0 | 15 | 3 |  |
| 1988 | 10 | 3 | 23 | 57 | 56 | -5.5 | -35.8 | 0 | 15 | 3.1 |  |
| 1988 | 10 | 4 | 15 | 23 | 33 | -5.5 | -35.8 | 0 | 15 | 3 |  |
| 1988 | 10 | 4 | 22 | 18 | 43 | -4.8 | -38 | 0 | 5 | 2.8 |  |
| 1988 | 10 | 18 | 9 | 44 | 37 | -4.8 | -38 | 4 | 2 | 3.7 |  |
| 1988 | 10 | 18 | 21 | 3 | 29 | -4.8 | -38 | 4 | 2 | 3.9 |  |
| 1988 | 10 | 19 | 2 | 15 | 51 | -4.8 | -38 | 4 | 2 | 4.2 | 6 |
| 1988 | 10 | 29 | 3 | 24 | 28 | -4.8 | -38 | 4 | 2 | 4.1 | 6 |
| 1988 | 10 | 29 | 15 | 30 | 7 | -4.8 | -38 | 0 | 4 | 3.3 |  |
| 1988 | 10 | 29 | 16 | 18 | 9 | -4.8 | -38 | 0 | 4 | 3 |  |
| 1988 | 10 | 30 | 7 | 13 | 4 | -4.8 | -38 | 5 | 2 | 3.2 |  |
| 1988 | 11 | 1 | 14 | 25 | 29 | -5.5 | -35.8 | 0 | 15 | 3.3 |  |
| 1988 | 11 | 10 | 20 | 15 | 3 | -4.8 | -38 | 0 | 5 | 3.3 |  |
| 1988 | 11 | 28 | 19 | 32 | 39 | -5.5 | -35.8 | 0 | 15 | 3 |  |
| 1988 | 12 | 10 | 10 | 28 | 48 | -4.8 | -38 | 0 | 5 | 3.4 |  |
| 1989 | 1 | 15 | 12 | 45 | 18 | -4.8 | -38 | 0 | 5 | 3.6 |  |
| 1989 | 2 | 7 | 19 | 12 | 45 | -4.8 | -38 | 0 | 5 | 3.1 |  |
| 1989 | 3 | 10 | 4 | 11 | 20 | -5.5 | -35.7 | 8 | 5 | 5 | 7 |
| 1989 | 3 | 10 | 7 | 13 | 27 | -5.5 | -35.7 | 0 | 10 | 3.2 |  |
| 1989 | 3 | 10 | 10 | 7 | 13 | -5.5 | -35.7 | 0 | 10 | 3.9 |  |
| 1989 | 3 | 10 | 18 | 54 | 52 | -5.5 | -35.7 | 0 | 10 | 3.3 |  |
| 1989 | 3 | 10 | 21 | 26 | 39 | -5.5 | -35.7 | 0 | 10 | 3.5 |  |
| 1989 | 3 | 14 | 22 | 56 | 35 | -5.5 | -35.7 | 0 | 10 | 3 |  |
| 1989 | 3 | 25 | 14 | 33 | 48 | -4.8 | -38 | 0 | 5 | 3.5 |  |
| 1989 | 3 | 25 | 15 | 29 | 24 | -4.8 | -38 | 4 | 5 | 4.1 |  |
| 1989 | 3 | 26 | 13 | 25 | 39 | -4.8 | -38 | 4 | 3 | 4.5 | 6 |
| 1989 | 3 | 26 | 16 | 30 | 16 | -4.8 | -38 | 4 | 5 | 3.7 |  |
| 1989 | 3 | 26 | 18 | 17 | 48 | -4.8 | -38 | 0 | 5 | 3.9 |  |
| 1989 | 5 | 6 | 19 | 55 | 46 | -5.5 | -35.7 | 0 | 20 | 3.6 |  |
| 1989 | 5 | 9 | 6 | 38 | 36 | -12 | -38.6 | 0 | 20 | 2.2 |  |
| 1989 | 5 | 13 | 7 | 20 | 46 | -4.8 | -38 | 0 | 10 | 3 |  |
| 1989 | 5 | 13 | 7 | 27 | 32 | -4.8 | -38 | 0 | 10 | 3.2 |  |
| 1989 | 5 | 22 | 21 | 9 | 46 | -5.5 | -35.7 | 0 | 20 | 3 |  |
| 1989 | 5 | 26 | 22 | 55 | 28 | -4.8 | -38 | 0 | 5 | 4.1 | 5-6 |
| 1989 | 8 | 26 | 16 | 5 | 47 | -4.8 | -38 | 0 | 5 | 3.8 |  |
| 1989 | 8 | 28 | 8 | 6 | 26 | -4.8 | -38 | 0 | 5 | 3.9 |  |
| 1989 | 9 | 27 | 3 | 56 | 28 | -5.8 | -36.9 | 0 | 5 | 2 |  |
| 1989 | 9 | 27 | 6 | 35 | 14 | -5.8 | -36.9 | 0 | 5 | 2.3 | 3-4 |
| 1989 | 9 | 27 | 6 | 50 | 5 | -5.8 | -36.9 | 0 | 5 | 2 |  |
| 1989 | 10 | 17 | 10 | 45 | 59 | -4.8 | -38 | 0 | 5 | 3.6 |  |
| 1989 | 10 | 17 | 15 | 2 | 48 | -4.8 | -38 | 0 | 5 | 4.2 |  |
| 1989 | 10 | 17 | 15 | 8 | 26 | -4.8 | -38 | 0 | 5 | 3.5 |  |
| 1989 | 10 | 17 | 15 | 26 | 34 | -4.8 | -38 | 0 | 10 | 3.1 |  |
| 1989 | 10 | 18 | 15 | 31 | 6 | -4.8 | -38 | 0 | 10 | 3 |  |
| 1989 | 11 | 6 | 15 | 14 | 0 | -7.1 | -41.4 | 0 | 0 | 2.6 | 3 |
| 1989 | 12 | 27 | 19 | 26 | 13 | -3.8 | -40.8 | 0 | 30 | 3.2 | 4-5 |
| 1990 | 3 | 8 | 21 | 1 | 32 | -16 | -40.9 | 1 | 2 | 3.1 | 4 |
| 1990 | 3 | 9 | 12 | 53 | 29 | -16 | -40.9 | 1 | 2 | 2.9 |  |
| 1990 | 4 | 30 | 5 | 31 | 4 | -13 | -40.3 | 0 | 10 | 2.9 | 4-5 |
| 1990 | 6 | 13 | 16 | 35 | 3 | -5.8 | -36.9 | 0 | 5 | 2.6 |  |
| 1990 | 6 | 13 | 21 | 57 | 36 | -5.8 | -36.9 | 0 | 5 | 2.1 |  |
| 1990 | 7 | 24 | 11 | 33 | 24 | -5.8 | -36.9 | 2 | 3 | 2.2 | 2-3 |
| 1990 | 9 | 7 | 23 | 39 | 41 | -5.7 | -35.9 | 3 | 5 | 2.8 |  |
| 1990 | 9 | 9 | 11 | 24 | 20 | -5.7 | -35.9 | 3 | 5 | 3 |  |
| 1990 | 9 | 12 | 20 | 34 | 18 | -5.7 | -35.9 | 3 | 5 | 3.2 |  |
| 1990 | 9 | 13 | 14 | 24 | 2 | -5.8 | -36.9 | 2 | 3 | 2.1 | 2-3 |
| 1990 | 9 | 24 | 15 | 25 | 18 | -5.7 | -35.9 | 3 | 5 | 3.7 | 5 |
| 1990 | 10 | 2 | 9 | 44 | 3 | -4.3 | -38.5 | 0 | 20 | 2 |  |
| 1990 | 10 | 15 | 6 | 27 | 23 | -5.7 | -39.5 | 0 | 30 | 2.5 |  |
| 1990 | 10 | 27 | 6 | 5 | 15 | -5.9 | -36.9 | 2 | 30 | 2.2 |  |
| 1990 | 11 | 1 | 19 | 26 | 2 | -5.6 | -39.7 | 0 | 30 | 2.1 |  |
| 1990 | 11 | 3 | 10 | 46 | 14 | -5.7 | -35.9 | 3 | 5 | 3.1 |  |
| 1990 | 11 | 10 | 21 | 43 | 3 | -5.9 | -37.3 | 4 | 5 | 2.1 | 3-4 |
| 1990 | 11 | 11 | 4 | 37 | 55 | -5.9 | -37.3 | 4 | 5 | 2.3 | 3-4 |
| 1990 | 12 | 19 | 2 | 5 | 55 | -11 | -40.4 | 0 | 30 | 2.1 |  |
| 1991 | 2 | 8 | 21 | 13 | 14 | -6.1 | -38.5 | 0 | 30 | 2.8 |  |
| 1991 | 2 | 17 | 9 | 34 | 33 | -6.1 | -38.5 | 0 | 30 | 2.1 |  |
| 1991 | 3 | 14 | 3 | 5 | 9 | -11 | -38.7 | 0 | 30 | 2.2 |  |
| 1991 | 4 | 14 | 15 | 45 | 0 | -9.8 | -37.4 | 0 | 5 | 2 | 4-5 |
| 1991 | 4 | 19 | 6 | 10 | 0 | -16 | -40.9 | 0 | 2 | 2.8 | 3-4 |
| 1991 | 4 | 19 | 10 | 12 | 49 | -3.9 | -39.9 | 10 | 5 | 4.9 | 6-7 |
| 1991 | 4 | 26 | 14 | 14 | 29 | -5.5 | -35.7 | 0 | 10 | 3.5 |  |
| 1991 | 5 | 5 | 21 | 7 | 57 | -15 | -39.4 | 0 | 50 | 2.8 |  |
| 1991 | 5 | 12 | 6 | 50 | 44 | -9 | -41.8 | 0 | 50 | 2.9 |  |
| 1991 | 5 | 27 | 5 | 55 | 9 | -4.4 | -40.3 | 1 | 1 | 2.4 |  |
| 1991 | 9 | 6 | 19 | 45 | 20 | -8.3 | -36 | 0 | 5 | 2 |  |
| 1991 | 9 | 11 | 3 | 55 | 19 | -13 | -38.7 | 0 | 20 | 2.3 |  |
| 1991 | 9 | 13 | 19 | 56 | 41 | -8.3 | -36 | 0 | 5 | 2.1 |  |
| 1991 | 9 | 17 | 8 | 24 | 26 | -12 | -40.6 | 0 | 40 | 2.4 |  |
| 1991 | 9 | 21 | 18 | 27 | 37 | -8.3 | -36 | 0 | 5 | 2.5 |  |
| 1991 | 10 | 15 | 10 | 33 | 0 | -9.6 | -40.3 | 0 | 20 | 2 |  |
| 1991 | 11 | 22 | 8 | 5 | 55 | -13 | -38.7 | 0 | 20 | 2.2 | 4-5 |
| 1992 | 2 | 6 | 7 | 38 | 47 | -10 | -36.9 | 0 | 30 | 2.8 |  |
| 1992 | 2 | 17 | 5 | 14 | 20 | -5.5 | -35.7 | 0 | 5 | 3 |  |
| 1992 | 4 | 17 | 21 | 28 | 8 | -5.6 | -35.9 | 0 | 5 | 3.2 |  |
| 1992 | 6 | 9 | 10 | 27 | 55 | -14 | -39.8 | 0 | 40 | 3 |  |
| 1992 | 8 | 5 | 17 | 37 | 21 | -12 | -40.6 | 0 | 40 | 2.1 |  |
| 1992 | 8 | 6 | 3 | 47 | 51 | -12 | -40.6 | 0 | 40 | 2.3 |  |
| 1992 | 8 | 13 | 18 | 20 | 31 | -12 | -40.6 | 0 | 40 | 2.8 |  |
| 1992 | 8 | 16 | 6 | 57 | 21 | -12 | -40.6 | 0 | 40 | 2.1 |  |
| 1992 | 8 | 20 | 8 | 8 | 43 | -12 | -40.6 | 0 | 40 | 2.4 |  |
| 1992 | 8 | 20 | 8 | 19 | 29 | -12 | -40.6 | 0 | 40 | 2.3 |  |
| 1992 | 8 | 20 | 19 | 23 | 34 | -10 | -40 | 0 | 20 | 2.5 |  |
| 1992 | 8 | 25 | 17 | 26 | 26 | -13 | -39 | 0 | 50 | 3.2 |  |
| 1992 | 9 | 2 | 11 | 24 | 25 | -12 | -40.6 | 0 | 40 | 2 |  |
| 1992 | 9 | 10 | 0 | 56 | 31 | -12 | -40.6 | 0 | 40 | 2.4 |  |
| 1992 | 9 | 19 | 23 | 22 | 52 | -12 | -40.6 | 0 | 40 | 2.4 |  |
| 1992 | 9 | 19 | 23 | 33 | 56 | -12 | -40.6 | 0 | 40 | 2.1 |  |
| 1992 | 9 | 20 | 8 | 39 | 47 | -12 | -40.6 | 0 | 40 | 2.2 |  |
| 1992 | 9 | 30 | 12 | 57 | 45 | -12 | -40.6 | 0 | 40 | 2.1 |  |
| 1992 | 10 | 15 | 8 | 56 | 19 | -12 | -40.6 | 0 | 40 | 2.6 |  |
| 1993 | 1 | 1 | 23 | 22 | 9 | -6.8 | -37.1 | 0 | 40 | 2.4 |  |
| 1993 | 1 | 28 | 3 | 50 | 42 | -5.6 | -35.8 | 0 | 10 | 2.6 |  |
| 1993 | 1 | 28 | 11 | 23 | 51 | -4.8 | -38 | 0 | 5 | 2 |  |
| 1993 | 2 | 1 | 16 | 9 | 24 | -5.6 | -35.8 | 0 | 10 | 3 |  |
| 1993 | 2 | 3 | 21 | 14 | 30 | -5.6 | -35.8 | 0 | 10 | 2.7 |  |
| 1993 | 2 | 5 | 5 | 41 | 52 | -8.3 | -36 | 4 | 5 | 2.2 |  |
| 1993 | 2 | 16 | 10 | 21 | 12 | -5.7 | -36.3 | 0 | 5 | 2.3 | 4 |
| 1993 | 4 | 13 | 15 | 15 | 35 | -2.9 | -39.5 | 0 | 20 | 2.4 |  |
| 1993 | 4 | 14 | 17 | 32 | 17 | -3.3 | -40.5 | 0 | 15 | 2.1 |  |
| 1993 | 4 | 15 | 10 | 22 | 33 | -3.8 | -40.1 | 0 | 5 | 2.4 | 3-4 |
| 1993 | 4 | 16 | 11 | 38 | 58 | -3.8 | -40.1 | 0 | 5 | 2.5 | 3-4 |
| 1993 | 8 | 29 | 0 | 48 | 38 | -5.9 | -38.1 | 0 | 2 | 2.2 | 3-4 |
| 1993 | 9 | 25 | 20 | 27 | 27 | -10 | -37.1 | 0 | 10 | 3.5 | 5 |
| 1993 | 9 | 27 | 3 | 56 | 6 | -10 | -37.1 | 0 | 10 | 3.5 | 5-6 |
| 1993 | 10 | 5 | 19 | 31 | 48 | -8.3 | -36 | 0 | 5 | 2.1 |  |
| 1993 | 10 | 6 | 7 | 23 | 11 | -8.3 | -36 | 0 | 5 | 2.5 |  |
| 1993 | 10 | 10 | 0 | 58 | 17 | -8.3 | -36 | 0 | 5 | 3.3 | 3-4 |
| 1993 | 10 | 23 | 20 | 21 | 50 | -4.4 | -38.3 | 0 | 2 | 2.1 | 3-4 |
| 1993 | 10 | 24 | 20 | 59 | 36 | -4.4 | -38.3 | 0 | 2 | 2.1 | 3-4 |
| 1993 | 11 | 21 | 2 | 56 | 13 | -4.4 | -38.3 | 0 | 2 | 2 | 3-4 |
| 1994 | 2 | 18 | 1 | 55 | 19 | -5.7 | -35.9 | 0 | 10 | 2 |  |
| 1994 | 2 | 20 | 4 | 53 | 40 | -5.7 | -35.9 | 0 | 10 | 2 |  |
| 1994 | 2 | 20 | 6 | 4 | 47 | -5.7 | -35.9 | 0 | 10 | 2 |  |
| 1994 | 2 | 20 | 12 | 23 | 59 | -5.7 | -35.9 | 0 | 10 | 2 |  |
| 1994 | 2 | 21 | 23 | 11 | 17 | -4.4 | -38.3 | 4 | 2 | 2 |  |
| 1994 | 2 | 22 | 13 | 53 | 48 | -4.4 | -38.3 | 4 | 2 | 2.2 |  |
| 1994 | 2 | 24 | 18 | 22 | 59 | -4.4 | -38.3 | 4 | 2 | 2.1 |  |
| 1994 | 4 | 6 | 12 | 18 | 22 | -9.8 | -36.5 | 0 | 20 | 2.8 | 4 |
| 1994 | 6 | 13 | 16 | 4 | 53 | -4.4 | -38.3 | 4 | 2 | 2.1 |  |
| 1994 | 6 | 14 | 14 | 44 | 41 | -4.4 | -38.3 | 4 | 2 | 2.1 |  |
| 1994 | 6 | 14 | 18 | 40 | 7 | -4.4 | -38.3 | 4 | 2 | 2.1 |  |
| 1994 | 6 | 14 | 20 | 18 | 8 | -4.4 | -38.3 | 4 | 2 | 2 |  |
| 1994 | 6 | 15 | 14 | 8 | 7 | -4.4 | -38.3 | 4 | 2 | 2.2 |  |
| 1994 | 6 | 16 | 11 | 57 | 43 | -4.4 | -38.3 | 4 | 2 | 2.1 |  |
| 1994 | 6 | 18 | 21 | 2 | 7 | -4.4 | -38.3 | 4 | 2 | 2.9 |  |
| 1994 | 6 | 19 | 2 | 33 | 55 | -4.4 | -38.3 | 4 | 2 | 2.2 |  |
| 1994 | 6 | 19 | 19 | 33 | 20 | -4.4 | -38.3 | 4 | 2 | 2.4 |  |
| 1994 | 6 | 19 | 19 | 35 | 3 | -4.4 | -38.3 | 4 | 2 | 2 |  |
| 1994 | 6 | 20 | 18 | 15 | 44 | -4.4 | -38.3 | 4 | 2 | 2.6 |  |
| 1994 | 6 | 21 | 2 | 0 | 1 | -4.4 | -38.3 | 4 | 2 | 2.6 |  |
| 1994 | 6 | 21 | 12 | 31 | 24 | -5.5 | -35.7 | 0 | 10 | 2.3 |  |
| 1994 | 6 | 22 | 1 | 30 | 47 | -4.4 | -38.3 | 4 | 2 | 2 |  |
| 1994 | 6 | 23 | 7 | 32 | 37 | -4.4 | -38.3 | 4 | 2 | 2.5 |  |
| 1994 | 6 | 23 | 7 | 44 | 31 | -4.4 | -38.3 | 4 | 2 | 2.5 |  |
| 1994 | 6 | 23 | 7 | 45 | 59 | -4.4 | -38.3 | 4 | 2 | 2.5 |  |
| 1994 | 6 | 23 | 18 | 45 | 34 | -4.4 | -38.3 | 4 | 2 | 2 |  |
| 1994 | 6 | 23 | 23 | 15 | 16 | -4.4 | -38.3 | 4 | 2 | 2.2 |  |
| 1994 | 7 | 3 | 10 | 4 | 34 | -4.4 | -38.3 | 4 | 2 | 2.3 |  |
| 1994 | 7 | 7 | 8 | 9 | 45 | -4.4 | -38.3 | 4 | 2 | 2.4 |  |
| 1994 | 7 | 15 | 4 | 35 | 57 | -4.4 | -38.3 | 4 | 2 | 2.1 |  |
| 1994 | 7 | 24 | 9 | 32 | 11 | -4.4 | -38.3 | 4 | 2 | 2.2 |  |
| 1994 | 7 | 24 | 11 | 21 | 1 | -4.4 | -38.3 | 4 | 2 | 2.5 |  |
| 1994 | 7 | 24 | 11 | 30 | 53 | -4.4 | -38.3 | 4 | 2 | 2.4 |  |
| 1994 | 7 | 24 | 12 | 12 | 16 | -4.4 | -38.3 | 4 | 2 | 2.7 |  |
| 1994 | 7 | 24 | 13 | 56 | 2 | -4.4 | -38.3 | 4 | 2 | 2.9 |  |
| 1994 | 7 | 25 | 19 | 1 | 3 | -4.4 | -38.3 | 4 | 2 | 2.2 |  |
| 1994 | 7 | 29 | 16 | 3 | 16 | -4.4 | -38.3 | 4 | 2 | 2 |  |
| 1994 | 7 | 30 | 0 | 15 | 47 | -4.4 | -38.3 | 4 | 2 | 2 |  |
| 1994 | 8 | 1 | 20 | 38 | 4 | -4.4 | -38.3 | 4 | 2 | 2.3 |  |
| 1994 | 8 | 3 | 15 | 38 | 38 | -4.4 | -38.3 | 4 | 2 | 2 |  |
| 1994 | 8 | 5 | 10 | 55 | 17 | -4.4 | -38.3 | 4 | 2 | 2 |  |
| 1994 | 8 | 5 | 12 | 56 | 3 | -4.4 | -38.3 | 4 | 2 | 2.8 |  |
| 1994 | 8 | 5 | 18 | 42 | 36 | -4.4 | -38.3 | 4 | 2 | 2 |  |
| 1994 | 8 | 6 | 22 | 19 | 31 | -11 | -37.3 | 0 | 50 | 2.6 |  |
| 1994 | 8 | 8 | 2 | 50 | 35 | -4.4 | -38.3 | 4 | 2 | 2.2 |  |
| 1994 | 8 | 8 | 14 | 14 | 22 | -4.4 | -38.3 | 4 | 2 | 2.4 |  |
| 1994 | 8 | 8 | 15 | 6 | 10 | -4.4 | -38.3 | 4 | 2 | 2.4 |  |
| 1994 | 8 | 8 | 16 | 20 | 5 | -4.4 | -38.3 | 4 | 2 | 2.2 |  |
| 1994 | 8 | 10 | 2 | 41 | 42 | -4.4 | -38.3 | 4 | 2 | 2.2 |  |
| 1994 | 8 | 11 | 7 | 23 | 34 | -4.4 | -38.3 | 4 | 2 | 3.5 |  |
| 1994 | 8 | 11 | 7 | 27 | 57 | -4.4 | -38.3 | 4 | 2 | 2.4 |  |
| 1994 | 8 | 11 | 15 | 1 | 1 | -4.4 | -38.3 | 4 | 2 | 2 |  |
| 1994 | 8 | 15 | 8 | 12 | 28 | -4.4 | -38.3 | 4 | 2 | 2 |  |
| 1994 | 8 | 15 | 10 | 34 | 7 | -4.4 | -38.3 | 4 | 2 | 2.1 |  |
| 1994 | 8 | 24 | 7 | 1 | 14 | -4.4 | -38.3 | 4 | 2 | 2 |  |
| 1994 | 8 | 26 | 1 | 31 | 47 | -5.5 | -36.5 | 0 | 2 | 3 |  |
| 1994 | 8 | 26 | 7 | 52 | 30 | -5.5 | -36.5 | 0 | 2 | 2.6 |  |
| 1994 | 9 | 11 | 1 | 41 | 22 | -5.5 | -35.7 | 0 | 10 | 3.5 |  |
| 1994 | 9 | 16 | 10 | 36 | 0 | -5.5 | -35.7 | 0 | 10 | 2.2 |  |
| 1994 | 9 | 20 | 7 | 9 | 46 | -4.4 | -38.3 | 4 | 2 | 2.8 |  |
| 1994 | 9 | 21 | 6 | 31 | 17 | -4.4 | -38.3 | 4 | 2 | 2 |  |
| 1994 | 9 | 22 | 20 | 11 | 0 | -4.4 | -38.3 | 4 | 2 | 2.3 |  |
| 1994 | 9 | 23 | 1 | 5 | 17 | -13 | -39.7 | 0 | 40 | 2.7 | 3-4 |
| 1994 | 9 | 29 | 18 | 16 | 45 | -4.4 | -38.3 | 4 | 2 | 2.3 |  |
| 1994 | 10 | 2 | 22 | 38 | 48 | -4.4 | -38.3 | 4 | 2 | 2.1 |  |
| 1994 | 10 | 2 | 23 | 17 | 48 | -4.4 | -38.3 | 4 | 2 | 2.1 |  |
| 1994 | 10 | 23 | 11 | 39 | 33 | -4.4 | -38.3 | 4 | 2 | 2.1 |  |
| 1994 | 10 | 27 | 0 | 28 | 19 | -4.4 | -38.3 | 4 | 2 | 2 |  |
| 1994 | 11 | 1 | 14 | 42 | 41 | -5.7 | -35.9 | 0 | 10 | 2.2 |  |
| 1994 | 11 | 4 | 12 | 19 | 47 | -4.4 | -38.3 | 4 | 2 | 2.8 |  |
| 1994 | 11 | 15 | 3 | 10 | 44 | -4.4 | -38.3 | 4 | 2 | 2.5 |  |
| 1995 | 1 | 13 | 18 | 22 | 0 | -4.4 | -38.3 | 0 | 2 | 2.2 |  |
| 1995 | 1 | 13 | 20 | 46 | 0 | -4.4 | -38.3 | 0 | 2 | 2.6 |  |
| 1995 | 1 | 18 | 19 | 21 | 8 | -4.4 | -38.3 | 0 | 2 | 2.5 |  |
| 1995 | 1 | 18 | 20 | 45 | 7 | -4.4 | -38.3 | 0 | 2 | 2.2 |  |
| 1995 | 1 | 18 | 21 | 45 | 0 | -4.4 | -38.3 | 0 | 2 | 2.6 |  |
| 1995 | 1 | 20 | 14 | 21 | 47 | -4.4 | -38.3 | 0 | 2 | 2.1 |  |
| 1995 | 3 | 12 | 14 | 42 | 8 | -5.6 | -35.9 | 0 | 30 | 2.3 |  |
| 1995 | 3 | 20 | 3 | 26 | 38 | -5.4 | -35.9 | 0 | 30 | 2.9 |  |
| 1995 | 3 | 20 | 11 | 0 | 0 | -5.5 | -35.8 | 0 | 30 | 2.1 |  |
| 1995 | 3 | 29 | 0 | 41 | 36 | -7.1 | -40.2 | 0 | 30 | 2.6 |  |
| 1995 | 4 | 12 | 17 | 28 | 1 | -12 | -40.4 | 0 | 50 | 2.2 |  |
| 1995 | 4 | 22 | 16 | 50 | 50 | -4.4 | -38.3 | 4 | 2 | 3.5 |  |
| 1995 | 4 | 23 | 1 | 36 | 57 | -4.7 | -38.2 | 0 | 20 | 2.7 |  |
| 1995 | 4 | 28 | 13 | 33 | 8 | -5.6 | -35.6 | 0 | 10 | 2.6 |  |
| 1995 | 5 | 10 | 1 | 37 | 56 | -3.9 | -40.4 | 0 | 5 | 3.4 |  |
| 1995 | 5 | 30 | 1 | 41 | 13 | -11 | -38.2 | 0 | 50 | 2.2 |  |
| 1995 | 6 | 27 | 0 | 14 | 50 | -5.5 | -35.8 | 0 | 30 | 2 |  |
| 1995 | 6 | 27 | 4 | 13 | 53 | -5.5 | -35.8 | 0 | 30 | 2.1 |  |
| 1995 | 6 | 27 | 4 | 14 | 0 | -5.5 | -35.8 | 0 | 30 | 2 |  |
| 1995 | 7 | 18 | 9 | 40 | 24 | -5.5 | -35.8 | 0 | 30 | 2 |  |
| 1995 | 8 | 24 | 21 | 31 | 10 | -5.5 | -35.8 | 0 | 30 | 2.5 |  |
| 1995 | 10 | 12 | 10 | 45 | 0 | -4.4 | -38.3 | 0 | 2 | 2.5 |  |
| 1995 | 10 | 30 | 2 | 37 | 15 | -5.5 | -35.8 | 0 | 30 | 2.3 |  |
| 1995 | 11 | 25 | 6 | 22 | 3 | -5.5 | -35.8 | 0 | 30 | 2.2 |  |
| 1995 | 11 | 25 | 19 | 12 | 49 | -5.5 | -35.8 | 0 | 30 | 2.5 |  |
| 1995 | 12 | 27 | 9 | 53 | 44 | -5.5 | -35.8 | 0 | 30 | 2.4 |  |
| 1995 | 12 | 27 | 21 | 29 | 14 | -5.5 | -35.8 | 0 | 30 | 2 |  |
| 1996 | 1 | 19 | 16 | 18 | 30 | -5.5 | -35.7 | 0 | 10 | 2.7 |  |
| 1996 | 1 | 19 | 16 | 21 | 31 | -5.5 | -35.7 | 0 | 10 | 2.3 |  |
| 1996 | 6 | 13 | 13 | 15 | 0 | -15 | -40.3 | 0 | 10 | 2.1 | 2 |
| 1996 | 9 | 10 | 2 | 55 | 0 | -8.3 | -36 | 0 | 20 | 2.8 | 3-4 |
| 1996 | 9 | 20 | 21 | 8 | 16 | -5.5 | -35.7 | 0 | 10 | 2.6 |  |
| 1996 | 9 | 26 | 21 | 23 | 20 | -5.5 | -35.7 | 0 | 10 | 2 |  |
| 1996 | 11 | 14 | 4 | 30 | 0 | -8.3 | -36 | 0 | 20 | 2.3 | 2-3 |
| 1996 | 11 | 15 | 8 | 10 | 46 | -11 | -38.4 | 0 | 10 | 2.6 | 3 |
| 1997 | 1 | 15 | 9 | 32 | 27 | -5.5 | -35.7 | 0 | 5 | 2.3 |  |
| 1997 | 2 | 3 | 4 | 15 | 31 | -4.4 | -38.3 | 0 | 2 | 2.1 |  |
| 1997 | 2 | 3 | 4 | 16 | 6 | -4.4 | -38.3 | 0 | 2 | 2.1 |  |
| 1997 | 2 | 7 | 18 | 26 | 9 | -4.4 | -38.3 | 0 | 2 | 2.2 |  |
| 1997 | 2 | 16 | 6 | 58 | 31 | -4.3 | -39.5 | 0 | 30 | 2.3 |  |
| 1997 | 2 | 16 | 9 | 45 | 29 | -4.3 | -39.5 | 0 | 30 | 2 |  |
| 1997 | 3 | 2 | 12 | 43 | 23 | -4.4 | -38.3 | 0 | 2 | 2.2 |  |
| 1997 | 3 | 3 | 5 | 48 | 43 | -4.4 | -38.3 | 0 | 2 | 2 |  |
| 1997 | 3 | 4 | 16 | 30 | 8 | -4.4 | -38.3 | 0 | 2 | 2 |  |
| 1997 | 3 | 18 | 1 | 31 | 0 | -5.5 | -35.7 | 0 | 5 | 2 |  |
| 1997 | 3 | 20 | 19 | 12 | 25 | -5.5 | -35.8 | 0 | 5 | 2.1 |  |
| 1997 | 3 | 31 | 19 | 38 | 47 | -4.4 | -38.3 | 0 | 2 | 2.1 |  |
| 1997 | 4 | 5 | 11 | 49 | 58 | -5.5 | -35.7 | 0 | 5 | 2.4 |  |
| 1997 | 4 | 5 | 13 | 4 | 29 | -5.5 | -35.7 | 0 | 5 | 2.3 |  |
| 1997 | 4 | 5 | 13 | 21 | 27 | -5.5 | -35.7 | 0 | 5 | 2.3 |  |
| 1997 | 4 | 22 | 14 | 29 | 39 | -3.8 | -40.8 | 0 | 10 | 3.2 | 4-5 |
| 1997 | 4 | 22 | 18 | 52 | 26 | -4.4 | -38.3 | 0 | 2 | 2.1 |  |
| 1997 | 4 | 23 | 3 | 55 | 25 | -4.4 | -38.3 | 0 | 2 | 2.7 | 4 |
| 1997 | 6 | 4 | 6 | 7 | 23 | -3.8 | -40.8 | 0 | 10 | 2.4 | 2-3 |
| 1997 | 6 | 9 | 12 | 2 | 16 | -3.2 | -40.4 | 0 | 2 | 3.2 | 5 |
| 1997 | 6 | 23 | 7 | 21 | 31 | -5.5 | -35.7 | 0 | 5 | 2.5 |  |
| 1997 | 7 | 7 | 17 | 20 | 17 | -3.2 | -40.4 | 0 | 2 | 2.4 |  |
| 1997 | 7 | 18 | 7 | 16 | 10 | -5.5 | -35.7 | 0 | 5 | 2.7 |  |
| 1997 | 8 | 2 | 7 | 41 | 23 | -3.2 | -40.4 | 0 | 2 | 2 |  |
| 1997 | 9 | 7 | 6 | 8 | 54 | -4.4 | -38.3 | 0 | 2 | 2.1 |  |
| 1997 | 9 | 7 | 9 | 34 | 55 | -4.4 | -38.3 | 0 | 2 | 2.2 |  |
| 1997 | 9 | 8 | 10 | 58 | 33 | -4.4 | -38.3 | 0 | 2 | 2.2 |  |
| 1997 | 9 | 10 | 13 | 55 | 56 | -5.5 | -35.7 | 0 | 5 | 2 |  |
| 1997 | 9 | 12 | 5 | 34 | 33 | -4.4 | -38.3 | 0 | 2 | 2 |  |
| 1997 | 9 | 14 | 11 | 23 | 9 | -4.4 | -38.3 | 0 | 2 | 2.1 |  |
| 1997 | 9 | 17 | 9 | 18 | 36 | -4.4 | -38.3 | 0 | 2 | 2.2 |  |
| 1997 | 9 | 18 | 11 | 26 | 42 | -4.4 | -38.3 | 0 | 2 | 2 |  |
| 1997 | 9 | 19 | 2 | 26 | 36 | -4.4 | -38.3 | 0 | 2 | 2 |  |
| 1997 | 9 | 19 | 19 | 34 | 31 | -4.4 | -38.3 | 0 | 2 | 2.5 |  |
| 1997 | 9 | 19 | 22 | 3 | 22 | -4.4 | -38.3 | 0 | 2 | 3.6 |  |
| 1997 | 9 | 19 | 22 | 41 | 27 | -4.4 | -38.3 | 0 | 2 | 2 |  |
| 1997 | 9 | 21 | 4 | 14 | 55 | -4.4 | -38.3 | 0 | 2 | 3.3 |  |
| 1997 | 9 | 22 | 14 | 51 | 28 | -4.4 | -38.3 | 0 | 2 | 2 |  |
| 1997 | 9 | 22 | 16 | 5 | 47 | -4.4 | -38.3 | 0 | 2 | 2.8 |  |
| 1997 | 9 | 22 | 21 | 48 | 38 | -4.4 | -38.3 | 0 | 2 | 3.3 |  |
| 1997 | 9 | 23 | 4 | 41 | 32 | -5.5 | -35.7 | 0 | 5 | 2.4 |  |
| 1997 | 9 | 23 | 5 | 52 | 16 | -4.4 | -38.3 | 0 | 2 | 2.3 |  |
| 1997 | 9 | 23 | 6 | 21 | 0 | -5.5 | -38.2 | 0 | 5 | 2.2 |  |
| 1997 | 9 | 23 | 15 | 3 | 39 | -5.5 | -38.2 | 0 | 5 | 2.2 |  |
| 1997 | 9 | 23 | 23 | 6 | 58 | -4.4 | -38.3 | 0 | 2 | 2 |  |
| 1997 | 9 | 24 | 12 | 31 | 2 | -4.4 | -38.3 | 0 | 2 | 2 |  |
| 1997 | 9 | 24 | 18 | 43 | 29 | -4.4 | -38.3 | 0 | 2 | 2.2 |  |
| 1997 | 9 | 26 | 6 | 11 | 28 | -4.4 | -38.3 | 0 | 2 | 2 |  |
| 1997 | 9 | 26 | 13 | 13 | 18 | -4.4 | -38.3 | 0 | 2 | 2 |  |
| 1997 | 9 | 26 | 18 | 11 | 23 | -4.4 | -38.3 | 0 | 2 | 2.7 |  |
| 1997 | 9 | 27 | 16 | 46 | 38 | -3.2 | -40.4 | 0 | 2 | 2 |  |
| 1997 | 9 | 29 | 18 | 33 | 38 | -4.4 | -38.3 | 0 | 2 | 2.3 |  |
| 1997 | 10 | 5 | 5 | 14 | 17 | -4.4 | -38.3 | 0 | 2 | 2 |  |
| 1997 | 10 | 9 | 8 | 43 | 15 | -4.4 | -38.3 | 0 | 2 | 2.2 |  |
| 1997 | 10 | 9 | 9 | 16 | 23 | -4.4 | -38.3 | 0 | 2 | 2.3 |  |
| 1997 | 10 | 23 | 23 | 15 | 6 | -3.9 | -39.5 | 0 | 30 | 2.9 | 3-4 |
| 1997 | 12 | 11 | 10 | 48 | 55 | -4.4 | -38.3 | 0 | 2 | 2.4 |  |
| 1997 | 12 | 11 | 12 | 47 | 37 | -5.7 | -35.9 | 0 | 5 | 2.2 |  |
| 1997 | 12 | 11 | 22 | 16 | 57 | -4.4 | -38.3 | 0 | 2 | 2.3 |  |
| 1997 | 12 | 12 | 4 | 54 | 33 | -4.4 | -38.3 | 0 | 2 | 2.6 |  |
| 1997 | 12 | 12 | 8 | 45 | 45 | -3.2 | -40.4 | 0 | 2 | 3 |  |
| 1997 | 12 | 12 | 9 | 55 | 58 | -4.4 | -38.3 | 0 | 2 | 2 |  |
| 1997 | 12 | 12 | 11 | 58 | 47 | -4.4 | -38.3 | 0 | 2 | 2.1 |  |
| 1997 | 12 | 13 | 22 | 9 | 50 | -4.4 | -38.3 | 0 | 2 | 2.3 |  |
| 1997 | 12 | 16 | 3 | 34 | 2 | -4.4 | -38.3 | 0 | 2 | 3.1 |  |
| 1997 | 12 | 20 | 22 | 5 | 28 | -4.4 | -38.3 | 0 | 2 | 2.6 |  |
| 1998 | 1 | 1 | 12 | 23 | 46 | -5.7 | -35.9 | 0 | 5 | 2 |  |
| 1998 | 1 | 4 | 7 | 59 | 23 | -5.7 | -35.9 | 0 | 5 | 2 |  |
| 1998 | 1 | 22 | 5 | 49 | 19 | -3.9 | -39.5 | 0 | 10 | 2.1 |  |
| 1998 | 2 | 2 | 15 | 31 | 25 | -3.9 | -39.5 | 0 | 10 | 2.8 |  |
| 1998 | 2 | 13 | 19 | 35 | 37 | -5.5 | -35.7 | 0 | 5 | 2 |  |
| 1998 | 3 | 31 | 12 | 49 | 35 | -3.9 | -39.4 | 0 | 3 | 2.1 |  |
| 1998 | 4 | 1 | 8 | 52 | 1 | -3.9 | -39.4 | 0 | 3 | 2.1 |  |
| 1998 | 4 | 7 | 20 | 24 | 41 | -5.6 | -35.8 | 0 | 5 | 2.1 |  |
| 1998 | 4 | 22 | 2 | 29 | 10 | -4.4 | -38.3 | 0 | 2 | 2.8 |  |
| 1998 | 4 | 28 | 17 | 14 | 49 | -5.7 | -35.9 | 0 | 5 | 2 |  |
| 1998 | 4 | 29 | 3 | 18 | 28 | -4.4 | -38.3 | 0 | 2 | 2.1 |  |
| 1998 | 5 | 20 | 18 | 13 | 17 | -5.6 | -35.8 | 0 | 5 | 2.1 |  |
| 1998 | 6 | 2 | 23 | 18 | 47 | -4.4 | -38.3 | 0 | 2 | 2 |  |
| 1998 | 6 | 4 | 0 | 40 | 46 | -4.4 | -38.3 | 0 | 2 | 4 |  |
| 1998 | 6 | 4 | 0 | 48 | 22 | -4.4 | -38.3 | 0 | 2 | 2.5 |  |
| 1998 | 6 | 4 | 11 | 31 | 30 | -4.4 | -38.3 | 0 | 2 | 2 |  |
| 1998 | 6 | 4 | 15 | 16 | 27 | -4.4 | -38.3 | 0 | 2 | 2.3 |  |
| 1998 | 6 | 9 | 3 | 18 | 20 | -4.4 | -38.3 | 0 | 2 | 2.3 |  |
| 1998 | 6 | 11 | 1 | 21 | 52 | -4.4 | -38.3 | 0 | 2 | 2.4 |  |
| 1998 | 6 | 11 | 1 | 41 | 47 | -4.4 | -38.3 | 0 | 2 | 2.2 |  |
| 1998 | 6 | 11 | 2 | 1 | 51 | -4.4 | -38.3 | 0 | 2 | 2 |  |
| 1998 | 7 | 2 | 13 | 30 | 25 | -4.4 | -38.3 | 0 | 2 | 2.4 |  |
| 1998 | 7 | 3 | 17 | 23 | 35 | -4.4 | -38.3 | 0 | 2 | 2.5 |  |
| 1998 | 7 | 10 | 16 | 36 | 41 | -4.4 | -38.3 | 0 | 2 | 2.3 |  |
| 1998 | 7 | 29 | 6 | 20 | 52 | -9.9 | -39.9 | 0 | 1 | 3.1 | 3 |
| 1998 | 8 | 4 | 6 | 2 | 41 | -4.4 | -38.3 | 0 | 2 | 2.8 |  |
| 1998 | 8 | 4 | 6 | 17 | 9 | -4.4 | -38.3 | 0 | 2 | 2.3 |  |
| 1998 | 9 | 6 | 13 | 20 | 39 | -4.4 | -38.3 | 0 | 2 | 2.1 |  |
| 1998 | 10 | 7 | 15 | 39 | 13 | -9.9 | -39.9 | 0 | 1 | 2.5 | 2 |
| 1998 | 10 | 18 | 7 | 13 | 13 | -4.4 | -38.3 | 0 | 2 | 2 |  |
| 1998 | 10 | 20 | 23 | 34 | 26 | -4.4 | -38.3 | 0 | 2 | 2.4 |  |
| 1998 | 11 | 19 | 15 | 39 | 59 | -9.7 | -37 | 0 | 10 | 2.5 | 5 |
| 1999 | 1 | 7 | 12 | 45 | 8 | -4.4 | -38.3 | 0 | 2 | 2.1 |  |
| 1999 | 3 | 28 | 3 | 5 | 47 | -9.9 | -39.9 | 0 | 1 | 2.8 |  |
| 1999 | 5 | 5 | 3 | 45 | 53 | -6.6 | -38.4 | 0 | 5 | 2.2 |  |
| 1999 | 5 | 12 | 18 | 59 | 48 | -5.7 | -35.9 | 0 | 5 | 2 |  |
| 1999 | 5 | 31 | 2 | 34 | 10 | -4.4 | -38.2 | 0 | 2 | 2 |  |
| 1999 | 6 | 30 | 20 | 19 | 33 | -5.6 | -35.8 | 0 | 5 | 3 |  |
| 1999 | 6 | 30 | 20 | 24 | 17 | -5.6 | -35.8 | 0 | 5 | 2.1 |  |
| 1999 | 7 | 4 | 14 | 2 | 46 | -5.6 | -35.8 | 0 | 5 | 2.2 |  |
| 1999 | 7 | 11 | 1 | 35 | 0 | -13 | -39.6 | 0 | 10 | 2.9 |  |
| 1999 | 7 | 31 | 20 | 6 | 0 | -4.4 | -38.3 | 0 | 2 | 2.3 |  |
| 1999 | 8 | 13 | 8 | 33 | 32 | -3.9 | -39.5 | 0 | 3 | 2 |  |
| 1999 | 10 | 25 | 9 | 24 | 6 | -9.9 | -39.9 | 0 | 1 | 2.2 |  |
| 1999 | 11 | 17 | 9 | 20 | 0 | -8.3 | -33 | 0 | 5 | 2.4 |  |
| 1999 | 11 | 17 | 12 | 6 | 14 | -8.3 | -36 | 0 | 5 | 3 | 4-5 |
| 1999 | 12 | 22 | 11 | 18 | 55 | -4.4 | -38.3 | 0 | 2 | 2.2 |  |
| 1999 | 12 | 22 | 11 | 19 | 50 | -4.4 | -38.3 | 0 | 2 | 2.8 |  |
| 1999 | 12 | 22 | 12 | 3 | 5 | -4.4 | -38.3 | 0 | 2 | 2.1 |  |
| 1999 | 12 | 23 | 3 | 58 | 2 | -4.4 | -38.3 | 0 | 2 | 2.1 |  |
| 2000 | 1 | 22 | 23 | 36 | 40 | -4.4 | -38.3 | 0 | 2 | 3.3 |  |
| 2000 | 1 | 22 | 23 | 43 | 52 | -4.4 | -38.3 | 0 | 2 | 2.6 |  |
| 2000 | 1 | 23 | 6 | 22 | 39 | -4.4 | -38.3 | 0 | 2 | 3.1 |  |
| 2000 | 1 | 23 | 8 | 4 | 51 | -4.4 | -38.3 | 0 | 2 | 3 |  |
| 2000 | 2 | 25 | 14 | 35 | 27 | -4.4 | -38.3 | 0 | 2 | 2.7 |  |
| 2000 | 5 | 18 | 17 | 53 | 0 | -5.5 | -35.6 | 0 | 30 | 2.4 |  |
| 2000 | 6 | 29 | 2 | 2 | 18 | -4.4 | -38.3 | 0 | 2 | 3.5 |  |
| 2000 | 7 | 4 | 4 | 54 | 47 | -4.4 | -38.3 | 0 | 5 | 4.1 | 4-5 |
| 2000 | 7 | 13 | 23 | 55 | 41 | -9.9 | -39.9 | 0 | 1 | 2.8 |  |
| 2000 | 9 | 23 | 1 | 55 | 25 | -4.3 | -38.4 | 0 | 50 | 3.1 |  |
| 2000 | 10 | 6 | 13 | 4 | 12 | -3.2 | -40.4 | 0 | 5 | 3.2 |  |
| 2000 | 10 | 17 | 13 | 37 | 45 | -3.2 | -40.4 | 0 | 5 | 3 |  |
| 2000 | 11 | 10 | 20 | 5 | 8 | -4.3 | -38.4 | 0 | 50 | 3 |  |
| 2001 | 1 | 23 | 9 | 21 | 31 | -5.3 | -39.4 | 0 | 50 | 3.3 |  |
| 2001 | 2 | 26 | 20 | 42 | 0 | -4.4 | -38.3 | 0 | 2 | 3.7 |  |
| 2001 | 4 | 6 | 10 | 26 | 5 | -9.9 | -39.9 | 0 | 1 | 3 |  |
| 2001 | 12 | 8 | 2 | 6 | 5 | -9.9 | -39.9 | 0 | 1 | 3.1 | 4 |
| 2002 | 5 | 1 | 16 | 21 | 10 | -8.3 | -36 | 0 | 5 | 2.7 |  |
| 2002 | 6 | 30 | 21 | 39 | 31 | -8.3 | -36 | 0 | 5 | 3.8 | 4-5 |
| 2002 | 7 | 14 | 13 | 35 | 0 | -8.3 | -36 | 0 | 5 | 2 |  |
| 2002 | 7 | 17 | 0 | 45 | 0 | -8.3 | -36 | 0 | 5 | 2.5 |  |
| 2002 | 7 | 27 | 12 | 10 | 0 | -8.3 | -36 | 0 | 5 | 2.5 |  |
| 2002 | 7 | 27 | 13 | 23 | 0 | -8.3 | -36 | 0 | 5 | 1.7 |  |
| 2002 | 7 | 27 | 13 | 27 | 0 | -8.3 | -36 | 0 | 5 | 1.9 |  |
| 2002 | 7 | 29 | 13 | 19 | 0 | -8.3 | -36 | 0 | 5 | 2.9 |  |
| 2002 | 8 | 6 | 19 | 53 | 0 | -8.3 | -36 | 0 | 5 | 2.3 |  |
| 2002 | 8 | 7 | 14 | 58 | 0 | -8.3 | -36 | 0 | 5 | 2.5 |  |
| 2002 | 8 | 8 | 0 | 54 | 0 | -8.3 | -36 | 0 | 5 | 2.2 |  |
| 2002 | 8 | 8 | 22 | 22 | 0 | -8.3 | -36 | 0 | 5 | 1.8 |  |
| 2002 | 8 | 9 | 6 | 18 | 0 | -8.3 | -36 | 0 | 5 | 2.2 |  |
| 2002 | 8 | 9 | 14 | 23 | 0 | -8.3 | -36 | 0 | 5 | 1.9 |  |
| 2002 | 8 | 10 | 22 | 16 | 0 | -8.3 | -36 | 0 | 5 | 2.3 |  |
| 2002 | 8 | 11 | 2 | 58 | 0 | -8.3 | -36 | 0 | 5 | 1.7 |  |
| 2002 | 8 | 11 | 22 | 58 | 0 | -8.3 | -36 | 0 | 5 | 2.4 |  |
| 2002 | 8 | 15 | 8 | 34 | 0 | -8.3 | -36 | 0 | 5 | 2.5 |  |
| 2002 | 8 | 16 | 11 | 19 | 0 | -8.3 | -36 | 0 | 5 | 2.8 |  |
| 2002 | 8 | 23 | 13 | 2 | 0 | -8.3 | -36 | 0 | 5 | 2.9 |  |
| 2002 | 8 | 31 | 0 | 0 | 0 | -8.3 | -36 | 0 | 5 | 2.3 |  |
| 2002 | 9 | 1 | 0 | 15 | 0 | -8.3 | -36 | 0 | 5 | 2.4 |  |
| 2002 | 9 | 2 | 3 | 31 | 0 | -8.3 | -36.2 | 0 | 5 | 2.4 |  |
| 2002 | 9 | 20 | 16 | 3 | 1 | -13 | -38.7 | 0 | 20 | 3 |  |
| 2002 | 9 | 28 | 9 | 35 | 8 | -13 | -38.7 | 0 | 20 | 3.7 |  |
| 2002 | 10 | 1 | 17 | 56 | 54 | -13 | -38.7 | 0 | 20 | 2.8 |  |
| 2002 | 10 | 10 | 21 | 9 | 33 | -13 | -38.9 | 0 | 20 | 3.6 |  |
| 2002 | 10 | 17 | 13 | 18 | 8 | -13 | -38.7 | 0 | 20 | 2.9 |  |
| 2003 | 1 | 21 | 7 | 30 | 0 | -8.3 | -36 | 0 | 5 | 2.4 |  |
| 2003 | 1 | 23 | 20 | 31 | 0 | -8.3 | -36 | 0 | 5 | 2.1 |  |
| 2003 | 1 | 25 | 21 | 0 | 0 | -8.3 | -40.2 | 0 | 10 | 3.5 | 5 |
| 2003 | 2 | 18 | 5 | 47 | 0 | -8.3 | -36.2 | 0 | 5 | 2 |  |
| 2003 | 2 | 26 | 14 | 32 | 2 | -5.7 | -35.9 | 0 | 10 | 3.4 |  |
| 2003 | 5 | 20 | 7 | 50 | 31 | -4.2 | -40 | 0 | 30 | 2.5 |  |
| 2003 | 6 | 17 | 2 | 15 | 0 | -8.3 | -36 | 0 | 5 | 2.4 |  |
| 2003 | 7 | 19 | 10 | 32 | 0 | -5.6 | -35.6 | 0 | 5 | 2.7 |  |
| 2003 | 7 | 19 | 22 | 12 | 0 | -5.6 | -35.6 | 0 | 5 | 2.6 |  |
| 2003 | 7 | 20 | 0 | 32 | 0 | -5.6 | -35.6 | 0 | 5 | 2.7 |  |
| 2003 | 7 | 20 | 0 | 43 | 0 | -5.6 | -35.6 | 0 | 5 | 2.1 |  |
| 2003 | 7 | 20 | 9 | 7 | 0 | -5.6 | -35.6 | 0 | 5 | 2.6 |  |
| 2003 | 7 | 20 | 10 | 38 | 0 | -5.6 | -35.6 | 0 | 5 | 1.8 |  |
| 2003 | 7 | 20 | 10 | 43 | 0 | -5.6 | -35.6 | 0 | 5 | 1.9 |  |
| 2003 | 7 | 23 | 0 | 12 | 0 | -5.6 | -35.6 | 0 | 5 | 2.1 |  |
| 2003 | 7 | 23 | 1 | 6 | 0 | -5.6 | -35.6 | 0 | 5 | 2.2 |  |
| 2003 | 7 | 23 | 16 | 8 | 0 | -5.6 | -35.6 | 0 | 5 | 1.7 |  |
| 2003 | 7 | 31 | 11 | 34 | 11 | -14 | -44.6 | 0 | 30 | 2.9 |  |
| 2003 | 7 | 31 | 14 | 43 | 40 | -13 | -44.6 | 0 | 30 | 3 |  |
| 2003 | 7 | 31 | 15 | 29 | 57 | -14 | -44.6 | 0 | 30 | 2.9 |  |
| 2003 | 8 | 22 | 17 | 19 | 0 | -8.3 | -36 | 0 | 5 | 1.5 |  |
| 2003 | 8 | 22 | 18 | 34 | 0 | -8.3 | -36 | 0 | 5 | 1.7 |  |
| 2003 | 8 | 22 | 19 | 34 | 0 | -8.3 | -36 | 0 | 5 | 1.6 |  |
| 2003 | 8 | 22 | 20 | 40 | 0 | -8.3 | -36 | 0 | 5 | 1.7 |  |
| 2003 | 8 | 22 | 21 | 9 | 0 | -8.3 | -36 | 0 | 5 | 1.6 |  |
| 2003 | 12 | 8 | 10 | 21 | 0 | -8.3 | -36 | 0 | 5 | 2.4 |  |
| 2004 | 1 | 11 | 10 | 34 | 20 | -8.3 | -36.6 | 0 | 20 | 2.7 |  |
| 2004 | 1 | 12 | 4 | 51 | 50 | -6.6 | -38.8 | 0 | 50 | 3.2 |  |
| 2004 | 1 | 13 | 8 | 18 | 0 | -8.3 | -36.6 | 0 | 20 | 2.4 |  |
| 2004 | 2 | 3 | 1 | 7 | 0 | -8.3 | -36 | 0 | 5 | 2.5 |  |
| 2004 | 2 | 6 | 5 | 55 | 20 | -5 | -39.7 | 0 | 50 | 2.8 |  |
| 2004 | 3 | 12 | 2 | 50 | 2 | -8.2 | -38.4 | 0 | 0 | 2.2 |  |
| 2004 | 5 | 21 | 11 | 57 | 0 | -8.3 | -36.6 | 0 | 20 | 2.5 |  |
| 2004 | 5 | 21 | 13 | 6 | 0 | -8.3 | -36.6 | 0 | 20 | 2.3 |  |
| 2004 | 5 | 21 | 16 | 29 | 0 | -8.3 | -36.6 | 0 | 20 | 2 |  |
| 2004 | 5 | 22 | 0 | 10 | 0 | -8.3 | -36.6 | 0 | 20 | 2 |  |
| 2004 | 5 | 22 | 0 | 11 | 0 | -8.3 | -36.6 | 0 | 20 | 2.2 |  |
| 2004 | 5 | 22 | 0 | 23 | 0 | -8.3 | -36.6 | 0 | 20 | 2.3 |  |
| 2004 | 5 | 22 | 21 | 40 | 0 | -8.3 | -36.6 | 0 | 20 | 2.3 |  |
| 2004 | 5 | 30 | 17 | 1 | 0 | -8.3 | -36.6 | 0 | 20 | 2.2 |  |
| 2004 | 6 | 1 | 12 | 11 | 0 | -8.3 | -36.6 | 0 | 5 | 3.1 |  |
| 2004 | 6 | 2 | 11 | 45 | 0 | -8.3 | -36.6 | 0 | 5 | 2.3 |  |
| 2004 | 6 | 2 | 11 | 56 | 0 | -8.3 | -36.6 | 0 | 5 | 2 |  |
| 2004 | 6 | 7 | 23 | 25 | 0 | -8.3 | -36.6 | 0 | 5 | 2.5 |  |
| 2004 | 6 | 9 | 2 | 34 | 0 | -8.3 | -36.6 | 0 | 5 | 2.1 |  |
| 2004 | 6 | 9 | 11 | 36 | 0 | -8.3 | -36.6 | 0 | 5 | 2 |  |
| 2004 | 6 | 9 | 12 | 30 | 0 | -8.3 | -36.6 | 0 | 5 | 2.9 |  |
| 2004 | 6 | 10 | 2 | 31 | 0 | -8.3 | -36.6 | 0 | 5 | 2.3 |  |
| 2004 | 6 | 10 | 3 | 20 | 0 | -8.3 | -36.6 | 0 | 5 | 2 |  |
| 2004 | 6 | 14 | 11 | 7 | 0 | -8.3 | -36.6 | 0 | 5 | 2 |  |
| 2004 | 8 | 17 | 1 | 37 | 0 | -8.3 | -36 | 0 | 5 | 1.8 |  |
| 2004 | 8 | 24 | 8 | 14 | 49 | -4.4 | -38.5 | 0 | 10 | 3.3 |  |
| 2004 | 9 | 18 | 8 | 44 | 0 | -5.5 | -35.7 | 0 | 10 | 2.6 |  |
| 2005 | 4 | 3 | 13 | 44 | 59 | -8.8 | -33.2 | 0 | 50 | 3 |  |
| 2005 | 6 | 6 | 5 | 1 | 0 | -4.4 | -38.4 | 0 | 10 | 3 |  |
| 2005 | 6 | 18 | 11 | 10 | 0 | -4.4 | -38.4 | 0 | 10 | 3.1 |  |
| 2005 | 6 | 27 | 14 | 37 | 0 | -5.7 | -37.9 | 0 | 5 | 2.3 |  |
| 2005 | 7 | 23 | 0 | 0 | 0 | -5.7 | -37.9 | 0 | 5 | 2 |  |
| 2005 | 7 | 24 | 0 | 0 | 0 | -5.7 | -37.9 | 0 | 5 | 1.5 |  |
| 2005 | 8 | 26 | 13 | 57 | 0 | -5.9 | -37.3 | 0 | 10 | 2 |  |
| 2005 | 12 | 14 | 11 | 40 | 0 | -5.6 | -35.6 | 0 | 10 | 2 |  |
| 2006 | 1 | 8 | 1 | 54 | 55 | -10 | -36.9 | 0 | 10 | 3.4 | 5 |
| 2006 | 1 | 16 | 0 | 0 | 0 | -7.3 | -35.3 | 0 | 10 | 2.6 |  |
| 2006 | 3 | 29 | 0 | 0 | 0 | -14 | -40.1 | 0 | 10 | 3.7 | 5-6 |
| 2006 | 5 | 18 | 23 | 41 | 0 | -8.3 | -36.2 | 0 | 5 | 2.5 |  |
| 2006 | 5 | 20 | 4 | 26 | 6 | -8.3 | -36.2 | 6 | 4 | 3.9 | 5-6 |
| 2006 | 6 | 14 | 7 | 30 | 0 | -4.4 | -38.3 | 0 | 2 | 2.6 |  |
| 2006 | 6 | 28 | 8 | 0 | 0 | -4.4 | -38.3 | 0 | 2 | 3.1 |  |
| 2006 | 7 | 20 | 17 | 20 | 0 | -8.3 | -36.2 | 0 | 5 | 3.3 |  |
| 2006 | 8 | 30 | 0 | 0 | 0 | -11 | -37.1 | 0 | 10 | 2.5 |  |
| 2006 | 9 | 1 | 13 | 55 | 0 | -8.3 | -36.2 | 0 | 5 | 3.6 |  |
| 2006 | 9 | 2 | 23 | 56 | 0 | -5 | -35 | 0 | 30 | 4 |  |
| 2007 | 3 | 20 | 13 | 38 | 53 | -8.3 | -36.2 | 5 | 1 | 3.9 |  |
| 2007 | 3 | 20 | 14 | 22 | 0 | -8.3 | -36.2 | 0 | 5 | 3.4 |  |
| 2007 | 3 | 23 | 4 | 41 | 0 | -8.3 | -36.2 | 0 | 5 | 2.6 |  |
| 2007 | 3 | 26 | 0 | 44 | 17 | -8.3 | -36.2 | 0 | 5 | 3.3 |  |
| 2007 | 3 | 28 | 15 | 4 | 0 | -8.3 | -36.6 | 0 | 30 | 2.8 |  |
| 2007 | 4 | 15 | 2 | 0 | 0 | -3.2 | -40.1 | 0 | 30 | 2.9 |  |
| 2007 | 6 | 25 | 2 | 26 | 38 | -5.3 | -38.3 | 0 | 5 | 2.1 |  |
| 2007 | 7 | 10 | 9 | 29 | 25 | -5.3 | -38.3 | 0 | 5 | 2.3 |  |
| 2007 | 9 | 13 | 10 | 40 | 2 | -5.9 | -38.1 | 5 | 2 | 3.2 | 5 |
| 2007 | 10 | 25 | 22 | 30 | 0 | -11 | -41.7 | 0 | 20 | 3 | 4 |
| 2008 | 1 | 28 | 15 | 46 | 0 | -3.6 | -40.5 | 0 | 5 | 2.5 | 3 |
| 2008 | 2 | 2 | 22 | 36 | 0 | -3.6 | -40.5 | 0 | 5 | 2.8 |  |
| 2008 | 2 | 15 | 18 | 0 | 0 | -6.1 | -39.9 | 0 | 5 | 2.3 | 2-3 |
| 2008 | 2 | 17 | 1 | 57 | 0 | -3.6 | -40.5 | 0 | 5 | 3.7 | 4 |
| 2008 | 2 | 29 | 4 | 48 | 0 | -3.6 | -40.5 | 0 | 5 | 3.7 |  |
| 2008 | 3 | 1 | 0 | 58 | 0 | -3.6 | -40.5 | 0 | 5 | 3.4 |  |
| 2008 | 3 | 4 | 11 | 45 | 0 | -3.6 | -40.5 | 0 | 5 | 2.4 |  |
| 2008 | 3 | 5 | 20 | 3 | 0 | -3.6 | -40.5 | 0 | 5 | 2.8 |  |
| 2008 | 3 | 21 | 6 | 20 | 0 | -3.6 | -40.5 | 0 | 5 | 2.9 |  |
| 2008 | 3 | 21 | 11 | 16 | 0 | -3.6 | -40.5 | 0 | 5 | 2.2 |  |
| 2008 | 4 | 4 | 17 | 7 | 0 | -3.6 | -40.5 | 0 | 5 | 3.5 |  |
| 2008 | 5 | 1 | 8 | 36 | 0 | -3.6 | -40.5 | 0 | 5 | 3.1 |  |
| 2008 | 5 | 21 | 19 | 23 | 59 | -3.6 | -40.5 | 5 | 5 | 3.8 |  |
| 2008 | 5 | 21 | 19 | 28 | 16 | -3.6 | -40.5 | 0 | 5 | 3.9 |  |
| 2008 | 5 | 22 | 21 | 1 | 0 | -3.6 | -40.5 | 0 | 5 | 3 |  |
| 2008 | 7 | 31 | 3 | 12 | 6 | -14 | -44.7 | 0 | 45 | 2.9 |  |
| 2008 | 9 | 11 | 12 | 35 | 27 | -3.6 | -40.5 | 0 | 5 | 2.4 |  |
| 2008 | 9 | 15 | 17 | 14 | 43 | -3.6 | -40.5 | 0 | 5 | 2.4 |  |
| 2008 | 9 | 19 | 11 | 39 | 50 | -3.6 | -40.5 | 0 | 5 | 2.1 |  |
| 2008 | 10 | 12 | 19 | 29 | 41 | -3.6 | -40.5 | 0 | 5 | 2.4 |  |
| 2008 | 10 | 26 | 19 | 9 | 42 | -3.6 | -40.5 | 0 | 5 | 2.2 |  |
| 2008 | 12 | 19 | 0 | 0 | 0 | -3.6 | -40.5 | 0 | 5 | 3.5 |  |
| 2009 | 1 | 31 | 2 | 50 | 59 | -14 | -44.7 | 11 | 30 | 2.6 |  |
| 2009 | 2 | 9 | 14 | 45 | 0 | -3.6 | -40.5 | 0 | 5 | 3.5 |  |
| 2009 | 3 | 18 | 16 | 50 | 8 | -3.6 | -40.5 | 5 | 5 | 2.5 |  |
| 2009 | 3 | 20 | 0 | 0 | 0 | -3.6 | -40.5 | 5 | 5 | 2.2 |  |
| 2009 | 3 | 22 | 23 | 55 | 0 | -3.6 | -40.5 | 5 | 5 | 2.5 |  |
| 2009 | 4 | 17 | 8 | 55 | 33 | -3.6 | -40.5 | 5 | 10 | 2.6 |  |
| 2009 | 5 | 27 | 19 | 19 | 51 | -3.6 | -40.5 | 5 | 10 | 2.2 |  |
| 2009 | 5 | 27 | 23 | 30 | 34 | -3.6 | -40.5 | 5 | 10 | 2.3 |  |
| 2009 | 6 | 11 | 6 | 47 | 42 | -4.2 | -38.7 | 5 | 10 | 2.6 |  |
| 2009 | 7 | 4 | 9 | 47 | 0 | -3.5 | -40.2 | 0 | 10 | 1.3 |  |
| 2009 | 7 | 8 | 16 | 57 | 43 | -3.6 | -40.5 | 5 | 5 | 2.4 |  |
| 2009 | 7 | 8 | 17 | 5 | 0 | -3.6 | -40.5 | 5 | 5 | 2.6 |  |
| 2009 | 7 | 19 | 19 | 46 | 0 | -3.6 | -40.5 | 5 | 5 | 2.2 |  |
| 2009 | 7 | 31 | 14 | 28 | 57 | -3.6 | -40.5 | 5 | 10 | 2.5 |  |
| 2009 | 7 | 31 | 20 | 55 | 21 | -3.6 | -40.5 | 5 | 10 | 3.4 |  |
| 2009 | 9 | 7 | 22 | 14 | 17 | -3.1 | -40.7 | 5 | 30 | 2.6 |  |
| 2009 | 9 | 8 | 7 | 16 | 59 | -4.6 | -38.2 | 0 | 5 | 2.6 | 3 |
| 2009 | 10 | 27 | 0 | 45 | 27 | -3.8 | -40.3 | 5 | 10 | 2.6 | 4 |
| 2009 | 11 | 25 | 9 | 43 | 0 | -8.3 | -36 | 0 | 5 | 2.1 |  |
| 2009 | 12 | 21 | 8 | 25 | 0 | -3.6 | -40.7 | 0 | 10 | 2.9 |  |
| 2010 | 1 | 2 | 3 | 28 | 42 | -3.6 | -40.7 | 5 | 10 | 2.7 |  |
| 2010 | 1 | 9 | 18 | 18 | 39 | -5.6 | -35.7 | 10 | 5 | 3.5 |  |
| 2010 | 1 | 11 | 15 | 54 | 30 | -5.6 | -35.7 | 10 | 5 | 4.3 |  |
| 2010 | 2 | 28 | 2 | 21 | 0 | -8.3 | -36.2 | 0 | 5 | 2.9 |  |
| 2010 | 3 | 3 | 20 | 41 | 0 | -8.5 | -36.8 | 0 | 5 | 1.6 |  |
| 2010 | 3 | 3 | 21 | 0 | 0 | -5.9 | -38.1 | 5 | 2 | 2 |  |
| 2010 | 3 | 3 | 22 | 49 | 0 | -8.5 | -36.8 | 0 | 5 | 2 |  |
| 2010 | 3 | 3 | 23 | 17 | 0 | -8.5 | -36.8 | 0 | 5 | 1.8 |  |
| 2010 | 3 | 8 | 23 | 14 | 0 | -8.5 | -36.8 | 0 | 5 | 3.2 |  |
| 2010 | 3 | 11 | 2 | 57 | 9 | -8.3 | -36.2 | 0 | 5 | 2.7 |  |
| 2010 | 3 | 25 | 10 | 39 | 0 | -6 | -36.8 | 0 | 5 | 2.9 |  |
| 2010 | 3 | 28 | 16 | 41 | 0 | -5.6 | -35.7 | 0 | 5 | 2.3 |  |
| 2010 | 4 | 7 | 21 | 19 | 0 | -8.5 | -36.8 | 0 | 5 | 2.6 |  |
| 2010 | 4 | 9 | 7 | 59 | 0 | -3.9 | -39.9 | 0 | 5 | 2.3 |  |
| 2010 | 4 | 17 | 7 | 6 | 0 | -8.3 | -36.2 | 0 | 5 | 2.3 |  |
| 2010 | 4 | 18 | 23 | 41 | 0 | -8.6 | -35.9 | 0 | 5 | 2.8 |  |
| 2010 | 4 | 19 | 13 | 22 | 0 | -8.6 | -35.9 | 0 | 5 | 2 |  |
| 2010 | 4 | 19 | 13 | 32 | 0 | -8.6 | -35.9 | 0 | 5 | 1.9 |  |
| 2010 | 4 | 20 | 21 | 9 | 0 | -8.6 | -35.9 | 0 | 5 | 2.2 |  |
| 2010 | 4 | 26 | 10 | 33 | 56 | -3.5 | -44.8 | 0 | 10 | 2.6 |  |
| 2010 | 6 | 1 | 6 | 58 | 47 | -13 | -39.5 | 0 | 10 | 3.2 |  |
| 2010 | 6 | 11 | 9 | 58 | 0 | -8.3 | -36.3 | 0 | 10 | 2.7 |  |
| 2010 | 6 | 14 | 5 | 0 | 0 | -11 | -41.3 | 0 | 10 | 2.5 | 4-5 |
| 2010 | 7 | 19 | 4 | 10 | 0 | -8.3 | -36.2 | 0 | 5 | 2.7 |  |
| 2010 | 9 | 1 | 7 | 11 | 0 | -8.3 | -36.2 | 0 | 5 | 2 |  |
| 2010 | 10 | 26 | 13 | 15 | 0 | -3.6 | -40.5 | 5 | 5 | 2.6 |  |
| 2010 | 10 | 31 | 0 | 0 | 0 | -3.6 | -40.5 | 5 | 5 | 3.1 |  |
| 2010 | 12 | 4 | 11 | 26 | 0 | -5.5 | -36.1 | 5 | 3 | 3.1 |  |
| 2010 | 12 | 4 | 23 | 58 | 0 | -5.5 | -36.1 | 5 | 3 | 3 |  |
| 2010 | 12 | 5 | 0 | 1 | 0 | -5.5 | -36.1 | 5 | 3 | 3.1 |  |
| 2010 | 12 | 5 | 0 | 25 | 0 | -5.5 | -36.1 | 5 | 3 | 2 |  |
| 2010 | 12 | 6 | 9 | 27 | 0 | -5.5 | -36.1 | 5 | 3 | 1.8 |  |
| 2010 | 12 | 8 | 0 | 12 | 0 | -5.5 | -36.1 | 5 | 3 | 2.3 |  |
| 2010 | 12 | 13 | 3 | 16 | 0 | -5.5 | -36.1 | 5 | 3 | 2.1 |  |
| 2010 | 12 | 14 | 10 | 46 | 0 | -5.5 | -36.1 | 5 | 3 | 2.5 |  |
| 2010 | 12 | 31 | 3 | 1 | 0 | -5.5 | -36.1 | 5 | 3 | 2.6 |  |
| 2011 | 2 | 14 | 18 | 58 | 0 | -5.6 | -35.7 | 0 | 5 | 2.1 |  |
| 2011 | 3 | 12 | 15 | 48 | 0 | -5.6 | -35.7 | 0 | 5 | 2.6 |  |
| 2011 | 3 | 16 | 20 | 8 | 0 | -8.3 | -36 | 0 | 5 | 2 |  |
| 2011 | 3 | 17 | 15 | 26 | 0 | -8.3 | -36 | 0 | 5 | 1.9 |  |
| 2011 | 4 | 4 | 15 | 20 | 0 | -3.5 | -40.3 | 0 | 10 | 2.7 |  |
| 2011 | 4 | 19 | 1 | 19 | 0 | -3.6 | -40.5 | 0 | 5 | 2.7 |  |
| 2011 | 4 | 23 | 2 | 58 | 0 | -3.5 | -40.3 | 0 | 10 | 2.7 |  |
| 2011 | 6 | 7 | 2 | 17 | 0 | -8.2 | -35.8 | 0 | 10 | 2.2 |  |
| 2011 | 7 | 8 | 10 | 46 | 0 | -6.1 | -36.8 | 0 | 5 | 2.1 |  |
| 2011 | 7 | 19 | 5 | 52 | 0 | -6 | -35.8 | 0 | 5 | 1 |  |
| 2011 | 8 | 8 | 13 | 53 | 0 | -3.6 | -40.5 | 5 | 5 | 3 |  |
| 2011 | 10 | 12 | 17 | 22 | 0 | -5.5 | -35.8 | 0 | 5 | 1.9 |  |
| 2011 | 10 | 12 | 17 | 31 | 9 | -5.5 | -35.8 | 0 | 5 | 2.3 |  |
| 2011 | 10 | 19 | 11 | 48 | 0 | -5.5 | -35.8 | 0 | 5 | 2.1 |  |
| 2011 | 10 | 21 | 23 | 12 | 0 | -5.5 | -35.8 | 0 | 5 | 2.3 |  |
| 2011 | 10 | 22 | 0 | 26 | 0 | -5.5 | -35.8 | 0 | 5 | 2.2 |  |
| 2011 | 10 | 22 | 0 | 39 | 0 | -5.5 | -35.8 | 0 | 5 | 2.1 |  |
| 2011 | 10 | 23 | 10 | 17 | 0 | -5.5 | -35.8 | 0 | 5 | 2.6 |  |
| 2011 | 10 | 23 | 10 | 29 | 0 | -5.5 | -35.8 | 0 | 5 | 2.2 |  |
| 2011 | 10 | 24 | 12 | 38 | 0 | -5.5 | -35.8 | 0 | 5 | 2.8 |  |
| 2011 | 11 | 3 | 5 | 19 | 0 | -3.6 | -40.5 | 5 | 5 | 1.8 |  |
| 2011 | 11 | 9 | 5 | 18 | 0 | -3.5 | -40.2 | 0 | 10 | 2.1 |  |
| 2011 | 11 | 9 | 9 | 33 | 0 | -3.5 | -40.2 | 0 | 10 | 1.9 |  |
| 2011 | 11 | 23 | 19 | 33 | 0 | -5.5 | -36.1 | 5 | 3 | 2 |  |
| 2011 | 12 | 3 | 15 | 49 | 0 | -9 | -40.5 | 0 | 20 | 3.3 | 4-5 |
| 2011 | 12 | 5 | 15 | 50 | 0 | -5.5 | -35.7 | 0 | 10 | 1.8 |  |
| 2011 | 12 | 11 | 16 | 53 | 0 | -5.5 | -36.1 | 5 | 3 | 1.8 |  |
| 2011 | 12 | 11 | 16 | 55 | 0 | -5.5 | -36.1 | 5 | 3 | 2 |  |
| 2011 | 12 | 15 | 5 | 19 | 0 | -3.6 | -40.5 | 5 | 5 | 2 |  |
| 2011 | 12 | 16 | 3 | 22 | 0 | -5.5 | -36.1 | 0 | 3 | 2.1 |  |
| 2011 | 12 | 17 | 16 | 53 | 0 | -5.5 | -36.1 | 0 | 3 | 1.8 |  |
| 2011 | 12 | 17 | 16 | 55 | 0 | -5.5 | -36.1 | 0 | 3 | 2 |  |
| 2012 | 1 | 24 | 4 | 41 | 0 | -3.6 | -40.5 | 5 | 5 | 2.1 |  |
| 2012 | 2 | 17 | 7 | 11 | 0 | -3 | -41.1 | 0 | 20 | 2.5 |  |
| 2012 | 2 | 26 | 3 | 8 | 0 | -5.5 | -35.7 | 0 | 10 | 1.5 |  |
| 2012 | 3 | 10 | 18 | 26 | 0 | -3.4 | -34 | 0 | 50 | 2.3 |  |
| 2012 | 3 | 18 | 3 | 14 | 0 | -8.2 | -36 | 0 | 5 | 2.1 |  |
| 2012 | 3 | 28 | 14 | 15 | 0 | -8.3 | -36.2 | 0 | 5 | 2.6 |  |
| 2012 | 3 | 28 | 23 | 53 | 0 | -8.3 | -36.2 | 0 | 5 | 2.6 |  |
| 2012 | 3 | 29 | 0 | 22 | 0 | -8.3 | -36.2 | 0 | 5 | 1.8 |  |
| 2012 | 3 | 29 | 0 | 24 | 0 | -8.3 | -36.2 | 0 | 5 | 1.8 |  |
| 2012 | 3 | 29 | 17 | 15 | 0 | -8.3 | -36.2 | 0 | 5 | 3.1 |  |
| 2012 | 4 | 15 | 18 | 4 | 0 | -5.5 | -36.1 | 0 | 5 | 2.8 |  |
| 2012 | 4 | 20 | 19 | 33 | 0 | -8.3 | -36.2 | 0 | 5 | 2.2 |  |
| 2012 | 4 | 22 | 0 | 0 | 0 | -11 | -41.8 | 0 | 50 | 2.8 |  |
| 2012 | 4 | 26 | 8 | 46 | 0 | -4.3 | -37.9 | 0 | 20 | 2 |  |
| 2012 | 4 | 26 | 23 | 54 | 0 | -5.5 | -36.1 | 0 | 5 | 2.4 |  |
| 2012 | 5 | 1 | 8 | 48 | 0 | -6 | -37.9 | 0 | 5 | 2.7 |  |
| 2012 | 5 | 2 | 13 | 51 | 0 | -4 | -39.1 | 0 | 10 | 2.1 |  |
| 2012 | 5 | 8 | 4 | 9 | 0 | -8.3 | -36.2 | 0 | 5 | 2.2 |  |
| 2012 | 6 | 13 | 10 | 14 | 0 | -5.4 | -35.7 | 0 | 5 | 1.6 |  |
| 2012 | 6 | 18 | 2 | 50 | 0 | -5.9 | -36.9 | 0 | 10 | 1.3 |  |
| 2012 | 6 | 20 | 13 | 52 | 0 | -5.5 | -36.1 | 0 | 3 | 2.7 |  |
| 2012 | 7 | 17 | 22 | 32 | 0 | -5.9 | -36.9 | 0 | 5 | 1.7 |  |
| 2012 | 8 | 2 | 7 | 14 | 0 | -3.6 | -40.5 | 0 | 5 | 2.9 |  |
| 2012 | 8 | 2 | 7 | 41 | 0 | -3.6 | -40.5 | 0 | 5 | 2.2 |  |
| 2012 | 8 | 7 | 16 | 9 | 0 | -8.3 | -36.2 | 0 | 5 | 2.2 |  |
| 2012 | 8 | 7 | 20 | 27 | 0 | -8.3 | -36.2 | 0 | 5 | 2.2 |  |
| 2012 | 9 | 11 | 23 | 5 | 0 | -5.5 | -36.1 | 0 | 5 | 2.7 |  |
| 2012 | 9 | 12 | 18 | 6 | 0 | -5.5 | -36.1 | 0 | 5 | 3 |  |
| 2012 | 10 | 3 | 4 | 52 | 0 | -8.3 | -36.2 | 0 | 5 | 2.7 |  |
| 2012 | 10 | 4 | 16 | 38 | 0 | -5.5 | -36.1 | 0 | 5 | 1.8 |  |
| 2012 | 10 | 6 | 22 | 52 | 0 | -5.5 | -36.1 | 0 | 5 | 2.8 |  |
| 2012 | 10 | 10 | 8 | 15 | 0 | -5.5 | -36.1 | 0 | 5 | 1.8 |  |
| 2012 | 10 | 10 | 16 | 16 | 0 | -8.4 | -35.8 | 0 | 5 | 2.3 |  |
| 2012 | 10 | 11 | 10 | 8 | 0 | -5.5 | -36.1 | 0 | 5 | 2 |  |
| 2012 | 10 | 16 | 8 | 44 | 0 | -5.5 | -36.1 | 0 | 5 | 1.8 |  |
| 2012 | 10 | 23 | 20 | 28 | 0 | -5.5 | -36.1 | 0 | 5 | 1.5 |  |
| 2012 | 11 | 7 | 9 | 13 | 0 | -5.5 | -36.1 | 0 | 5 | 2.1 |  |
| 2012 | 11 | 17 | 0 | 41 | 0 | -5.5 | -35.8 | 0 | 5 | 2.8 |  |
| 2012 | 11 | 18 | 13 | 28 | 0 | -5.5 | -36.1 | 0 | 5 | 2.6 |  |
| 2012 | 11 | 21 | 12 | 59 | 0 | -5.5 | -36.1 | 0 | 5 | 1.6 |  |
| 2012 | 11 | 25 | 20 | 41 | 0 | -5.5 | -36.1 | 0 | 5 | 1.6 |  |
| 2012 | 12 | 6 | 11 | 16 | 0 | -5.5 | -36.1 | 0 | 5 | 2.4 |  |
| 2012 | 12 | 15 | 3 | 1 | 0 | -5.5 | -36.1 | 0 | 5 | 2.4 |  |
| 2012 | 12 | 21 | 12 | 20 | 0 | -5.5 | -36.1 | 0 | 5 | 1.9 |  |
| 2012 | 12 | 21 | 12 | 37 | 0 | -5.5 | -36.1 | 0 | 5 | 3.6 |  |
| 2012 | 12 | 21 | 13 | 7 | 0 | -5.5 | -36.1 | 0 | 5 | 1.7 |  |
| 2012 | 12 | 22 | 12 | 8 | 0 | -5.3 | -35.9 | 0 | 5 | 1.7 |  |
| 2012 | 12 | 22 | 15 | 38 | 0 | -5.3 | -35.9 | 0 | 5 | 1.9 |  |
| 2012 | 12 | 24 | 11 | 37 | 0 | -5.5 | -36.1 | 0 | 5 | 2.7 |  |
| 2012 | 12 | 24 | 16 | 16 | 0 | -5.2 | -35.9 | 0 | 5 | 2.1 |  |
| 2012 | 12 | 26 | 7 | 30 | 0 | -5.2 | -35.9 | 0 | 5 | 2.2 |  |
| 2012 | 12 | 28 | 2 | 25 | 12 | -5.2 | -35.9 | 0 | 5 | 1.7 |  |
| 2012 | 12 | 28 | 2 | 25 | 32 | -5.2 | -35.9 | 0 | 5 | 2.6 |  |
| 2012 | 12 | 28 | 5 | 14 | 0 | -5.2 | -35.9 | 0 | 5 | 1.7 |  |
| 2012 | 12 | 28 | 10 | 57 | 0 | -5.2 | -35.9 | 0 | 5 | 1.5 |  |
| 2013 | 1 | 5 | 12 | 18 | 45 | -5.5 | -36.1 | 0 | 10 | 3.3 |  |
| 2013 | 1 | 5 | 13 | 1 | 47 | -5.5 | -36.1 | 0 | 10 | 2.7 |  |
| 2013 | 1 | 12 | 7 | 10 | 43 | -5.5 | -36.1 | 0 | 10 | 1.9 |  |
| 2013 | 1 | 25 | 19 | 30 | 37 | -6.1 | -36.3 | 0 | 2 | 2.4 |  |
| 2013 | 3 | 3 | 13 | 36 | 31 | -5.4 | -36.1 | 0 | 10 | 2.3 |  |
| 2013 | 3 | 17 | 20 | 28 | 29 | -3.7 | -40.5 | 0 | 12 | 2.6 |  |
| 2013 | 10 | 24 | 22 | 34 | 41 | -5.4 | -36.1 | 0 | 10 | 2.7 |  |
| 2013 | 10 | 25 | 11 | 9 | 39 | -5.4 | -36.1 | 2 | 10 | 3.7 |  |
| 2013 | 10 | 28 | 14 | 22 | 40 | -5.4 | -36.1 | 0 | 10 | 2.2 |  |
| 2013 | 10 | 29 | 15 | 17 | 29 | -5.4 | -36.1 | 0 | 10 | 3.4 |  |
| 2013 | 10 | 30 | 17 | 58 | 56 | -5.5 | -36.1 | 0 | 10 | 2.1 |  |
| 2013 | 10 | 31 | 22 | 56 | 10 | -5.3 | -36.1 | 0 | 10 | 2.6 |  |
| 2013 | 11 | 1 | 1 | 5 | 14 | -5.4 | -36.1 | 2 | 10 | 2 |  |
| 2013 | 11 | 1 | 11 | 51 | 17 | -5.4 | -36.1 | 2 | 10 | 2.8 |  |
| 2013 | 11 | 1 | 22 | 7 | 49 | -5.5 | -36.1 | 2 | 10 | 2.5 |  |
| 2013 | 11 | 2 | 10 | 25 | 34 | -5.4 | -36.1 | 2 | 10 | 2.1 |  |
| 2013 | 11 | 2 | 11 | 28 | 51 | -5.6 | -36.2 | 2 | 10 | 1.8 |  |
| 2013 | 11 | 2 | 14 | 34 | 6 | -5.5 | -36.1 | 2 | 10 | 1.5 |  |
| 2013 | 11 | 2 | 19 | 29 | 42 | -5.5 | -36.1 | 2 | 10 | 1.9 |  |
| 2013 | 11 | 3 | 11 | 39 | 56 | -5.5 | -36.1 | 2 | 10 | 2.4 |  |
| 2013 | 11 | 3 | 11 | 41 | 57 | -5.5 | -36.1 | 2 | 10 | 2.1 |  |
| 2013 | 11 | 5 | 4 | 6 | 32 | -5.5 | -36.1 | 2 | 10 | 2 |  |
| 2013 | 11 | 5 | 5 | 28 | 6 | -5.4 | -36.1 | 2 | 10 | 2 |  |
| 2013 | 11 | 5 | 16 | 38 | 13 | -5.5 | -36.2 | 2 | 10 | 3 |  |
| 2013 | 11 | 15 | 7 | 42 | 34 | -5.5 | -36.1 | 2 | 10 | 3 |  |
| 2013 | 11 | 25 | 13 | 4 | 11 | -5.5 | -36.1 | 0 | 20 | 2.8 |  |
| 2013 | 11 | 27 | 5 | 50 | 5 | -5.4 | -35.8 | 0 | 20 | 3.5 |  |
| 2013 | 12 | 1 | 22 | 26 | 56 | -5.6 | -36.2 | 0 | 20 | 1.7 |  |
| 2013 | 12 | 2 | 6 | 16 | 40 | -5.4 | -36.1 | 0 | 20 | 3.2 |  |
| 2013 | 12 | 4 | 19 | 12 | 28 | -5.5 | -36.1 | 0 | 20 | 2.1 |  |
| 2013 | 12 | 6 | 15 | 41 | 36 | -5.5 | -36.1 | 0 | 20 | 2.4 |  |
| 2013 | 12 | 26 | 16 | 25 | 23 | -5.6 | -36.2 | 0 | 20 | 2.1 |  |
| 2014 | 1 | 5 | 20 | 32 | 10 | -5.4 | -36.1 | 2 | 16 | 1.3 |  |
| 2014 | 1 | 7 | 12 | 14 | 0 | -5.5 | -36.2 | 0 | 10 | 2.6 |  |
| 2014 | 3 | 10 | 12 | 38 | 20 | -13 | -41.7 | 0 | 30 | 2 |  |
| 2014 | 4 | 20 | 16 | 9 | 44 | -3 | -40.6 | 0 | 10 | 2.3 |  |
| 2014 | 4 | 27 | 12 | 45 | 9 | -5.9 | -35.6 | 2 | 10 | 1.3 |  |
| 2014 | 4 | 27 | 12 | 51 | 39 | -5.9 | -36.6 | 2 | 15 | 1.7 |  |
| 2014 | 6 | 16 | 21 | 42 | 6 | -6.5 | -36.9 | 0 | 5 | 2.2 |  |
| 2014 | 6 | 18 | 3 | 29 | 25 | -5.6 | -36.1 | 0 | 1 | 3.5 |  |
| 2014 | 6 | 22 | 15 | 26 | 57 | -5.6 | -36.1 | 0 | 5 | 2.3 |  |
| 2014 | 7 | 30 | 20 | 1 | 37 | -13 | -44.5 | 0 | 20 | 2.2 |  |
| 2014 | 8 | 13 | 20 | 34 | 58 | -13 | -44.6 | 0 | 10 | 2.5 |  |
| 2014 | 10 | 22 | 6 | 9 | 8 | -8.6 | -43.4 | 0 | 45 | 3.3 |  |
| 2014 | 11 | 16 | 5 | 17 | 23 | -3.5 | -40.7 | 0 | 5 | 3.6 | 4-5 |
| 2014 | 11 | 29 | 15 | 31 | 46 | -14 | -43 | 0 | 30 | 2.2 |  |
| 2014 | 12 | 29 | 17 | 39 | 38 | -5.5 | -36.1 | 0 | 10 | 1.5 |  |
| 2015 | 1 | 15 | 18 | 20 | 21 | -13 | -39 | 0 | 30 | 3.1 |  |
| 2015 | 2 | 3 | 14 | 51 | 0 | -8.3 | -36 | 0 | 1 | 1.9 |  |
| 2015 | 2 | 3 | 15 | 30 | 35 | -8.4 | -36 | 0 | 10 | 0.7 |  |
| 2015 | 2 | 3 | 19 | 38 | 15 | -8.3 | -36 | 2 | 2 | 2.4 |  |
| 2015 | 2 | 5 | 0 | 53 | 27 | -8.4 | -36 | 0 | 10 | 2 |  |
| 2015 | 2 | 5 | 14 | 29 | 19 | -6 | -36.9 | 0 | 20 | 1.2 |  |
| 2015 | 2 | 6 | 11 | 24 | 6 | -8.3 | -36 | 0 | 2 | 1.5 |  |
| 2015 | 2 | 6 | 22 | 10 | 5 | -8.3 | -36 | 0 | 2 | 1.8 |  |
| 2015 | 2 | 7 | 8 | 23 | 21 | -8.3 | -36 | 0 | 2 | 0.9 |  |
| 2015 | 2 | 7 | 17 | 1 | 22 | -3.6 | -40.5 | 0 | 1 | 2 |  |
| 2015 | 2 | 19 | 1 | 49 | 51 | -9.8 | -36.7 | 0 | 1 | 2.3 |  |
| 2015 | 3 | 10 | 0 | 22 | 25 | -5.5 | -35.8 | 0 | 10 | 2.1 |  |
| 2015 | 3 | 11 | 20 | 1 | 9 | -9.5 | -35.8 | 0 | 10 | 1.3 |  |
| 2015 | 3 | 14 | 15 | 44 | 17 | -7.3 | -36.8 | 0 | 10 | 1.5 |  |
| 2015 | 3 | 16 | 20 | 1 | 8 | -9.6 | -36 | 0 | 10 | 1.8 |  |
| 2015 | 3 | 16 | 20 | 2 | 39 | -8.2 | -35 | 0 | 15 | 1.3 |  |
| 2015 | 3 | 17 | 19 | 7 | 4 | -14 | -39.2 | 0 | 20 | 2.3 |  |
| 2015 | 3 | 18 | 14 | 31 | 52 | -4 | -38.6 | 0 | 5 | 1.7 |  |
| 2015 | 3 | 19 | 18 | 51 | 43 | -7.7 | -36.4 | 0 | 10 | 1.8 |  |
| 2015 | 3 | 26 | 2 | 24 | 56 | -4.2 | -38.1 | 0 | 50 | 2.5 |  |
| 2015 | 4 | 15 | 12 | 18 | 38 | -5.4 | -39.2 | 0 | 20 | 2.2 |  |
| 2015 | 4 | 18 | 0 | 46 | 20 | -4.9 | -37.6 | 0 | 20 | 2.1 |  |
| 2015 | 5 | 5 | 12 | 11 | 24 | -3.6 | -40.5 | 0 | 10 | 2.5 |  |
| 2015 | 5 | 13 | 11 | 24 | 33 | -3.6 | -40.5 | 0 | 1 | 3 |  |
| 2015 | 6 | 5 | 1 | 29 | 42 | -4.6 | -38.3 | 0 | 40 | 2.7 |  |
| 2015 | 6 | 16 | 4 | 17 | 4 | -5.4 | -36.1 | 0 | 18 | 1.8 |  |
| 2015 | 6 | 30 | 19 | 20 | 3 | -8.3 | -35.9 | 0 | 5 | 2 |  |
| 2015 | 6 | 30 | 19 | 49 | 54 | -8.3 | -35.9 | 0 | 5 | 2.2 |  |
| 2015 | 7 | 20 | 22 | 0 | 42 | -3.1 | -40.7 | 0 | 15 | 3.2 |  |
| 2015 | 7 | 25 | 21 | 42 | 50 | -8.3 | -35.9 | 0 | 8 | 2.5 |  |
| 2015 | 7 | 26 | 0 | 24 | 40 | -3 | -40.7 | 0 | 20 | 1.8 |  |
| 2015 | 7 | 29 | 10 | 8 | 42 | -3.3 | -40.4 | 0 | 10 | 2.1 |  |
| 2015 | 9 | 10 | 23 | 28 | 53 | -3.8 | -39.9 | 0 | 15 | 2.3 |  |
| 2015 | 9 | 10 | 23 | 33 | 0 | -3.8 | -39.9 | 0 | 15 | 1.3 |  |
| 2015 | 9 | 14 | 1 | 29 | 38 | -3.8 | -39.8 | 0 | 10 | 1.7 |  |
| 2015 | 9 | 15 | 21 | 38 | 23 | -3.8 | -39.9 | 0 | 20 | 2.5 |  |
| 2015 | 9 | 20 | 11 | 49 | 19 | -5.5 | -35.8 | 0 | 10 | 3.1 |  |
| 2015 | 9 | 23 | 4 | 30 | 56 | -5.5 | -35.7 | 0 | 10 | 2.1 |  |
| 2015 | 9 | 23 | 12 | 49 | 2 | -5.5 | -35.8 | 0 | 10 | 2.5 |  |
| 2015 | 9 | 27 | 7 | 15 | 10 | -5.6 | -46.7 | 0 | 6 | 2.3 |  |
| 2015 | 10 | 8 | 16 | 58 | 31 | -3.8 | -39.9 | 0 | 15 | 2.5 |  |
| 2015 | 10 | 8 | 19 | 58 | 36 | -7.6 | -39 | 0 | 10 | 2.4 |  |
| 2015 | 10 | 9 | 15 | 10 | 14 | -5.4 | -38.9 | 0 | 10 | 2.3 |  |
| 2015 | 10 | 16 | 19 | 45 | 44 | -3.8 | -40.5 | 0 | 15 | 1.3 |  |
| 2015 | 10 | 20 | 1 | 4 | 3 | -3.8 | -40.2 | 0 | 10 | 1.5 |  |
| 2015 | 10 | 25 | 5 | 15 | 51 | -3.3 | -40.6 | 0 | 5 | 2 |  |
| 2015 | 10 | 25 | 5 | 16 | 19 | -3.3 | -40.6 | 0 | 5 | 2.8 |  |
| 2015 | 10 | 25 | 5 | 19 | 50 | -3.3 | -40.6 | 0 | 5 | 2.3 |  |
| 2015 | 10 | 25 | 5 | 22 | 31 | -3.3 | -40.6 | 0 | 5 | 2.5 |  |
| 2015 | 11 | 6 | 4 | 20 | 37 | -15 | -40.1 | 0 | 5 | 3.2 |  |
| 2015 | 12 | 9 | 14 | 49 | 53 | -11 | -43.9 | 0 | 20 | 3.8 |  |
| 2015 | 12 | 13 | 18 | 37 | 34 | -3.7 | -38.8 | 0 | 20 | 2.4 |  |
| 2015 | 12 | 25 | 11 | 52 | 16 | -3.8 | -39.8 | 0 | 15 | 3.5 |  |
| 2016 | 1 | 1 | 15 | 25 | 25 | -14 | -43.2 | 0 | 25 | 2.8 |  |
| 2016 | 1 | 3 | 0 | 25 | 36 | -2.5 | -45.1 | 0 | 20 | 2.9 |  |
| 2016 | 1 | 6 | 20 | 38 | 42 | -3.8 | -39.9 | 0 | 15 | 2.6 |  |
| 2016 | 1 | 6 | 20 | 39 | 11 | -3.8 | -39.9 | 0 | 15 | 2.4 |  |
| 2016 | 1 | 9 | 1 | 58 | 22 | -13 | -44.7 | 0 | 50 | 2.5 |  |
| 2016 | 1 | 10 | 14 | 3 | 9 | -15 | -39.7 | 0 | 2 | 3 |  |
| 2016 | 1 | 11 | 3 | 31 | 6 | -15 | -39.8 | 0 | 2 | 2.1 |  |
| 2016 | 1 | 11 | 17 | 21 | 35 | -3.8 | -39.9 | 0 | 15 | 2.3 |  |
| 2016 | 1 | 12 | 18 | 46 | 28 | -15 | -39 | 0 | 10 | 1.8 |  |
| 2016 | 1 | 13 | 2 | 59 | 37 | -15 | -39.2 | 0 | 10 | 1.6 |  |
| 2016 | 1 | 18 | 3 | 19 | 2 | -3.8 | -39.9 | 0 | 15 | 2.1 |  |
| 2016 | 2 | 5 | 2 | 41 | 44 | -13 | -39.5 | 0 | 20 | 2 |  |
| 2016 | 2 | 14 | 6 | 5 | 33 | -13 | -39.5 | 0 | 20 | 2.1 |  |
| 2016 | 2 | 23 | 2 | 50 | 40 | -9.6 | -37.4 | 0 | 5 | 2.3 |  |
| 2016 | 2 | 23 | 14 | 38 | 0 | -8.3 | -36.1 | 0 | 3 | 2 |  |
| 2016 | 2 | 23 | 15 | 12 | 3 | -8.3 | -36.1 | 0 | 3 | 2.2 |  |
| 2016 | 2 | 23 | 15 | 13 | 8 | -8.3 | -36.1 | 0 | 3 | 1.8 |  |
| 2016 | 2 | 23 | 16 | 27 | 26 | -8.3 | -36.1 | 0 | 3 | 2.3 |  |
| 2016 | 2 | 23 | 18 | 35 | 26 | -8.3 | -36.2 | 0 | 3 | 3.8 |  |
| 2016 | 2 | 23 | 18 | 44 | 1 | -8.3 | -36.2 | 0 | 3 | 1.5 |  |
| 2016 | 2 | 23 | 18 | 44 | 34 | -8.3 | -36.1 | 0 | 3 | 2 |  |
| 2016 | 2 | 23 | 18 | 55 | 0 | -8.3 | -36.1 | 0 | 3 | 2.1 |  |
| 2016 | 2 | 23 | 19 | 21 | 51 | -8.3 | -36.2 | 0 | 3 | 2.4 |  |
| 2016 | 2 | 23 | 19 | 34 | 1 | -8.3 | -36.1 | 0 | 3 | 1.6 |  |
| 2016 | 2 | 23 | 19 | 56 | 53 | -8.3 | -36.2 | 0 | 3 | 1.8 |  |
| 2016 | 2 | 25 | 1 | 48 | 6 | -8.3 | -36.1 | 0 | 3 | 2.1 |  |
| 2016 | 2 | 25 | 4 | 14 | 17 | -8.3 | -36.1 | 0 | 3 | 1.9 |  |
| 2016 | 2 | 25 | 5 | 3 | 58 | -8.3 | -36.1 | 0 | 3 | 1.9 |  |
| 2016 | 2 | 25 | 16 | 51 | 33 | -8.3 | -36.1 | 0 | 3 | 2.3 |  |
| 2016 | 2 | 26 | 2 | 42 | 27 | -8.3 | -36.1 | 0 | 3 | 2 |  |
| 2016 | 3 | 1 | 12 | 25 | 6 | -8.3 | -36.1 | 0 | 3 | 2.2 |  |
| 2016 | 3 | 2 | 5 | 19 | 17 | -8.3 | -36.2 | 0 | 3 | 3.5 |  |
| 2016 | 3 | 4 | 0 | 10 | 56 | -9.6 | -36.8 | 0 | 30 | 2.2 |  |
| 2016 | 3 | 4 | 0 | 56 | 16 | -9.6 | -36.8 | 0 | 30 | 2 | 2 |
| 2016 | 3 | 5 | 20 | 41 | 54 | -8.3 | -36.1 | 0 | 3 | 2.2 |  |
| 2016 | 3 | 6 | 20 | 2 | 32 | -5.9 | -38.8 | 0 | 20 | 1.7 |  |
| 2016 | 3 | 6 | 20 | 8 | 45 | -5.9 | -38.9 | 0 | 20 | 2.6 |  |
| 2016 | 3 | 6 | 20 | 29 | 29 | -5.9 | -38.9 | 0 | 20 | 2.8 | 2 |
| 2016 | 3 | 9 | 19 | 0 | 3 | -9.4 | -35.8 | 0 | 5 | 2.6 |  |
| 2016 | 3 | 13 | 3 | 33 | 20 | -11 | -40.6 | 0 | 20 | 2.4 |  |
| 2016 | 3 | 14 | 9 | 48 | 16 | -5.9 | -38.8 | 0 | 20 | 3.4 |  |
| 2016 | 3 | 16 | 18 | 42 | 45 | -8.3 | -36.1 | 0 | 3 | 2.1 |  |
| 2016 | 3 | 18 | 4 | 59 | 59 | -8.4 | -36.1 | 0 | 20 | 2 |  |
| 2016 | 3 | 20 | 2 | 50 | 46 | -13 | -39.2 | 0 | 15 | 2.1 |  |
| 2016 | 3 | 23 | 8 | 26 | 19 | -8.3 | -36.2 | 0 | 15 | 2.3 |  |
| 2016 | 3 | 29 | 2 | 28 | 26 | -8.5 | -36 | 0 | 15 | 1.5 |  |
| 2016 | 3 | 29 | 2 | 30 | 14 | -8.3 | -36.1 | 0 | 15 | 1.5 |  |
| 2016 | 4 | 1 | 2 | 31 | 3 | -8.5 | -36 | 0 | 20 | 1.9 |  |
| 2016 | 4 | 1 | 2 | 44 | 46 | -8.3 | -36.1 | 0 | 20 | 1.8 |  |
| 2016 | 4 | 2 | 0 | 17 | 40 | -8.3 | -36.1 | 0 | 20 | 1.6 |  |
| 2016 | 4 | 8 | 6 | 59 | 7 | -4.9 | -38.1 | 0 | 20 | 2.1 |  |
| 2016 | 4 | 19 | 14 | 45 | 2 | -11 | -37.7 | 0 | 1 | 1.8 |  |
| 2016 | 4 | 23 | 7 | 59 | 41 | -9.6 | -36.5 | 0 | 15 | 2.8 |  |
| 2016 | 4 | 28 | 3 | 2 | 51 | -9.7 | -36 | 0 | 6 | 1.3 |  |
| 2016 | 4 | 28 | 7 | 26 | 9 | -15 | -37.5 | 0 | 35 | 2.8 |  |
| 2016 | 5 | 13 | 14 | 38 | 32 | -9.7 | -36.4 | 0 | 20 | 1.9 |  |
| 2016 | 6 | 24 | 3 | 57 | 21 | -8.3 | -36 | 0 | 5 | 1.8 |  |
| 2016 | 6 | 28 | 19 | 49 | 29 | -10 | -37 | 0 | 2 | 2.5 |  |
| 2016 | 7 | 8 | 4 | 31 | 5 | -12 | -44 | 0 | 20 | 2.3 |  |
| 2016 | 7 | 8 | 7 | 0 | 29 | -12 | -42.9 | 0 | 11 | 2.4 |  |
| 2016 | 7 | 9 | 4 | 15 | 30 | -12 | -42.9 | 0 | 11 | 2.6 |  |
| 2016 | 7 | 9 | 5 | 59 | 59 | -12 | -41.6 | 0 | 6 | 2.5 |  |
| 2016 | 7 | 9 | 6 | 31 | 2 | -12 | -46.2 | 0 | 20 | 1.9 |  |
| 2016 | 7 | 9 | 8 | 15 | 31 | -12 | -43.6 | 0 | 30 | 2.2 |  |
| 2016 | 8 | 7 | 14 | 0 | 42 | -15 | -39.5 | 0 | 20 | 2.2 |  |
| 2016 | 8 | 11 | 0 | 44 | 56 | -17 | -40.1 | 0 | 10 | 2.8 |  |
| 2016 | 8 | 12 | 13 | 11 | 24 | -9.6 | -37 | 0 | 12 | 2.4 |  |
| 2016 | 8 | 26 | 20 | 15 | 22 | -11 | -37 | 0 | 15 | 2.4 |  |
| 2016 | 9 | 12 | 15 | 17 | 39 | -9.7 | -36.3 | 0 | 2 | 1.3 |  |
| 2016 | 9 | 12 | 20 | 22 | 34 | -9.7 | -36.4 | 0 | 2 | 1.4 |  |
| 2016 | 9 | 12 | 21 | 46 | 32 | -9.6 | -36.4 | 0 | 2 | 1.3 |  |
| 2016 | 9 | 12 | 23 | 12 | 13 | -9.7 | -36.4 | 0 | 2 | 1.5 |  |
| 2016 | 9 | 14 | 20 | 48 | 37 | -9.3 | -36.2 | 0 | 2 | 1.6 |  |
| 2016 | 9 | 15 | 2 | 21 | 46 | -9.4 | -36.2 | 0 | 2 | 1.6 |  |
| 2016 | 9 | 15 | 2 | 22 | 40 | -9.5 | -36.2 | 0 | 2 | 1.4 |  |
| 2016 | 9 | 18 | 14 | 26 | 9 | -17 | -38 | 0 | 20 | 2.9 |  |
| 2016 | 9 | 20 | 5 | 18 | 49 | -10 | -43.9 | 0 | 40 | 2.2 |  |
| 2016 | 9 | 20 | 20 | 16 | 57 | -8.7 | -35.3 | 0 | 10 | 1.2 |  |
| 2016 | 10 | 9 | 12 | 3 | 48 | -5.5 | -36.2 | 0 | 10 | 2 |  |
| 2016 | 10 | 9 | 18 | 46 | 9 | -5.5 | -36.2 | 0 | 10 | 1.7 |  |
| 2016 | 10 | 24 | 20 | 51 | 54 | -6.4 | -39.2 | 0 | 20 | 3.2 |  |
| 2016 | 11 | 16 | 4 | 15 | 39 | -10 | -37.2 | 0 | 20 | 3.1 |  |
| 2017 | 1 | 3 | 12 | 43 | 47 | -3.1 | -43.9 | 12 | 2 | 4.3 | 5 |
| 2017 | 1 | 3 | 12 | 50 | 49 | -3.1 | -43.9 | 12 | 2 | 3.1 |  |
| 2017 | 1 | 3 | 13 | 2 | 10 | -3.1 | -43.9 | 12 | 2 | 1.3 |  |
| 2017 | 1 | 3 | 13 | 4 | 17 | -3.1 | -43.9 | 12 | 2 | 2.2 |  |
| 2017 | 1 | 3 | 13 | 15 | 15 | -3.1 | -43.9 | 12 | 2 | 2.2 |  |
| 2017 | 1 | 3 | 13 | 23 | 8 | -3.1 | -43.9 | 12 | 2 | 1.7 |  |
| 2017 | 1 | 3 | 14 | 8 | 22 | -3.1 | -43.9 | 12 | 2 | 2.2 |  |
| 2017 | 1 | 3 | 14 | 19 | 12 | -3.1 | -43.9 | 12 | 2 | 1.5 |  |
| 2017 | 1 | 3 | 16 | 21 | 24 | -3.1 | -43.9 | 12 | 2 | 2 |  |
| 2017 | 1 | 3 | 18 | 35 | 58 | -3.1 | -43.9 | 12 | 2 | 0.9 |  |
| 2017 | 1 | 3 | 21 | 5 | 2 | -3.1 | -43.9 | 12 | 2 | 2.2 |  |
| 2017 | 1 | 4 | 0 | 30 | 31 | -3.1 | -43.9 | 12 | 2 | 2.5 |  |
| 2017 | 1 | 4 | 9 | 21 | 5 | -3.1 | -43.9 | 12 | 2 | 2.7 |  |
| 2017 | 1 | 4 | 10 | 8 | 43 | -3.1 | -43.9 | 12 | 2 | 2.5 |  |
| 2017 | 1 | 7 | 2 | 27 | 41 | -3.1 | -43.9 | 12 | 2 | 2.1 |  |
| 2017 | 1 | 10 | 4 | 30 | 44 | -3.1 | -43.9 | 12 | 2 | 0.2 |  |
| 2017 | 1 | 11 | 22 | 54 | 4 | -3.1 | -43.9 | 12 | 2 | 1.2 |  |
| 2017 | 1 | 11 | 23 | 11 | 56 | -3.1 | -43.9 | 12 | 2 | 0.8 |  |
| 2017 | 1 | 12 | 8 | 55 | 2 | -3.1 | -43.9 | 12 | 2 | 1 |  |
| 2017 | 1 | 17 | 0 | 16 | 57 | -3.1 | -43.9 | 12 | 2 | 0.2 |  |
| 2017 | 1 | 19 | 7 | 14 | 35 | -3.1 | -43.9 | 12 | 2 | 0.9 |  |
| 2017 | 1 | 19 | 23 | 35 | 57 | -3.1 | -43.9 | 12 | 2 | 0.1 |  |
| 2017 | 1 | 22 | 14 | 40 | 52 | -3.1 | -43.9 | 12 | 2 | 1.3 |  |
| 2017 | 2 | 2 | 7 | 15 | 11 | -3.1 | -43.9 | 12 | 0 | 0.6 |  |
| 2017 | 3 | 1 | 8 | 28 | 11 | -13 | -39.7 | 0 | 10 | 2.2 |  |
| 2017 | 3 | 5 | 8 | 46 | 11 | -3.5 | -40.2 | 0 | 34 | 2.6 |  |
| 2017 | 4 | 5 | 17 | 20 | 59 | -11 | -45.1 | 0 | 30 | 3 |  |
| 2017 | 4 | 5 | 23 | 37 | 28 | -10 | -44.5 | 0 | 30 | 2.4 |  |
| 2017 | 4 | 10 | 1 | 59 | 51 | -3.2 | -40.1 | 0 | 6 | 3.1 |  |
| 2017 | 6 | 17 | 17 | 42 | 49 | -13 | -44.5 | 0 | 20 | 2.6 |  |
| 2017 | 6 | 19 | 18 | 10 | 57 | -13 | -44.5 | 0 | 20 | 2.6 |  |
| 2017 | 7 | 25 | 20 | 49 | 51 | -4.1 | -38.2 | 0 | 48 | 3.3 |  |
| 2017 | 7 | 26 | 1 | 12 | 37 | -11 | -43.9 | 0 | 30 | 2 |  |
| 2017 | 9 | 13 | 21 | 4 | 45 | -3.1 | -43.9 | 0 | 5 | 2.7 |  |
| 2017 | 9 | 20 | 7 | 9 | 20 | -8.3 | -36.8 | 0 | 45 | 3 |  |
| 2017 | 10 | 10 | 1 | 58 | 39 | -8.3 | -36.1 | 0 | 10 | 1.7 |  |
| 2017 | 10 | 16 | 11 | 0 | 10 | -4.3 | -38.4 | 0 | 5 | 3 |  |
| 2017 | 11 | 4 | 11 | 30 | 52 | -18 | -39.1 | 0 | 40 | 1.9 |  |
| 2017 | 12 | 2 | 18 | 2 | 34 | -3 | -44.2 | 0 | 2 | 2.2 |  |
| 2018 | 1 | 25 | 1 | 27 | 20 | -17 | -38.9 | 0 | 15 | 3.1 |  |
| 2018 | 2 | 4 | 13 | 20 | 21 | -15 | -40 | 0 | 10 | 1.4 | 4 |
| 2018 | 2 | 23 | 16 | 23 | 0 | -12 | -40.2 | 1.5 | 1 | 2 | 3 |
| 2018 | 2 | 27 | 3 | 31 | 46 | -15 | -40.4 | 0 | 16 | 1.3 |  |
| 2018 | 3 | 3 | 17 | 30 | 30 | -9.6 | -35.7 | 0 | 5 | 2.2 | 4-5 |
| 2018 | 3 | 10 | 15 | 51 | 57 | -15 | -39.5 | 0 | 10 | 1.8 |  |
| 2018 | 3 | 10 | 19 | 32 | 48 | -5.6 | -35.6 | 0 | 5 | 1.8 |  |
| 2018 | 3 | 10 | 21 | 5 | 30 | -5.5 | -35.5 | 0 | 5 | 1.7 |  |
| 2018 | 3 | 11 | 8 | 16 | 23 | -5.5 | -35.7 | 5 | 5 | 2 |  |
| 2018 | 3 | 13 | 6 | 35 | 6 | -5.5 | -35.6 | 0 | 5 | 2.4 |  |
| 2018 | 3 | 14 | 21 | 25 | 18 | -8.2 | -35.7 | 0 | 10 | 1.5 |  |
| 2018 | 3 | 18 | 9 | 38 | 41 | -5.6 | -35.5 | 0 | 5 | 1.9 |  |
| 2018 | 3 | 21 | 18 | 46 | 14 | -2.4 | -44.4 | 0 | 30 | 3.2 |  |
| 2018 | 4 | 14 | 20 | 50 | 18 | -3.6 | -40.4 | 0 | 12 | 1.3 |  |
| 2018 | 4 | 24 | 7 | 47 | 48 | -5.6 | -36.1 | 0 | 10 | 1.5 |  |
| 2018 | 5 | 11 | 12 | 29 | 7 | -5 | -38.1 | 0 | 25 | 2.3 | 2 |
| 2018 | 5 | 16 | 12 | 24 | 52 | -9.8 | -37.2 | 0 | 10 | 1.7 |  |
| 2018 | 5 | 20 | 4 | 47 | 21 | -5.5 | -35.4 | 0 | 10 | 1.9 |  |
| 2018 | 5 | 24 | 0 | 8 | 42 | -3.8 | -39.9 | 0 | 30 | 2.5 | 2 |
| 2018 | 6 | 13 | 11 | 9 | 50 | -4.8 | -38.1 | 0 | 10 | 1.8 |  |
| 2018 | 7 | 10 | 11 | 55 | 52 | -5.7 | -39.6 | 0 | 5 | 1.5 |  |
| 2018 | 7 | 10 | 20 | 46 | 8 | -11 | -37.1 | 0 | 10 | 2.4 |  |
| 2018 | 7 | 25 | 11 | 24 | 29 | -4.2 | -38.9 | 0 | 10 | 1.9 |  |
| 2018 | 8 | 13 | 18 | 19 | 54 | -8.2 | -37.2 | 0 | 20 | 1.4 |  |
| 2018 | 9 | 18 | 19 | 20 | 26 | -3.4 | -40.8 | 0 | 3 | 3.2 |  |
| 2018 | 9 | 21 | 20 | 12 | 44 | -11 | -37.4 | 0 | 10 | 2.5 |  |
| 2018 | 9 | 27 | 9 | 59 | 9 | -12 | -38.9 | 0 | 27 | 1.4 |  |
| 2018 | 10 | 8 | 23 | 51 | 27 | -3.6 | -40.5 | 0 | 35 | 1.5 |  |
| 2018 | 10 | 21 | 15 | 46 | 18 | -9.4 | -35.7 | 0 | 10 | 1.8 |  |
| 2018 | 10 | 24 | 21 | 34 | 22 | -13 | -39.5 | 0 | 10 | 2.2 | 2 |
| 2018 | 10 | 27 | 6 | 36 | 57 | -13 | -39.6 | 0 | 10 | 3.2 | 4 |
| 2018 | 12 | 10 | 16 | 36 | 50 | -14 | -40.6 | 0 | 15 | 1.7 |  |
| 2018 | 12 | 14 | 16 | 53 | 43 | -10 | -37.1 | 0 | 10 | 2.7 |  |
| 2018 | 12 | 14 | 19 | 44 | 2 | -8.2 | -37.2 | 0 | 20 | 1.8 |  |
| 2018 | 12 | 18 | 16 | 19 | 18 | -14 | -40.7 | 0 | 15 | 1.7 |  |
| 2019 | 1 | 9 | 8 | 55 | 52 | -11 | -37.5 | 0 | 15 | 2.7 | 3 |
| 2019 | 1 | 12 | 9 | 54 | 7 | -11 | -37.4 | 0 | 20 | 2.2 | 2 |
| 2019 | 1 | 19 | 7 | 35 | 40 | -11 | -37.3 | 0 | 20 | 2 | 2 |
| 2019 | 1 | 19 | 11 | 0 | 0 | -11 | -37.3 | 0 | 20 | 1 | 2 |
| 2019 | 1 | 28 | 14 | 5 | 23 | -17 | -39.8 | 0 | 1 | 2.5 | 5 |
| 2019 | 1 | 30 | 5 | 42 | 47 | -9.6 | -35.9 | 0 | 6 | 1.4 |  |
| 2019 | 1 | 30 | 5 | 47 | 16 | -9.6 | -35.9 | 0 | 6 | 1.5 |  |
| 2019 | 1 | 30 | 5 | 53 | 22 | -9.6 | -35.8 | 0 | 6 | 1.4 |  |
| 2019 | 1 | 30 | 6 | 2 | 18 | -9.6 | -35.9 | 0 | 6 | 1.5 |  |
| 2019 | 2 | 11 | 15 | 26 | 21 | -17 | -39.8 | 0 | 6 | 2.1 | 4 |
| 2019 | 3 | 14 | 8 | 52 | 56 | -5.8 | -37.6 | 0 | 28 | 2.2 |  |
| 2019 | 3 | 18 | 2 | 17 | 49 | -10 | -36.8 | 0 | 20 | 2.5 | 4 |
| 2019 | 3 | 18 | 2 | 20 | 49 | -5.1 | -39.4 | 0 | 20 | 1.5 |  |
| 2019 | 3 | 18 | 2 | 41 | 55 | -5.2 | -39.4 | 0 | 20 | 2.3 | 2 |
| 2019 | 3 | 18 | 11 | 24 | 2 | -10 | -36.8 | 0 | 20 | 1.6 | 4 |
| 2019 | 3 | 18 | 18 | 2 | 17 | -10 | -36.8 | 0 | 20 | 2.5 |  |
| 2019 | 3 | 18 | 18 | 11 | 24 | -10 | -36.6 | 0 | 10 | 1.6 |  |
| 2019 | 3 | 18 | 19 | 28 | 2 | -5.2 | -39.4 | 0 | 20 | 2.4 | 2 |
| 2019 | 3 | 19 | 11 | 19 | 4 | -5.2 | -39.4 | 0 | 20 | 2.3 |  |
| 2019 | 3 | 20 | 10 | 32 | 37 | -5.2 | -39.4 | 0 | 20 | 2.9 |  |
| 2019 | 3 | 23 | 6 | 36 | 9 | -5.1 | -39.4 | 0 | 20 | 2 |  |
| 2019 | 3 | 23 | 6 | 57 | 26 | -5.1 | -39.5 | 0 | 20 | 2.1 |  |
| 2019 | 3 | 23 | 19 | 18 | 38 | -5.2 | -39.4 | 0 | 20 | 2.5 |  |
| 2019 | 3 | 30 | 11 | 8 | 6 | -5.1 | -39.5 | 0 | 20 | 3.2 |  |
| 2019 | 4 | 19 | 2 | 30 | 31 | -5.1 | -39.5 | 0 | 20 | 3.4 | 5 |
| 2019 | 5 | 16 | 14 | 45 | 58 | -6.1 | -36 | 0 | 6 | 2.1 |  |
| 2019 | 6 | 2 | 17 | 52 | 3 | -11 | -37.3 | 0 | 10 | 2.4 | 3 |
| 2019 | 6 | 6 | 22 | 55 | 31 | -14 | -44.8 | 0 | 2 | 2.9 |  |
| 2019 | 7 | 13 | 12 | 56 | 20 | -5.5 | -35.8 | 0 | 6 | 2.6 |  |
| 2019 | 7 | 15 | 5 | 49 | 57 | -5.1 | -39.6 | 0 | 20 | 1.8 |  |
| 2019 | 7 | 20 | 11 | 10 | 18 | -5.7 | -35.8 | 0 | 2 | 2 |  |
| 2019 | 7 | 22 | 10 | 7 | 4 | -5.6 | -36.5 | 0 | 10 | 1.8 |  |
| 2019 | 7 | 24 | 5 | 10 | 19 | -5 | -39.6 | 0 | 20 | 2 | 2 |
| 2019 | 7 | 24 | 5 | 46 | 3 | -5.1 | -39.5 | 0 | 20 | 2.7 | 2 |
| 2019 | 7 | 26 | 0 | 44 | 35 | -5 | -39.6 | 0 | 20 | 3.1 | 2-3 |
| 2019 | 8 | 3 | 23 | 41 | 19 | -5.6 | -35.5 | 0 | 3 | 2 |  |
| 2019 | 8 | 7 | 19 | 47 | 53 | -9.7 | -35.9 | 0 | 20 | 1.9 |  |
| 2019 | 8 | 9 | 11 | 9 | 43 | -4.2 | -38.1 | 0 | 3 | 1.5 |  |
| 2019 | 9 | 2 | 6 | 7 | 18 | -17 | -40.5 | 0 | 17 | 1.7 |  |
| 2019 | 9 | 5 | 3 | 37 | 4 | -5.7 | -37.5 | 0 | 27 | 2.8 |  |
| 2019 | 9 | 13 | 22 | 46 | 41 | -3 | -44.7 | 0 | 50 | 3.2 | 3 |
| 2019 | 9 | 15 | 14 | 14 | 55 | -5.5 | -35.6 | 0 | 8 | 1.3 |  |
| 2019 | 9 | 27 | 23 | 46 | 49 | -9.9 | -36.5 | 0 | 8 | 2.3 |  |
| 2019 | 10 | 14 | 1 | 47 | 35 | -4.8 | -41.2 | 0 | 2 | 2.7 |  |
| 2019 | 10 | 18 | 23 | 0 | 47 | -5.6 | -35.6 | 0 | 6 | 0.9 |  |
| 2019 | 10 | 19 | 23 | 33 | 52 | -5.6 | -35.6 | 0 | 6 | 0.9 |  |
| 2019 | 11 | 1 | 11 | 38 | 49 | -5.7 | -37.5 | 0 | 26 | 2.1 |  |
| 2019 | 11 | 3 | 15 | 33 | 36 | -4.7 | -38 | 0 | 30 | 2.4 | 2 |
| 2019 | 11 | 3 | 15 | 42 | 59 | -4.8 | -38 | 0 | 30 | 2.4 | 2 |
| 2019 | 11 | 9 | 7 | 30 | 24 | -13 | -39.6 | 0 | 30 | 3.3 | 4 |
| 2019 | 11 | 16 | 8 | 31 | 25 | -4.2 | -38.3 | 0 | 46 | 1 |  |
| 2019 | 11 | 21 | 21 | 38 | 55 | -14 | -44.8 | 0 | 25 | 3 |  |
| 2019 | 11 | 26 | 20 | 51 | 45 | -13 | -39.5 | 0 | 32 | 2 | 2 |
| 2019 | 11 | 30 | 21 | 58 | 4 | -13 | -39.5 | 0 | 32 | 2 | 2 |
| 2019 | 12 | 3 | 21 | 4 | 39 | -3.3 | -44.1 | 0 | 2 | 3.2 |  |
| 2019 | 12 | 26 | 21 | 20 | 26 | -8 | -36.2 | 0 | 31 | 1.9 | 4 |
| 2019 | 12 | 30 | 1 | 31 | 2 | -8.3 | -36 | 0 | 33 | 1.8 | 3-4 |
| 2019 | 12 | 31 | 3 | 24 | 30 | -8.1 | -36.1 | 0 | 33 | 1.9 |  |
| 2020 | 1 | 10 | 19 | 50 | 57 | -4.8 | -38 | 0 | 21 | 2 |  |
| 2020 | 1 | 13 | 12 | 46 | 43 | -5.6 | -35.5 | 0 | 6 | 1.6 |  |
| 2020 | 1 | 19 | 7 | 58 | 36 | -5.1 | -35.4 | 0 | 11 | 1.3 |  |
| 2020 | 1 | 31 | 19 | 59 | 35 | -16 | -40.1 | 0 | 5 | 1.8 |  |
| 2020 | 2 | 16 | 21 | 23 | 38 | -3.1 | -40.7 | 0 | 12 | 2 |  |
| 2020 | 2 | 28 | 8 | 23 | 31 | -8.3 | -36 | 0 | 33 | 1.7 |  |
| 2020 | 3 | 11 | 1 | 11 | 26 | -5.4 | -35.6 | 0 | 10 | 1.9 |  |
| 2020 | 3 | 17 | 2 | 29 | 22 | -5.4 | -35.6 | 0 | 10 | 1.7 |  |
| 2020 | 3 | 29 | 1 | 54 | 49 | -9.9 | -39.9 | 0 | 46 | 3 |  |
| 2020 | 3 | 30 | 3 | 30 | 11 | -5.5 | -35.6 | 0 | 6 | 1.7 |  |
| 2020 | 4 | 6 | 3 | 21 | 20 | -5.5 | -35.7 | 0 | 6 | 1.8 |  |
| 2020 | 4 | 7 | 12 | 12 | 15 | -4.1 | -39.2 | 0 | 19 | 1.6 | 1 |
| 2020 | 4 | 14 | 23 | 13 | 56 | -3.2 | -40.7 | 0 | 14 | 2 | 1 |
| 2020 | 4 | 26 | 4 | 45 | 5 | -4.7 | -38.1 | 0 | 24 | 1.6 |  |
| 2020 | 4 | 26 | 5 | 10 | 54 | -4.8 | -38.1 | 0 | 24 | 1.3 |  |
| 2020 | 4 | 26 | 11 | 25 | 20 | -4.3 | -38.2 | 0 | 30 | 1.8 |  |
| 2020 | 4 | 27 | 6 | 20 | 57 | -4.7 | -38.1 | 0 | 24 | 2 |  |
| 2020 | 4 | 29 | 2 | 45 | 24 | -8.3 | -36 | 0 | 33 | 1.7 |  |
| 2020 | 4 | 30 | 6 | 14 | 2 | -4.7 | -35.1 | 0 | 23 | 1.4 |  |
| 2020 | 5 | 3 | 13 | 16 | 7 | -5 | -39.5 | 0 | 20 | 1.8 |  |
| 2020 | 5 | 6 | 1 | 26 | 45 | -3.6 | -40.5 | 0 | 9 | 1.8 |  |
| 2020 | 5 | 6 | 1 | 31 | 39 | -3.6 | -40.5 | 0 | 9 | 1.6 |  |
| 2020 | 5 | 7 | 14 | 18 | 6 | -3.6 | -40.5 | 0 | 9 | 1.8 |  |
| 2020 | 5 | 8 | 10 | 14 | 12 | -4.7 | -38.1 | 0 | 24 | 1.7 |  |
| 2020 | 5 | 10 | 1 | 34 | 36 | -4.3 | -38.2 | 0 | 4 | 1.6 |  |
| 2020 | 5 | 11 | 4 | 20 | 4 | -5.3 | -40.7 | 0 | 20 | 1.6 |  |
| 2020 | 5 | 14 | 21 | 24 | 46 | -5.6 | -37.5 | 0 | 6 | 1.8 |  |
| 2020 | 5 | 15 | 20 | 42 | 26 | -8.3 | -36.5 | 0 | 11 | 1.6 |  |
| 2020 | 5 | 17 | 19 | 23 | 23 | -5.8 | -36.2 | 0 | 5 | 1.9 |  |
| 2020 | 5 | 18 | 4 | 45 | 4 | -5.7 | -37.5 | 0 | 6 | 2 |  |
| 2020 | 5 | 24 | 7 | 34 | 37 | -5.7 | -36.9 | 0 | 3 | 1.7 |  |
| 2020 | 5 | 26 | 5 | 54 | 23 | -3.9 | -40 | 0 | 10 | 1.2 |  |
| 2020 | 5 | 27 | 2 | 20 | 24 | -5.9 | -37.4 | 0 | 2 | 1.6 |  |
| 2020 | 5 | 29 | 19 | 37 | 57 | -5.7 | -37.5 | 0 | 6 | 1.5 |  |
| 2020 | 5 | 31 | 13 | 2 | 55 | -3.3 | -40.5 | 0 | 8 | 1.6 |  |
| 2020 | 6 | 28 | 2 | 23 | 5 | -3.3 | -40.5 | 0 | 8 | 2.2 |  |
| 2020 | 6 | 29 | 6 | 59 | 14 | -3.4 | -40.3 | 0 | 4 | 1.3 |  |
| 2020 | 6 | 29 | 7 | 36 | 22 | -3.4 | -40.2 | 0 | 4 | 1.4 |  |
| 2020 | 6 | 29 | 20 | 7 | 40 | -3.9 | -40.4 | 0 | 12 | 1.9 |  |
| 2020 | 7 | 3 | 5 | 32 | 27 | -5.6 | -37.5 | 0 | 27 | 1.9 |  |
| 2020 | 7 | 4 | 14 | 35 | 36 | -3.1 | -40.8 | 0 | 13 | 1.7 |  |
| 2020 | 7 | 7 | 23 | 22 | 3 | -4.3 | -38.3 | 0 | 33 | 2.5 |  |
| 2020 | 7 | 8 | 13 | 38 | 18 | -5.5 | -36.1 | 0 | 6 | 1.6 |  |
| 2020 | 7 | 9 | 9 | 53 | 10 | -8.3 | -36 | 0 | 33 | 1.6 |  |
| 2020 | 7 | 12 | 3 | 58 | 8 | -9.5 | -40.6 | 0 | 23 | 2.6 |  |
| 2020 | 7 | 17 | 14 | 48 | 15 | -3.5 | -40.7 | 0 | 12 | 1.9 |  |
| 2020 | 7 | 20 | 2 | 41 | 51 | -14 | -44.1 | 0 | 23 | 2.5 |  |
| 2020 | 7 | 20 | 13 | 18 | 45 | -3.9 | -40.3 | 0 | 10 | 2.5 |  |
| 2020 | 7 | 25 | 20 | 24 | 1 | -8.4 | -36.6 | 0 | 39 | 2.4 |  |
| 2020 | 7 | 28 | 14 | 33 | 0 | -6 | -38.2 | 0 | 18 | 2.1 |  |
| 2020 | 7 | 28 | 14 | 49 | 30 | -5.7 | -35.9 | 0 | 1 | 1.7 |  |
| 2020 | 7 | 28 | 19 | 33 | 50 | -5.8 | -35.9 | 0 | 1 | 1.8 |  |
| 2020 | 7 | 29 | 8 | 19 | 37 | -15 | -38.1 | 0 | 24 | 3.9 |  |
| 2020 | 8 | 1 | 23 | 55 | 24 | -15 | -40.1 | 0 | 10 | 1.9 |  |
| 2020 | 8 | 4 | 17 | 3 | 41 | -8.7 | -36.3 | 0 | 30 | 2 |  |
| 2020 | 8 | 5 | 20 | 17 | 28 | -8.6 | -35.8 | 0 | 37 | 1.8 |  |
| 2020 | 8 | 12 | 12 | 59 | 26 | -5.9 | -36 | 0 | 2 | 1.6 |  |
| 2020 | 8 | 12 | 19 | 32 | 57 | -5.9 | -36 | 0 | 2 | 1.7 |  |
| 2020 | 8 | 17 | 2 | 24 | 48 | -5.5 | -36.1 | 0 | 6 | 1.8 |  |
| 2020 | 8 | 17 | 9 | 6 | 11 | -3.8 | -39.8 | 0 | 9 | 1.8 |  |
| 2020 | 8 | 19 | 21 | 51 | 2 | -13 | -39 | 0 | 35 | 1.6 |  |
| 2020 | 8 | 23 | 9 | 26 | 57 | -8.3 | -36 | 0 | 33 | 1.6 |  |
| 2020 | 8 | 24 | 0 | 57 | 40 | -3.9 | -40.4 | 0 | 12 | 2 |  |
| 2020 | 8 | 26 | 7 | 6 | 44 | -3.7 | -40.5 | 0 | 9 | 2 |  |
| 2020 | 8 | 26 | 14 | 47 | 43 | -3.9 | -39.5 | 0 | 14 | 1.9 |  |
| 2020 | 8 | 27 | 6 | 1 | 52 | -8.3 | -36 | 0 | 33 | 1.9 | 3-4 |
| 2020 | 8 | 27 | 6 | 18 | 51 | -8.3 | -36 | 0 | 33 | 1.6 | 3-4 |
| 2020 | 8 | 27 | 11 | 1 | 22 | -8.3 | -36 | 0 | 33 | 1.6 |  |
| 2020 | 8 | 29 | 5 | 23 | 29 | -5.5 | -36.1 | 0 | 6 | 2.2 |  |
| 2020 | 8 | 30 | 10 | 43 | 44 | -13 | -39.6 | 0 | 32 | 2.5 |  |
| 2020 | 8 | 30 | 10 | 44 | 28 | -13 | -39.5 | 1.2 | 3 | 4.4 | 6 |
| 2020 | 8 | 30 | 11 | 18 | 7 | -13 | -39.6 | 0 | 32 | 3.7 |  |
| 2020 | 8 | 30 | 11 | 25 | 15 | -13 | -39.6 | 0 | 32 | 2.7 |  |
| 2020 | 8 | 30 | 11 | 57 | 9 | -13 | -39.6 | 0 | 32 | 2.8 |  |
| 2020 | 8 | 30 | 12 | 8 | 14 | -13 | -39.6 | 0 | 32 | 2.3 |  |
| 2020 | 8 | 30 | 12 | 11 | 42 | -13 | -39.6 | 0 | 32 | 2.2 |  |
| 2020 | 8 | 30 | 12 | 21 | 48 | -13 | -39.6 | 0 | 32 | 1.8 |  |
| 2020 | 8 | 30 | 13 | 41 | 43 | -13 | -39.6 | 0 | 32 | 1.6 |  |
| 2020 | 8 | 30 | 21 | 9 | 18 | -13 | -39.6 | 0 | 32 | 1.6 |  |
| 2020 | 8 | 30 | 21 | 11 | 44 | -13 | -39.6 | 0 | 32 | 2.3 |  |
| 2020 | 8 | 31 | 6 | 41 | 48 | -13 | -39.6 | 0 | 32 | 3.5 |  |
| 2020 | 8 | 31 | 7 | 27 | 28 | -5.5 | -36.1 | 0 | 6 | 1.8 |  |
| 2020 | 8 | 31 | 15 | 36 | 52 | -3.6 | -40.7 | 0 | 12 | 2.7 |  |
| 2020 | 8 | 31 | 17 | 26 | 20 | -3.5 | -40 | 0 | 4 | 2 |  |
| 2020 | 9 | 1 | 6 | 31 | 36 | -13 | -39.6 | 0 | 32 | 1.8 |  |
| 2020 | 9 | 1 | 9 | 36 | 28 | -13 | -39.6 | 0 | 32 | 2.5 |  |
| 2020 | 9 | 1 | 11 | 32 | 45 | -10 | -36.9 | 0 | 25 | 2.4 |  |
| 2020 | 9 | 2 | 1 | 1 | 19 | -5.5 | -36.1 | 0 | 6 | 2 |  |
| 2020 | 9 | 2 | 16 | 36 | 33 | -13 | -39.6 | 0 | 32 | 1.7 |  |
| 2020 | 9 | 3 | 2 | 21 | 57 | -10 | -36.9 | 0 | 25 | 2.1 |  |
| 2020 | 9 | 3 | 3 | 12 | 50 | -5.5 | -36 | 0 | 6 | 1.9 |  |
| 2020 | 9 | 4 | 2 | 30 | 48 | -9.8 | -37 | 0 | 21 | 2 |  |
| 2020 | 9 | 5 | 20 | 18 | 21 | -9.8 | -37 | 0 | 21 | 2.2 |  |
| 2020 | 9 | 6 | 0 | 33 | 24 | -9.8 | -37 | 0 | 21 | 2.1 |  |
| 2020 | 9 | 6 | 3 | 58 | 35 | -9.8 | -37 | 0 | 21 | 1.8 |  |
| 2020 | 9 | 9 | 2 | 18 | 11 | -8.3 | -36 | 0 | 33 | 1.9 |  |
| 2020 | 9 | 9 | 3 | 9 | 13 | -8.3 | -36 | 0 | 33 | 1.7 |  |
| 2020 | 9 | 9 | 5 | 37 | 29 | -8.3 | -36 | 0 | 33 | 1.5 |  |
| 2020 | 9 | 9 | 5 | 40 | 12 | -8.3 | -36 | 0 | 33 | 1.6 |  |
| 2020 | 9 | 9 | 13 | 35 | 13 | -8.3 | -36.1 | 0 | 33 | 2.5 |  |
| 2020 | 9 | 10 | 6 | 29 | 43 | -8.3 | -36.1 | 0 | 33 | 1.6 |  |
| 2020 | 9 | 10 | 19 | 44 | 43 | -7.9 | -38.8 | 0 | 24 | 2.3 |  |
| 2020 | 9 | 11 | 5 | 25 | 8 | -8.3 | -36.1 | 0 | 33 | 1.6 |  |
| 2020 | 9 | 11 | 5 | 29 | 10 | -8.3 | -36.1 | 0 | 33 | 2.3 |  |
| 2020 | 9 | 11 | 9 | 57 | 6 | -8.3 | -36.1 | 0 | 33 | 2.4 |  |
| 2020 | 9 | 12 | 2 | 29 | 7 | -5.5 | -36.1 | 0 | 6 | 2.2 |  |
| 2020 | 9 | 14 | 7 | 41 | 27 | -8.3 | -36.1 | 0 | 33 | 1.6 |  |
| 2020 | 9 | 14 | 10 | 51 | 52 | -8.3 | -36.1 | 0 | 33 | 1.8 |  |
| 2020 | 9 | 15 | 13 | 59 | 48 | -8.4 | -38.8 | 0 | 16 | 2 |  |
| 2020 | 9 | 15 | 20 | 10 | 17 | -5.5 | -35.8 | 0 | 6 | 2 |  |
| 2020 | 9 | 17 | 14 | 43 | 33 | -10 | -37 | 0 | 24 | 1.9 |  |
| 2020 | 9 | 19 | 9 | 6 | 10 | -3.1 | -41.2 | 0 | 19 | 2.7 |  |
| 2020 | 9 | 21 | 5 | 0 | 37 | -5.6 | -36.3 | 0 | 8 | 1.7 |  |
| 2020 | 9 | 21 | 14 | 53 | 49 | -4 | -40.4 | 0 | 12 | 2.4 |  |
| 2020 | 9 | 23 | 5 | 31 | 13 | -8.3 | -36 | 0 | 33 | 1.6 |  |
| 2020 | 9 | 26 | 4 | 59 | 48 | -5.5 | -36 | 0 | 6 | 1.6 |  |
| 2020 | 9 | 26 | 6 | 3 | 19 | -5.5 | -36.1 | 0 | 6 | 2 |  |
| 2020 | 9 | 29 | 2 | 28 | 0 | -3 | -40.7 | 0 | 12 | 2 |  |
| 2020 | 9 | 29 | 2 | 30 | 53 | -3 | -40.7 | 0 | 12 | 1.5 |  |
| 2020 | 9 | 29 | 19 | 52 | 57 | -5.5 | -36 | 0 | 1 | 1.6 |  |
| 2020 | 9 | 29 | 20 | 2 | 51 | -5.9 | -35.9 | 0 | 6 | 2.3 |  |
| 2020 | 9 | 29 | 20 | 15 | 12 | -3.6 | -40.7 | 0 | 12 | 1.9 |  |
| 2020 | 10 | 12 | 18 | 54 | 18 | -8.3 | -36 | 0 | 33 | 1.6 |  |
| 2020 | 10 | 13 | 7 | 1 | 4 | -5.5 | -36.1 | 0 | 6 | 2.1 |  |
| 2020 | 10 | 15 | 14 | 56 | 15 | -3.6 | -40.7 | 0 | 12 | 2.1 |  |
| 2020 | 10 | 15 | 15 | 51 | 24 | -8.8 | -35.2 | 0 | 23 | 2.1 |  |
| 2020 | 10 | 29 | 18 | 27 | 32 | -3.9 | -40.5 | 0 | 17 | 2 |  |
| 2020 | 10 | 29 | 19 | 25 | 34 | -5.7 | -35.9 | 0 | 1 | 2.2 |  |
| 2020 | 11 | 1 | 1 | 3 | 13 | -8.3 | -36 | 0 | 33 | 1.5 |  |
| 2020 | 11 | 1 | 1 | 4 | 18 | -8.3 | -36 | 0 | 33 | 1.5 |  |
| 2020 | 11 | 1 | 2 | 25 | 9 | -8.3 | -35.9 | 0 | 33 | 1.3 |  |
| 2020 | 11 | 12 | 9 | 35 | 30 | -8.3 | -36 | 0 | 33 | 1.9 |  |
| 2020 | 11 | 17 | 9 | 40 | 56 | -8.3 | -36 | 0 | 33 | 2 |  |
| 2020 | 11 | 20 | 17 | 44 | 48 | -8.3 | -36 | 0 | 33 | 1.8 |  |
| 2020 | 11 | 29 | 15 | 35 | 16 | -10 | -40.1 | 0 | 14 | 2.5 |  |
| 2020 | 12 | 4 | 7 | 8 | 24 | -2.8 | -44.8 | 0 | 20 | 3.7 | 4 |
| 2020 | 12 | 9 | 2 | 7 | 42 | -11 | -40.5 | 0 | 8 | 3 |  |
| 2020 | 12 | 9 | 2 | 18 | 59 | -11 | -40.5 | 0 | 8 | 3.2 |  |
| 2020 | 12 | 21 | 15 | 17 | 7 | -8.3 | -36 | 0 | 33 | 1.9 |  |
| 2020 | 12 | 24 | 9 | 10 | 51 | -8.3 | -36 | 0 | 33 | 1.7 |  |

# **References**

[1] A. Mignan, J. Woessner, Estimating the magnitude of completeness for earthquake catalogs, Community Online Resource for Statistical Seismicity Analysis, Community Online Resour. Stat. Seism. Anal. (2012). https://doi.org/10.5078/corssa-00180805.

[2] S. Wiemer, M. Wyss, Minimum Magnitude of Completeness in Earthquake Catalogs: Examples from Alaska, the Western United States, and Japan, Bull. Seismol. Soc. Am. 90 (2000) 859–869. https://doi.org/10.1785/0119990114.

[3] K.M. Leptokaropoulos, V.G. Karakostas, E.E. Papadimitriou, A.K. Adamaki, O. Tan, S. Inan, A homogeneous earthquake catalog for western Turkey and magnitude of completeness determination, Bull. Seismol. Soc. Am. (2013). https://doi.org/10.1785/0120120174.

[4] B. Orlecka-Sikora, S. Lasocki, J. Kocot, T. Szepieniec, J.R. Grasso, A. Garcia-Aristizabal, M. Schaming, P. Urban, G. Jones, I. Stimpson, S. Dineva, P. Sałek, K. Leptokaropoulos, G. Lizurek, D. Olszewska, J. Schmittbuhl, G. Kwiatek, A. Blanke, G. Saccorotti, K. Chodzińska, Ł. Rudziński, I. Dobrzycka, G. Mutke, A. Barański, A. Pierzyna, E. Kozlovskaya, J. Nevalainen, J. Kinscher, J. Sileny, M. Sterzel, S. Cielesta, T. Fischer, An open data infrastructure for the study of anthropogenic hazards linked to georesource exploitation, Sci. Data 7 (2020) 89. https://doi.org/10.1038/s41597-020-0429-3.

[5] T.W. Anderson, D.A. Darling, A Test of Goodness of Fit, J. Am. Stat. Assoc. (1954). https://doi.org/10.1080/01621459.1954.10501232.

[6] S. Lasocki, E.E. Papadimitriou, Magnitude distribution complexity revealed in seismicity from Greece, J. Geophys. Res. Solid Earth 111 (2006) n/a-n/a. https://doi.org/10.1029/2005JB003794.
